# Evaluation of Antiproliferative Activity and Molecular Modeling Studies of Some Novel Benzimidazolone-Bridged Hybrid Compounds

**DOI:** 10.3390/ph18121899

**Published:** 2025-12-17

**Authors:** Okan Güven, Emre Menteşe, Fatih Yılmaz, Adem Güner, Mustafa Emirik, Nedime Çalışkan

**Affiliations:** 1 Department of Chemistry, Faculty of Art and Sciences, Recep Tayyip Erdogan University, 53100 Rize, Turkey; okan_guven172@erdogan.edu.tr (O.G.); emre.mentese@erdogan.edu.tr (E.M.); mustafa.emirik@erdogan.edu.tr (M.E.); nedime_caliskan19@erdogan.edu.tr (N.Ç.); 2 Vocational School of Technical Sciences, Department of Chemistry and Chemical Process Technology, Recep Tayyip Erdogan University, 53100 Rize, Turkey; 3 Department of Occupational Health and Safety, Faculty of Health Sciences, Sinop University, 57000 Sinop, Turkey; ademguner@sinop.edu.tr

**Keywords:** benzimidazolone, coumarin, piperazine, oxadiazole, breast cancer, lung cancer, cervical cancer, molecular docking

## Abstract

**Background/Objectives**: Cancer is among the leading causes of mortality worldwide. In 2022 alone, the global cancer death toll stood at 9.74 million. Projections indicate that this figure will rise to 10.4 million by 2025. **Methods**: A new series of benzimidazolone-bridged hybrid compounds containing thiophene, furan, oxadiazole, piperazine, and coumarin moieties was synthesized and structurally characterized by ^1^H-NMR, ^13^C-NMR (APT), and elemental analysis. Their cytotoxic effects were evaluated by MTT assay against human lung (A549), human breast (MCF-7), and human cervical (HeLa) cancer cell lines, and the non-cancerous HEK293 cell line after 48 h exposure over a concentration range of 0.5–250 µM. IC_50_ values were determined, and Selectivity Indexes (SI) were calculated using HEK293 as the reference normal cell line. Molecular docking studies were carried out using the Glide XP protocol against VEGFR2 (PDB ID: 4ASD) and CDK4–Cyclin D3 (PDB ID: 7SJ3), with sorafenib and abemaciclib as reference inhibitors. **Results**: The results of anticancer activity were compared with doxorubicin (IC_50_ ± SD (µM)/SI: 4.3 ± 0.2/1.20 for A549, 6.4 ± 0.37/0.77 for MCF-7, 3.4 ± 0.19/1.54 for HeLa), a drug used for cancer chemotherapy. The structures of the newly synthesized hybrid compounds were identified by ^1^H-NMR, ^13^C-NMR (APT), and elemental analysis data. These hybrid compounds represent a promising class of anticancer agents. Several compounds demonstrated marked and concentration-dependent cytotoxicity across all cancer cell lines, with HeLa cells showing the highest overall sensitivity. The introduction of an oxadiazole ring (compound **7**) and coumarin substituents (compounds **12b**–**12d**) markedly improved anticancer activity and selectivity, yielding low-micromolar IC_50_ values in HeLa cells (10.6–13.6 µM) and high Selectivity Indexes (SI = 2.0–3.63). Compound **6** also exhibited balanced potency across A549, MCF-7, and HeLa cells (IC_50_ = 28.3–31.2 µM) with SI values ≥ 2.0. Compound **9** showed strong cytotoxicity across all cancer cell lines; its moderate SI values indicate lower discrimination between malignant and non-malignant cells. Taken together, these findings identified compounds **7**, **12b**–**12d**, **6**, and **12c** as the most promising benzimidazolone-based candidates, displaying both potent cytotoxicity and favorable selectivity over non-malignant HEK293 cells. **Conclusions**: Among the synthesized molecules, the oxadiazole derivative (**7**) and the coumarin-based hybrids (**12b**–**12d**) exhibited the strongest combination of cytotoxic activity and selectivity, reflected by their low IC_50_ values and high SI ratios. Notably, compound **12c** combined strong biological activity with the *highest predicted VEGFR2 affinity* in the series, highlighting it as a particularly promising scaffold. While compound **9** exhibited excellent docking scores toward both VEGFR2 and CDK4, its lower selectivity suggests a need for further structural refinement. Overall, the biological and computational findings converge to identify these benzimidazolone hybrids as credible lead candidates for future anticancer optimization.

## 1. Introduction

Breast cancer is a type of cancer that originates in the breast tissue, manifesting as an unregulated proliferation of cells. It is known as the second most prevalent cause of cancerous ailments among women [1,2]. The global incidence of breast cancer was about 2.3 million cases in 2022, and it is estimated to be 2.45 million in 2025. This will result in approximately 715,000 deaths among women in 2025. Breast cancer can affect any woman at any age after puberty. In 2025, approximately 13% of women will receive a breast cancer diagnosis during their lifetime. The mortality rate from breast cancer remained unchanged between the 1930s and the 1970s. However, an enhancement in survival rates was observed in the 1990s, attributable to the implementation of two key factors: the early detection of the condition and the introduction of effective pharmaceutical treatments [3,4,5].

Lung cancer is among the most prevalent types of cancer worldwide, with a high incidence of mortality [6,7,8]. As the disease progresses, cancerous cells can metastasize to the lungs, pancreas, stomach, and bones through a process known as angiogenesis. Angiogenesis has been identified as a contributing factor to the development of numerous diseases, including cancer. This is a significant mechanism employed by tumors to facilitate growth and dissemination. It has been demonstrated that cancerous cells can promote angiogenesis to secure a blood supply that supports their growth and allows them to survive, especially as they increase in size [9,10,11,12].

Cervical cancer is caused by a sexually acquired infection with certain types of human papillomavirus (HPV) [13]. Nearly all cases of cervical cancer are attributable to HPV infection, with smoking and HIV infection acting as contributing risk factors [14]. Around the world, cervical cancer is the second most common cancer in women, especially those living in underdeveloped regions, with an estimated 662,000 new cases in 2022, representing 6.5% of all female cancers. Among them, almost 349,000 women died from cervical cancer; it is estimated that mortality will reach 373,000 in 2025 and 545,000 in 2050 [15,16]. Therefore, there is critical importance of developing new anticancer agents with improved efficacy, selectivity, and reduced side effects.

**Figure 1:**
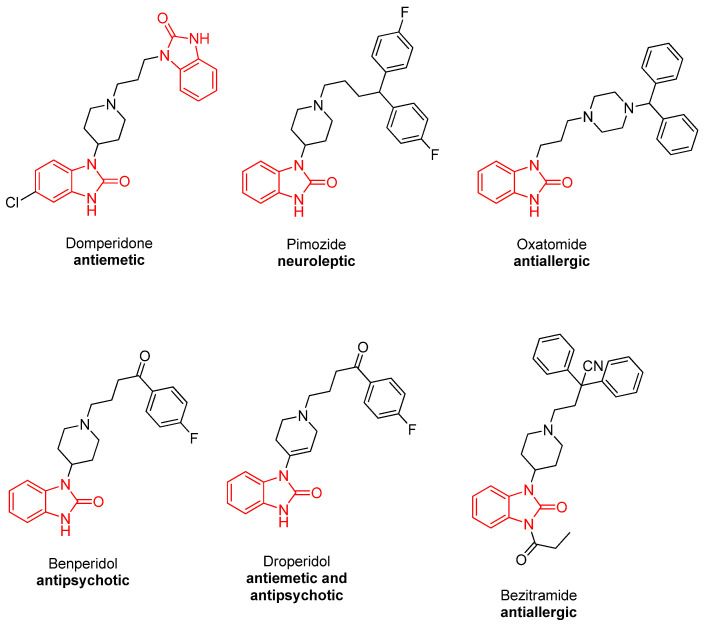
Some clinically used benzimidazolone-containing drugs.

Benzimidazolone derivatives have been shown to be efficacious in the treatment of some diseases such as mental disorders, glaucoma, obesity, and type-2 diabetes [17]. A number of studies have been conducted on benzimidazolone derivatives in the literature. These studies have demonstrated that benzimidazolone derivatives possess a wide range of pharmacological and biological properties, including anticancer [18], anti-HIV [19], antiviral [20], antidiabetic [17], and antimicrobial [21] activities. In addition, benzimidazolone cycles are found in the structures of clinically used drugs, including Domperidone (antiemetic) [22], Oxatomide (antiallergic) [23], Benperidol (antipsychotic) [24], Pimozide (neuroleptic) [25], Droperidol (antiemetic and antipsychotic) [26], and Benzitramide (antiallergic) [18] (Figure 1).

The development of effective anticancer compounds is an ongoing endeavor aimed at overcoming some of the challenges associated with existing treatments, such as severe toxicity and drug resistance [5,27,28,29,30,31,32,33,34,35,36]. Molecular hybridization is a medicinal chemistry approach that facilitates the synthesis of novel bioactive compounds. It can make new molecules more effective and increase their activity. It can also reduce side effects and help overcome drug resistance. This is because it combines two or more pharmacophores into a single structure. Many hybrid compounds have been studied for their different biological properties or have already been used in clinics to treat cancer. This demonstrates the value of this approach [37,38,39,40,41,42,43,44,45,46,47,48,49,50,51,52]. It seems that combining the benzimidazolone core with the oxadiazole, coumarin, piperazine, furan, and thiophene rings, which are known as bioactive compounds, could create new anticancer drugs that are less toxic, more specific, and more effective against both drug-sensitive and drug-resistant cancers.

The Vascular Endothelial Growth Factor Receptor 2 (VEGFR-2) and Cyclin-Dependent Kinase 4 (CDK4) are the most important therapeutic targets in cancer research because of their pivotal roles in cancer processes, angiogenesis, and uncontrolled proliferation, respectively. VEGFR-2 is the primary mediator of pro-angiogenic signaling, crucial for initiating endothelial cell migration and vessel formation necessary to sustain tumor growth and metastasis [53]. The inhibition of its tyrosine kinase activity is a clinically validated approach in multi-kinase therapy for various malignancies [54]. Concurrently, CDK4 is an indispensable component of the cell cycle machinery, driving the G1-to-S phase transition by regulating the Retinoblastoma (Rb) protein. Its aberrant activation, often through Cyclin D amplification or p16 inactivation, is a defining feature that allows cancer cells to bypass cell cycle checkpoints [55]. The clinical success of CDK4/6 inhibitors in breast cancer further validates this kinase as a high-value therapeutic target [56]. Screening novel small molecules against both VEGFR-2 and CDK4 offers a comprehensive approach to identify potential multi-targeted agents capable of simultaneously disrupting two fundamental and complementary pathways of tumor progression.

In our previous studies, we synthesized some benzimidazolone-conjugated hybrid compounds containing coumarin, piperazine, triazole, thiadiazole, furan, and thiophene moieties and investigated their urease, lipase, and acetylcholinesterase inhibitory activities [57,58]. In the literature, there are many studies on the biological activities of benzimidazole derivatives [19,59,60,61]. Based on the anticancer, antimicrobial, and enzymatic inhibitory properties of benzimidazolone derivatives, and considering the biological relevance of thiophene, furan, oxadiazole, piperazine, and coumarin moieties as pharmacologically active scaffolds, we hypothesized that hybridizing these bioactive fragments into a single benzimidazolone-centered framework would yield novel compounds with enhanced cytotoxic potency and improved selectivity toward cancer cells. Furthermore, due to their structural compatibility with VEGFR2 and CDK4 active sites, we anticipated that these hybrids would demonstrate favorable binding interactions in molecular docking studies, supporting their potential as promising anticancer lead compounds. Inspired by the literature and the lack of anticancer activity studies of benzimidazolone hybrid compounds, in this work, we have synthesized benzimidazolone-conjugated biscoumarin and piperazine and investigated their anticancer activities against breast cancer (MCF-7), lung cancer (A549), and cervical cancer (HeLa) cell lines and performed an in silico molecular docking study targeting the critical oncological enzymes VEGFR-2 and CDK4 to understand the potential mechanism of action.

## 2. Results and Discussion

### 2.1. Chemistry

**Scheme 1:**
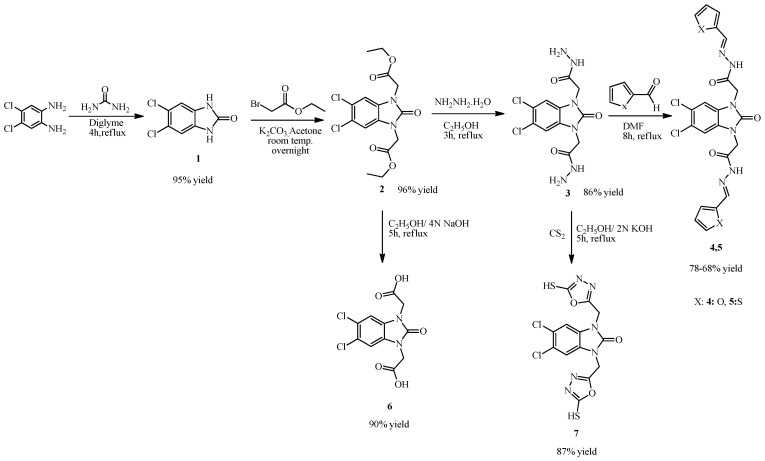
Synthesis of compounds **1**–**7**.

The synthesis studies were started by boiling 4,5-dichloro-o-phenylenediamine with urea in diglyme to obtain the starting benzimidazolone derivative (4,5-dichloro-1,3-dihydro-2*H*-benzimidazol-2-one (**1**)). Subsequently, the diacetate derivative (**2**) was synthesized via an alkylation reaction of compound **1** by reacting 4,5-dichloro-1,3-dihydro-2*H*-benzimidazol-2-one (**1**) with ethylbromo acetate in the presence of K_2_CO_3._ Then, compound **2** was converted to its diacetohydrazide derivative (**3**) by boiling compound **2** with hydrazine hydrate in absolute ethanol. Then, compound **3** was reacted with furan- and thiophene-2-carbaldehyde to obtain benzimidazolone-bridged hybrid compounds containing furan and thiophene moieties (compounds **4**, **5**) (Schiff base formation reaction). Then, compound **2** was boiled in a mixture of ethanol and 4N NaOH solution to obtain its carboxylic acid derivative, 2,2′-(5,6-dichloro-2-oxo-1*H*-benzo[d]imidazole-1,3(2*H*)-diyl)diacetic acid (**6**). Lastly, compound **3** was reacted with CS_2_ to obtain the oxadiazole derivative, 5,6-dichloro-1,3-bis((5-mercapto-1,3,4-oxadiazol-2-yl)methyl)-1*H*-benzo[d]imidazol-2(3*H*)-one (**7**) (Scheme 1).

**Scheme 2:**
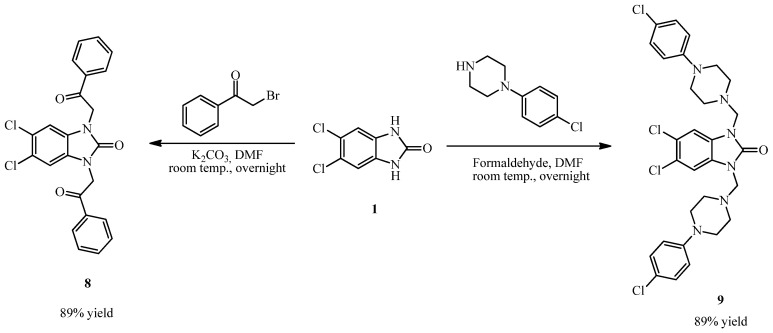
Synthesis of compounds **8**, **9**.

The reaction of compound **1** with pheny acylbromide in DMF via an alkylation reaction gave compound **8**, 5,6-dichloro-1,3-bis(2-oxo-2-phenylethyl)-1,3-dihydro-2*H*-benzimidazol-2-one, and the reaction of compound **1** with 4-chlorophenyl piperazine and formaldehyde via a Mannich reaction in DMF gave 5,6-dichloro-1,3-bis((4-(4-chlorophenyl)piperazin-1-yl)methyl)-1*H*-benzo[d]imidazol-2(3*H*)-one (**9**) (Scheme 2).

**Scheme 3:**
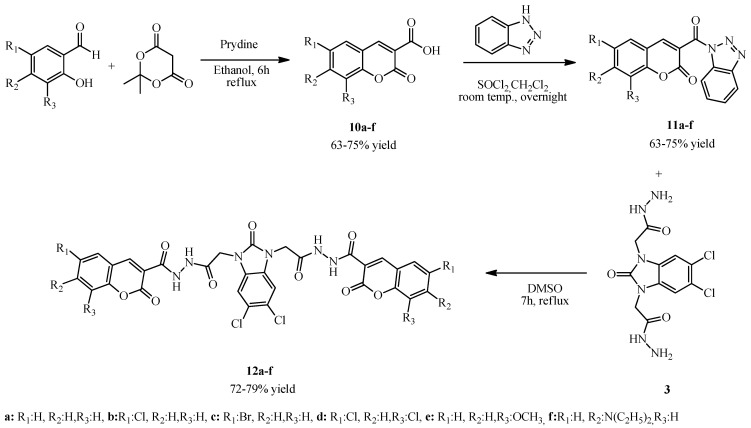
Synthesis of compounds **12a**–**f**.

To obtain coumarin-containing hybrid compounds, coumarin derivatives were synthesized according to our published protocols. Firstly, salicyl aldehyde derivatives were reacted with 2,2-dimethyl-1,3-dioxane-4,6-dione (Meldrum’s acid) in ethanol containing a catalytic amount of pyridine to obtain coumarin-3-carboxylic acid derivatives (**10a**–**f**) (Knoevenage**l** condensation). Our literature review has shown that benzotriazole is a good leaving group and can facilitate many chemical transformations. Therefore, we reacted compounds **10a**–**f** with 1*H*-benzotriazole in dichloromethane in the presence of SOCl_2_ to obtain coumarin-containing benzotriazole derivatives (**11a**–**f**) (first by formation of coumarin acyl chloride and second by substitution of chlorine and hydrogen of benzotriazole). Then, compounds **11a**–**f** were reacted with compound **3** to obtain benzimidazolone-bridged biscoumarin derivatives (**12a**–**f**) (nucleophilic reaction of hydrazide with coumarin-benzotriazole derivatives resulted in departure of benzotriazole and formation of new hydrazide derivatives) (Scheme 3).

Spectral investigations of newly synthesized compounds are in accordance with the proposed structures. In the ^1^H-NMR spectra of compound **2**, formation of new CH_3_, OCH_2_, and NCH_2_ signals at 1.91, 4.15, and 4.74 ppm, respectively, and disappearance of the NH signal (at 10.89 ppm in the ^1^H-NMR spectra of compound **1**) demonstrated the alkylation reaction. In the ^13^C-NMR spectra of compound **2**, CH_3_, OCH_2_, NCH_2_, and C=O signals were observed at 14.90, 42.50, 61.58, and 166.94 ppm. In the ^1^H-NMR spectra of compound **3**, disappearance of CH_3_ and OCH_2_ signals (coming from compound **2**) and formation of new NH_2_ and NH signals at 4.29 + 4.74 and 9.30 + 8.68 ppm demonstrated hydrazide formation. In the ^13^C-NMR spectra of compound **3**, disappearance of CH_3_ and OCH_2_ (coming from compound **2**) demonstrated hydrazide formation. In the ^1^H-NMR spectra of compounds **4** and **5**, disappearance of the NH_2_ signal (coming from compound **3**) and the formation of new N=CH and aromatic proton signals at about 8.21 and 7.14–7.65 ppm demonstrated Schiff base formation. In the ^13^C-NMR spectra of these compounds, formation of N=C**H** and aromatic protons at about a 145 and 112–150 ppm, respectively, demonstrated Schiff base formation.

**Scheme 4:**
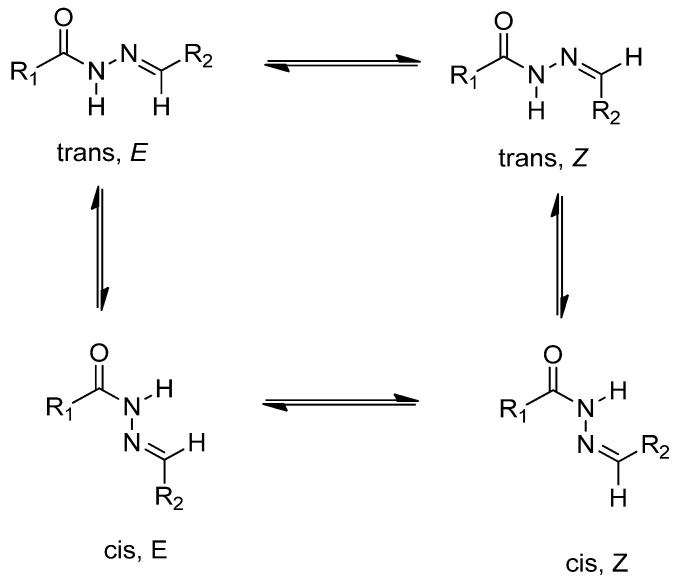
*E*/*Z* geometrical isomers and cis/trans amide conformer of compounds **4** and **5**.

In the ^1^H-NMR and ^13^C-NMR spectra of compounds **3**–**5**, it was seen that some of the protons of these compounds have two sets of signals at different ppm. This is because the compounds, which have an arylene-hydrazide structure, exist as *E*/*Z* geometrical isomers of the C═N double bond and *cis*/*trans* amide conformers at the CO–NH single bond. According to the literature [62,63,64,65], compounds that have a C═N double bond prefer the *E* geometrical isomers in DMSO-*d*_6_, and *Z* isomers can be preferred in less polar solvents. N–CH_2_ and N–H signals were observed in two sets of signals due to *cis*/*trans* conformers. The ratio in each case was calculated by using ^1^H-NMR data. *E*/*Z* and *cis*/*trans* geometrical isomer of compounds **4** and **5** are shown in Scheme 4.

In the ^1^H-NMR spectra of compound **6**, disappearance of OCH_2_ and CH_3_ signals (from compound **2**) and the formation of new COOH signal at 13.16 ppm demonstrated carboxylic acid formation. In the ^13^C-NMR spectra of this compound, OCH_2_ and CH_3_ signals (from compound **2**) disappeared. In the ^1^H-NMR spectra of compound **7**, NH and NH_2_ signals (coming from compound **3**) disappeared, and in the ^13^C NMR spectra of this compound, the C=S signal was shown at 177.11 ppm. The other aromatic carbons appeared in the appropriate region.

In the ^1^H-NMR spectra of compound **8**, the protons belonging to NCH_2_ and the benzene cycle appeared at about 5.57 ppm and 7.50–8.00 ppm, respectively. In the ^13^C-NMR spectra of this compound, C=O and NCH_2_ signals appeared at 48.28 and 163.47 ppm, respectively. In the ^1^H-NMR spectra of compound **9**, CH_2_ signals originating from piperazine appeared at 2.68 and 3.07 ppm. The NCH_2_ signal of this compound appeared at 4.70 ppm. In the ^13^C-NMR spectra of this compound, piperazine CH_2_ carbons appeared at 48.39 and 50.14 ppm, and the NCH_2_ carbon appeared at 62.73 ppm. Other aromatic carbons resonated in the appropriate range in the ^13^C-NMR spectra of this compound.

In the ^1^H-NMR spectra of compounds **12a**–**f**, disappearance of the NH_2_ signal (coming from compound **3**) and the formation of a new NH signal at about 11.20 ppm, as well as the formation of a new signal at about 8.90 ppm (coming from coumarin C_3_H), demonstrated the hybridization. In the aromatic region, the signals belonging to the coumarin cycle were clearly observed. In the ^13^C-NMR spectra of these compounds, new C=O signals coming from the coumarin ring appeared at about 159 and 160 ppm. The coumarin C_4_H proton appeared at about 148 ppm. In addition, all compounds showed suitable elemental analysis results.

### 2.2. Cell Viability

**Figure 2:**
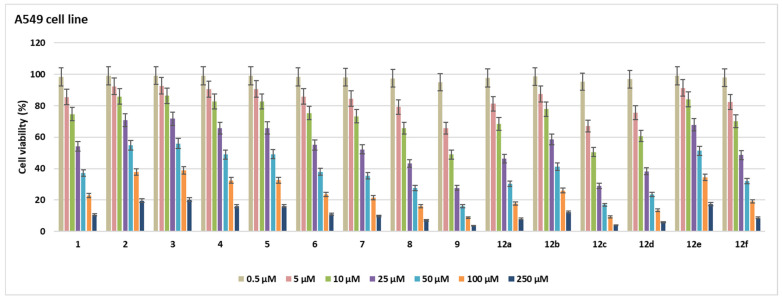
Cell viability (%) in A549 cell lines after treatment with the compounds for 48 h at seven different concentrations (0.5–250 µM).

**Figure 3:**
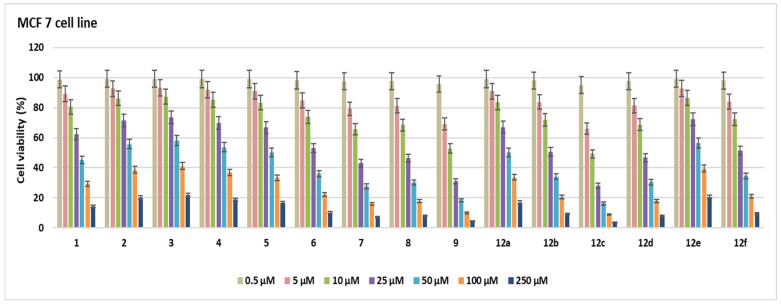
Cell viability (%) in MCF-7 cell lines after treatment with the compounds for 48 h at seven different concentrations (0.5–250 µM).

**Figure 4:**
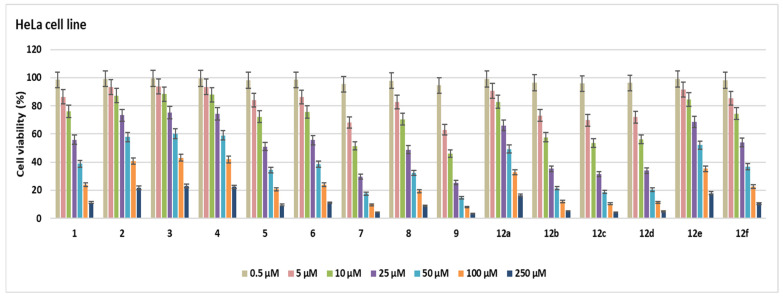
Cell viability (%) in HeLa cell lines after treatment with the compounds for 48 h at seven different concentrations (0.5–250 µM).

**Figure 5:**
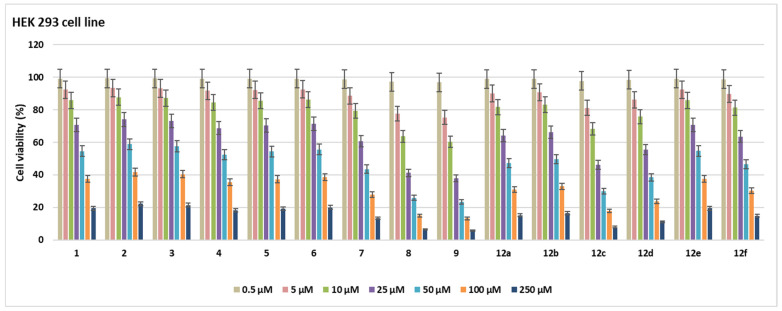
Cell viability (%) in HEK 293 cell lines after treatment with the compounds for 48 h at seven different concentrations (0.5–250 µM).

**Figure 6:**
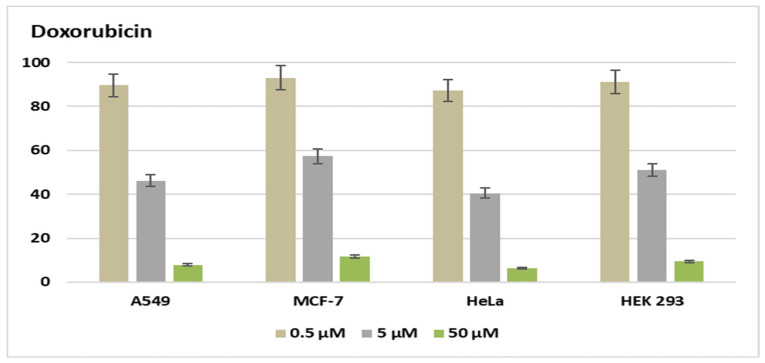
Cell viability (%) of doxorubicin in A549 MCF-7, HeLa, and HEK 293 at three different concentrations (0.5–50 µM).

In this study, the cytotoxic activities of 15 novel compounds were assessed across A549, MCF-7, and HeLa cell lines, as well as the non-cancerous HEK 293 cell line. IC_50_ values (µM ± SD) and Selectivity Indexes (SI) were calculated based on HEK 293 as the reference non-malignant cell line (Table 1, Figure 2, Figure 3, Figure 4, Figure 5 and Figure 6).

**Figure 7:**
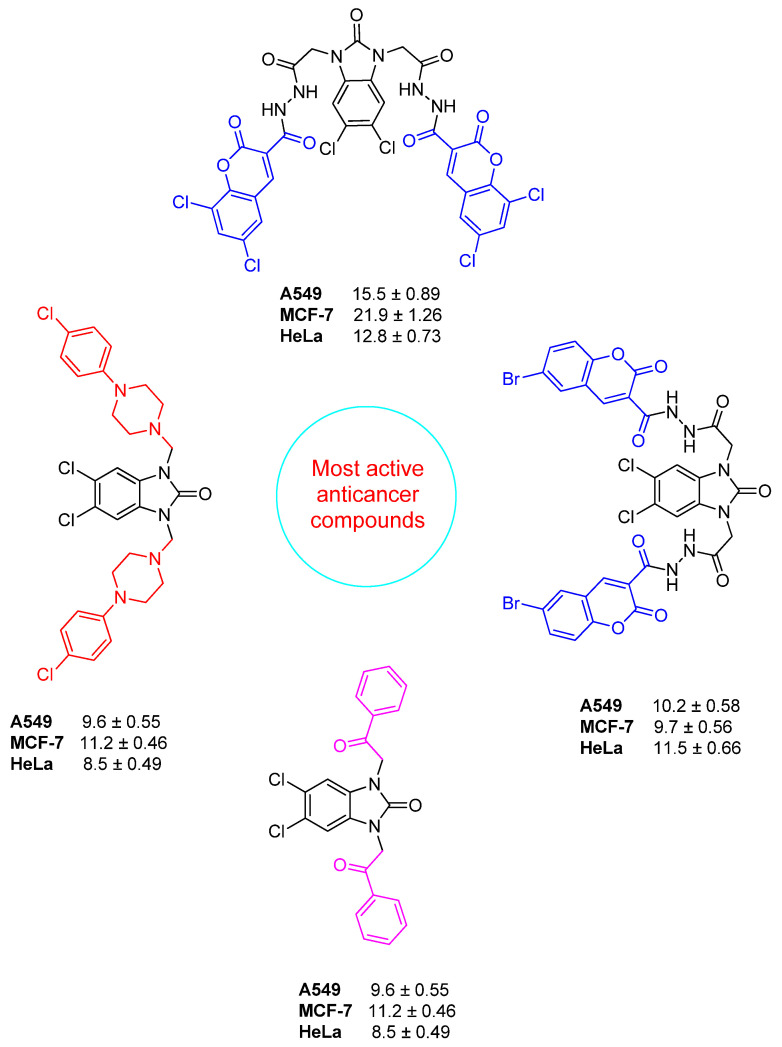
Promising benzimidazolone hybrids with potential anticancer activity.

Compound **6** exhibited notable cytotoxicity with IC_50_ values of 30.6 ± 1.76 µM in A549, 28.3 ± 1.63 µM in MCF-7, and 31.2 ± 1.8 µM in HeLa cells, while showing reduced toxicity in HEK 293 cells (62.7 ± 3.62 µM). The SI values (2.04, 2.21, and 2.00, respectively) indicated selective activity across all cancer cell lines. Compound **5** showed higher selectivity against HeLa cells (IC_50_ = 26.1 ± 1.5 µM) compared to A549 (48.3 ± 2.79 µM) and MCF-7 (50.2 ± 2.9 µM), resulting in a notable SI of 2.27 for HeLa. Compound **7** was most potent in HeLa cells (10.6 ± 0.61 µM, SI = 3.63), while also showing significant cytotoxicity in MCF-7 (19.1 ± 1.1 µM, SI = 2.01) and A549 (27.4 ± 1.58 µM, SI = 1.4), highlighting its HeLa-specific activity. Compound 12a showed strong cytotoxicity against A549 (21.7 ± 1.25 µM, SI = 2.05), but lower SI values were found for MCF-7 (50.8 ± 2.93 µM) and HeLa (48.5 ± 2.8 µM) (SI < 1), which means it was less selective. Compounds 2–4 and 12e had relatively high IC_50_ values across all tested cell lines, with SI values close to or below 1.2. This means that they were not very cytotoxic and had limited selectivity. Compounds **12b** and **12d** were notably potent against HeLa cells, with IC_50_ values of 13.6 ± 0.78 µM (SI = 3.61) and 12.8 ± 0.73 µM (SI = 2.4), respectively, indicating selective cytotoxicity against cervical cancer cells. Compound **9** showed strong activity in A549 (9.6 ± 0.55 µM), MCF-7 (11.2 ± 0.46 µM), and HeLa (8.5 ± 0.49 µM) cells; however, its relatively low SI values (1.35–1.78) suggest that the compound also exhibited comparable cytotoxicity in non-cancerous HEK 293 cells (15.2 ± 0.87 µM), limiting its therapeutic selectivity. Compound **8** exhibited cytotoxic activity in A549 (19.1 ± 1.1 µM), MCF-7 (21.7 ± 1.25 µM), and HeLa (23.9 ± 1.38 µM), but low SI values (0.73–0.91) and comparable toxicity in HEK 293 cells (17.5 ± 1.01 µM) suggest a lack of cancer-specific selectivity. Compound 1 exhibited moderate cytotoxicity, with IC_50_ values of 29.5 ± 1.7 µM (SI = 2.03) in A549 cells and 31.6 ± 1.82 µM (SI = 1.9) in HeLa cells, indicating a moderate selectivity for these cancer cell lines. Compound **12f** exhibited cytotoxicity in A549 (23.6 ± 1.3 µM), MCF-7 (26.4 ± 1.52 µM), and HeLa (29.1 ± 1.26 µM), with HEK 293 IC_50_ at 43.5 ± 2.51 µM. SI values ranged from 1.49 to 1.84, indicating moderate selectivity. Compound **12c** was cytotoxic against A549 (10.2 ± 0.58 µM), MCF-7 (9.7 ± 0.56 µM), and HeLa (11.5 ± 0.66 µM), with SI values of 2.01, 2.21, and 1.86, respectively, based on its IC_50_ in HEK 293 cells (21.5 ± 1.24 µM). Doxorubicin showed strong cytotoxicity with IC_50_ values of 4.3 ± 0.2 µM (A549), 6.4 ± 0.37 µM (MCF-7), 3.4 ± 0.19 µM (HeLa), and 5.2 ± 0.3 µM (HEK 293), corresponding to SI values between 0.77 and 1.54. The most active compounds are shown in Figure 7.

The biological evaluation of the synthesized benzimidazolone-based hybrid compounds revealed clear structure–activity relationships, demonstrating how specific heterocyclic fragments modulate anticancer potency and selectivity. Overall, the introduction of heteroaromatic rings (furan, thiophene, oxadiazole), piperazine, and coumarin scaffolds significantly enhanced cytotoxicity compared to the parent benzimidazolone core (compound **1**). Among the series, the oxadiazole-bearing compound **7** demonstrated the most potent and selective activity toward HeLa cells (IC_50_ = 10.6 µM, SI = 3.63). This is in line with literature reports where the incorporation of 1,3,4-oxadiazole rings enhances antiproliferative activity by improving metabolic stability and facilitating interaction with the hinge region of kinases.

The piperazine-containing compound **9** showed strong activity across all cancer cell lines (IC_50_ = 8.5–11.2 µM). Piperazine linkers are well-known pharmacophores in kinase-targeting small molecules due to their ability to improve aqueous solubility and enhance receptor binding through cation–π and H-bond interactions. Recent benzimidazole–piperazine conjugates have demonstrated IC_50_ values around 10–30 µM against MCF-7 and A549, indicating that compound **9** performs at the upper end of the potency reported for structurally similar molecules. However, its moderate selectivity index (SI < 2) suggests that further substitution on the aromatic moieties may be necessary to balance potency and tumor specificity.

The coumarin-based derivatives (**12b**–**12d**) displayed pronounced cytotoxicity, especially against HeLa cells (IC_50_ = 12.8–13.6 µM), with high SI values (2.4–3.61). Coumarin hybrids have frequently been associated with enhanced antiproliferative activity due to their planar aromatic structure and ability to π–stack with hydrophobic pockets in VEGFR2 and CDK4.

Comparing the entire set to the reference drug doxorubicin, several synthesized compounds exhibited higher Selectivity Indices (SI > 2), whereas doxorubicin displayed SI < 1.6 across all tested cell lines. This demonstrates that although the absolute cytotoxic potency of some compounds is weaker than that of doxorubicin, their selectivity toward cancer cells is superior, an important feature for reducing off-target toxicity.

### 2.3. Biochemical Analyses

Following the cell viability screening, only the compounds demonstrating selective cytotoxicity (SI > 2 in at least one tumor cell line), together with strong molecular docking affinity toward VEGFR2 and/or CDK4 were advanced to redox-related LDH, TOS, and GSH/GSSG assays. Based on this dual-criterion approach, compounds **5**, **6**, **7**, **12b**, **12c**, **12d**, and **1** were identified as the most promising candidates.

Among these, compounds **6**, **7**, **12c**, and **12d** showed the most consistent activity across multiple cell lines and exhibited potent binding energies (VEGFR2: −10.5 to −13.4 kcal/mol; CDK4: −6.5 to −8.6 kcal/mol). In particular, compound **7** displayed strong dual-target affinity and high selectivity toward HeLa cells; **12c** showed the best VEGFR2 interaction within the whole series; **12d** demonstrated HeLa- and A549-specific selectivity; and compound **6** exerted balanced cytotoxicity in A549, MCF-7, and HeLa cells. These combined features justified their progression into oxidative stress-related biochemical analysis.

#### 2.3.1. LDH Assay

LDH release sharply increased in all treatment groups, indicating significant plasma membrane disruption, a hallmark of late apoptosis or secondary necrosis (Table 2). In HeLa cells, compound **7** caused the greatest LDH leakage (30.9 ± 2.7 µU/mL ^c^) compared to the control (12.4 ± 1.1), consistent with its strong dual-target docking affinity and high SI value. Similarly, compound **12d** (29.7 ± 2.4) and **12b** (28.5 ± 2.3) produced pronounced LDH elevations compared to the control, supporting their ability to drive apoptotic progression toward terminal stages. Moderate but significant increases were also observed with compound **6** (27.3 ± 2.5) and compound **5** (24.8 ± 2.1) compared to the control. In A549 cells, compound **12c** generated the most substantial LDH release (30.4 ± 2.6 µU/mL) compared to the other compounds, consistent with its lowest IC_50_ value and strongest VEGFR2 interaction. This was followed by **12d** (27.3 ± 2.2) and compound **6** (24.5 ± 2.0), both of which produced moderate but significant increases compared to the control (10.9 ± 1.0). Compound **1** (19.8 ± 1.7) caused a milder yet statistically significant elevation. In MCF-7 cells, LDH levels followed the rank **12c** > **7** > **6**, with **12c** showing the highest release (28.8 ± 2.2), followed by compound 7 (25.3 ± 2.0) and compound **6** (22.7 ± 1.8), all significantly elevated compared to the control (11.6 ± 1.0, *p* < 0.05). This pattern closely parallels their cytotoxicity profiles and confirms that LDH leakage is tightly linked to apoptosis intensity. Taken together, LDH results showed that the selected compounds promote late apoptotic events in a potency-dependent manner, with the strongest effects seen in compound **7** (HeLa) and compound **12c** (A549 and MCF-7) compared to their respective controls.

#### 2.3.2. Total Oxidative Stress (TOS)

Tested compounds significantly increased total oxidative status compared to the control in cell lines. In HeLa cells, compound **7** produced the highest TOS level (8.6 ± 0.7 μmol H_2_O_2_ eq/L) compared to the control (3.1 ± 0.3), indicating a robust induction of oxidative stress. Similar but slightly lower increases were observed with compound **12d** (8.4 ± 0.6) and **12b** (8.1 ± 0.6) compared to the control, both reflecting strong ROS-generating capacity. Moderate elevations were recorded for compound **6** (7.8 ± 0.6) and compound **5** (6.9 ± 0.5) relative to the control. In A549 cells, compound **12c** induced the strongest TOS elevation (8.5 ± 0.7 ^c^) compared to the other compounds and to the control (3.4 ± 0.4), consistent with its potent cytotoxicity and highest VEGFR2 docking affinity. Compound **12d** (7.9 ± 0.6) and compound **6** (7.1 ± 0.5) also produced significant oxidative increases compared to the control, while compound 1 (6.1 ± 0.4) showed a smaller but still statistically significant rise (*p* < 0.05). In MCF-7 cells, TOS followed the order **12c** > **7** > **6**, with compound **12c** displaying the highest TOS level (8.3 ± 0.6) compared to the control (3.0 ± 0.3), followed by compound **7** (7.6 ± 0.6) and compound **6** (6.9 ± 0.5). These findings align with the LDH and viability data, indicating that oxidative stress is a central driver of cytotoxicity in these lines. Overall, TOS results confirm that the most potent VEGFR2-interacting compounds, particularly **7** and **12c**, generate a pronounced oxidative environment compared to the other compounds and respective controls.

#### 2.3.3. Glutathione Redox Imbalance (GSH/GSSG)

A marked reduction in the GSH/GSSG ratio was observed across all treatment groups compared to the control (*p* < 0.05), indicating depletion of cellular antioxidant reserves and a shift toward an oxidized intracellular state. In HeLa cells, compound **7** induced the most pronounced decrease in the GSH/GSSG ratio (9.4 ± 1.0) compared to the control (31.2 ± 2.4), indicating severe oxidative burden. Compound **12d** (9.7 ± 1.0) and **12b** (10.1 ± 1.1) displayed similarly strong reductions. Moderate decreases were recorded for compound **6** (10.6 ± 1.1) and compound **5** (11.8 ± 1.3) compared to the control. In A549 cells, compound **12c** caused the greatest reduction (9.5 ± 0.8) compared to the control (29.8 ± 2.1) and to the other compounds, mirroring its high TOS levels. Compound **12d** (10.1 ± 0.9) and compound **6** (11.2 ± 1.0) also significantly lowered the GSH/GSSG ratio compared to the control, whereas compound **1** (13.5 ± 1.3) produced a milder but still statistically meaningful decrease. In MCF-7 cells, compound **12c** again led to the most prominent decline (10.2 ± 1.0) compared to the control (33.4 ± 2.5), followed by compound **7** (11.3 ± 1.0) and compound **6** (12.1 ± 1.2). This pattern parallels the TOS and LDH results, indicating a coordinated ROS-driven apoptotic mechanism. Taken together, the decline in GSH/GSSG indicates that all tested compounds disrupt intracellular thiol homeostasis; however, compounds **7** and **12c** produce the most severe redox imbalance compared to the other groups, consistent with their potent pro-oxidant and pro-apoptotic effects.

### 2.4. Cell Cycle Analysis

**Figure 8:**
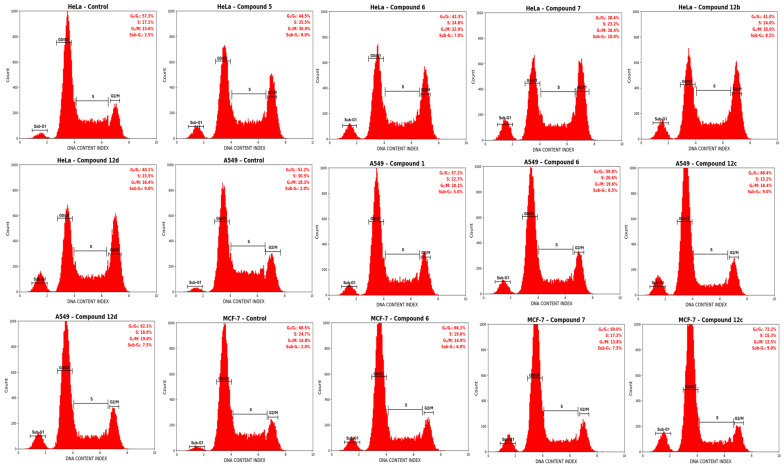
Cell cycle distribution analysis in HeLa, A549, and MCF-7 cells after 48 h treatment with tested complexes. Flow cytometric analysis of DNA content following 48 h exposure of cells to the IC_50_ concentrations of related complexes. Representative histograms illustrate the percentage of cells in the G_0_/G_1_, S, and G_2_/M phases after propidium iodide (PI) staining. The bar chart (right) summarizes the quantitative distribution of cell cycle phases expressed as mean ± SEM (n = 3).

In line with the cytotoxicity and redox data, treatment with the selected compounds caused a marked, cell line–dependent disruption of cell cycle progression compared to the control (Figure 8).

In HeLa cells, the control group displayed a typical distribution of 57.3 ± 2.0% in G0/G1, 27.1 ± 1.6% in S, and 15.6 ± 1.3% in G2/M. Exposure to compound **7** markedly reduced the G0/G1 population to 38.4 ± 2.1% and S-phase to 23.2 ± 1.5%, while increasing the G2/M fraction to 38.4 ± 1.9% compared to the control, indicating a pronounced G2/M arrest. Similarly, compound **12d** decreased G0/G1 to 40.1 ± 2.0% and increased G2/M to 36.4 ± 1.8%, whereas compound **12b** yielded 41.0 ± 2.1% G0/G1, 24.0 ± 1.4% S, and 35.0 ± 1.7% G2/M. More moderate but still significant shifts were observed with compound **6** (42.3 ± 2.3% G0/G1, 24.8 ± 1.5% S, 32.9 ± 1.6% G2/M) and compound **5** (44.5 ± 2.2% G0/G1, 25.5 ± 1.6% S, 30.0 ± 1.5% G2/M) compared to the control.

In A549 cells, the control group showed 51.2 ± 1.8% in G0/G1, 30.5 ± 1.7% in S, and 18.3 ± 1.4% in G2/M. Compound **12c** exerted the strongest effect, increasing the G0/G1 fraction to 68.4 ± 2.4% and reducing S-phase and G2/M to 15.2 ± 1.3% and 16.4 ± 1.5%, respectively, compared to the control. Compound **12d** increased G0/G1 to 62.1 ± 2.1% (S: 18.9 ± 1.4%, G2/M: 19.0 ± 1.5%) and compound **6** to 59.8 ± 2.0% (S: 20.6 ± 1.5%, G2/M: 19.6 ± 1.4%) compared to the control. In contrast, compound **1** induced a more modest G0/G1 accumulation (57.2 ± 1.9%) with S and G2/M fractions of 22.7 ± 1.5% and 20.1 ± 1.5%, respectively. This G0/G1 predominance is compatible with interference in CDK4-dependent G1–S transition and agrees with the stronger docking profile and IC_50_ of compound **12c**.

In MCF-7 cells, the control distribution consisted of 60.5 ± 2.1% G0/G1, 24.7 ± 1.5% S, and 14.8 ± 1.3% G2/M. Compound **12c** again caused the most pronounced alteration, increasing G0/G1 to 72.2 ± 2.3% and decreasing S-phase to 15.3 ± 1.2% and G2/M to 12.5 ± 1.1% compared to the control. Compound **7** resulted in 69.0 ± 2.0% G0/G1, 17.2 ± 1.3% S, and 13.8 ± 1.2% G2/M, whereas compound **6** led to a milder but significant shift with 66.1 ± 1.9% G0/G1, 19.0 ± 1.4% S, and 14.9 ± 1.3% G2/M versus the control. Overall, these data indicate that compounds **7** and **12c** predominantly induce G2/M arrest in HeLa and G0/G1 arrest in A549 and MCF-7 cells, in line with their stronger cytotoxic and docking profiles.

### 2.5. Molecular Docking Study

Comparative molecular docking analysis aimed to evaluate the inhibitory potential of a set of novel compounds against two different kinase targets, VEGFR2 (PDB ID: 4ASD) and CDK4 (PDB ID: 7SJ3), and the results were quantified using the Glide XP scoring function (Table 3). The established kinase inhibitors sorafenib and abemaciclib were used as reference compounds, yielding high binding scores of −14.66 kcal/mol and −13.60 kcal/mol for VEGFR2 and CDK4, respectively. Overall, the designed compounds exhibited promising dual-target affinity, with several molecules achieving scores similar to those of the reference drugs on either target. Notably, Compound **9** displayed strong predicted binding affinity for both receptors, achieving a docking score of −12.92 kcal/mol against VEGFR2 and an exceptionally potent score of −10.02 kcal/mol against CDK4, suggesting that it functions as a high-affinity dual inhibitor. Furthermore, compound **12c** emerged as the most potent predicted inhibitor of VEGFR2 within the new compound set, showing a superior docking score of −13.44 kcal/mol, which was particularly competitive with the reference ligand sorafenib (−14.66 kcal/mol). However, the affinity of Compound **12c** for CDK4 was significantly lower (−6.96 kcal/mol), suggesting a strong preference for the VEGFR2 active site. These data highlight the distinct selectivity profile in the series of compounds, identifying Compound **9** as a broad-spectrum dual inhibitor candidate and Compound **12c** as a highly selective lead for VEGFR2 inhibition.

**Figure 9:**
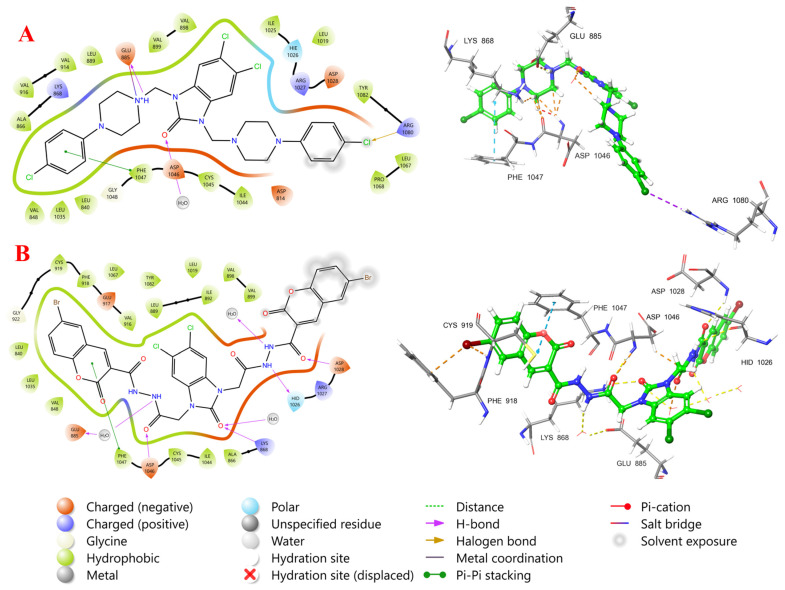
Two-dimensional and three-dimensional interaction diagrams of compound **9** (**A**) and **12c** (**B**) at the active site of VEGFR2.

Compound **9** demonstrated a high predicted binding affinity for VEGFR2 (PDB ID: 4ASD), as evidenced by docking scores of 12.92 kcal/mol. The 2D interaction diagram elucidates the key binding poses and molecular interactions stabilizing compound **9** within the ATP-binding site of VEGFR2 (Figure 9A). The binding mode and molecular interactions of compound **9** with VEGFR2 reveal a multifaceted binding profile within the ATP-binding pocket, indicative of strong inhibitory potential. The compound forms a key hydrogen bond between the protonated amine group and the negatively charged Glu885, reinforcing its anchoring in the hinge regions. A halogen bond is also observed between the terminal chlorine atom of the ligand and Arg1080, suggesting enhanced binding specificity. Additionally, the aromatic rings of the ligand participate in π–π stacking interactions with Phe1047, further stabilizing the binding. The ligand is enclosed by hydrophobic residues (e.g., Val848, Leu840, Leu1035, Val916), contributing to hydrophobic packing, while polar and charged residues such as Asp814, Arg1027, and Asp1028 flank the binding site, potentially influencing electrostatic complementarity. The overall binding conformation and interaction pattern underscore the compound’s capacity to effectively occupy and inhibit the VEGFR2 active site, highlighting its promise as a kinase inhibitor scaffold. Importantly, although these interactions support the compound’s strong predicted inhibitory affinity toward VEGFR2, the docking results do not imply cancer-selective cytotoxicity. This observation is consistent with the biological data, which show that Compound **9** is potent but exhibits low selectivity (SI < 2), and, therefore, its molecular docking profile explains potency rather than therapeutic selectivity.

The binding mode of compound **12c** within the ATP-binding site of the VEGFR-2 receptor (Figure 9B) reveals extensive and well-organized interactions that contribute to its high affinity and selectivity. The ligand occupies both the front cleft (adenine region) and extends into the back cleft (DFG motif region), indicative of a type II kinase inhibitor binding mode [66]. Notably, compound **12c** adopts a DFG-out conformation, evidenced by strong hydrogen bonding between the carbonyl group of the ligand and the conserved Asp1046 (part of the DFG motif), along with π–π stacking interactions with Phe1047, which stabilize the binding in the DFG-out pocket. In the hinge region, the compound forms a critical hydrogen bond with Glu885, mediated via a bridging water molecule, further anchoring it in the ATP site. Additional polar contacts are observed between the ligait is oknd and His1026, Asp1028, and Arg1027, located in the extended allosteric back pocket, which are key determinants of specificity for type II inhibitors. Importantly, the gatekeeper residue Val916 forms part of the hydrophobic pocket that accommodates the ligand’s bulky aromatic moiety, while other surrounding hydrophobic residues such as Leu840, Leu1035, Val848, and Ile892 contribute to shape complementarity and van der Waals stabilization. The overall binding pose of compound **12c** confirms its capacity to span both the ATP and allosteric sites, leveraging hinge, DFG, and hydrophobic pocket interactions, making it a promising scaffold for selective VEGFR-2 inhibition [67].

**Figure 10:**
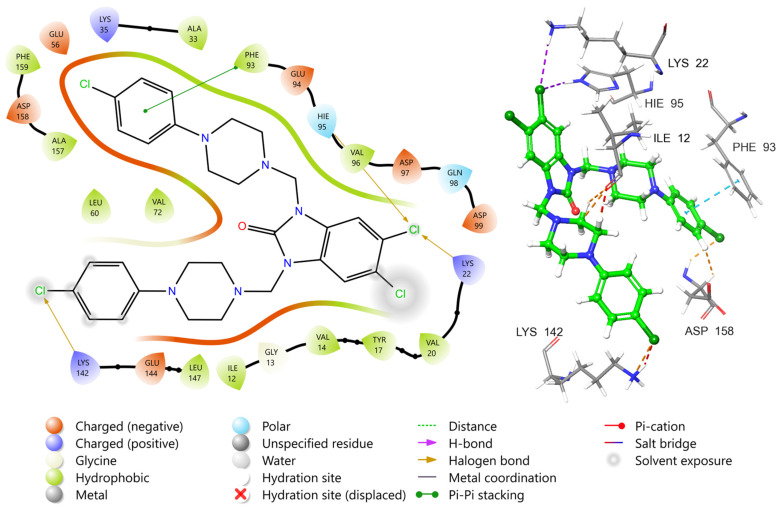
Two-dimensional and three-dimensional interaction diagrams of compound **9** at the active site of CDK4.

The binding mode of compound **9** within the CDK4–Cyclin D3 complex active site reveals that the ligand is well-anchored within the ATP-binding pocket, engaging in a network of non-covalent interactions that stabilize its conformation (Figure 10). A key feature of this interaction is the halogen bond formed between one of the terminal chlorine atoms and Lys22, which plays a critical role in ligand orientation and specificity [68]. Hydrophobic interactions also dominate the binding environment, as the ligand is deeply embedded within a hydrophobic pocket formed by residues such as Val96, Val20, Val14, Ile12, Leu147, and Tyr17. These residues stabilize the aromatic and aliphatic moieties of the ligand through van der Waals forces, enhancing complementarity within the ATP site. Notably, π–π stacking is observed between the aromatic core of the ligand and Phe93, which adds further stabilization, particularly within the adenine binding region. Furthermore, halogen bonding interactions are formed between the aromatic rings and Lys142, which sits at the entrance of the ATP site, helping to anchor the ligand at the binding cleft. Taken together, compound **9** demonstrates a robust binding pose within the CDK4 active site, stabilized by a balance of halogen bonding, π–π interactions, and extensive hydrophobic contacts. These features underscore its potential as a well-positioned ATP-competitive inhibitor, capable of selective CDK4 inhibition through engagement with both conserved and variable residues across the ATP-binding cleft.

Molecular docking analyses strongly support the experimental outcomes. Compounds **9** and **12c** formed multiple stabilizing interactions within the VEGFR2 and CDK4 active sites, such as π–π stacking with Phe1047 and key hydrogen bonds with Glu885 and Asp1046. The predicted VEGFR2 affinity of **12c** (–13.44 kcal/mol) is notably close to that of sorafenib (–14.66 kcal/mol), aligning with its high biological activity. The correlation between docking scores and cytotoxic outcomes further strengthens the hypothesis that VEGFR2 and CDK4 may be relevant molecular targets for these hybrids.

Collectively, the present findings demonstrate that the rational introduction of heterocyclic pharmacophores onto the benzimidazolone scaffold significantly enhances anticancer potential, yielding derivatives (**7**, **9**, **12c**, **12d**) that compare favorably with previously reported benzimidazole/benzimidazolone analogues. These results highlight the promising therapeutic potential of these compounds and provide a valuable starting point for further optimization toward potent, selective anticancer lead molecules.

### 2.6. ADMET Predictions

The absorption, distribution, metabolism, excretion, and toxicity (ADMET) properties of the synthesized lead candidates were computationally evaluated using the QikProp module of the Schrödinger Maestro program to predict their pharmacokinetic viability and safety profiles (Table 4). This in silico assessment was conducted to ensure that the active compounds possessed favorable properties for potential clinical applications. The compounds **9** and **12c** exhibit several encouraging properties, positioning them as promising drug leads. Both candidates show an exceptionally low propensity for hERG-related cardiotoxicity, as indicated by their highly favorable QPlogHERG values (−8.334 for **9** and −8.179 for **12c**), which suggests a high degree of cardiovascular safety. Furthermore, compound **9** demonstrates robust intestinal permeability with a QPPCaco value of 291.271 nm/s, correlating with a high predicted Percent Human Oral Absorption (HOA%) of 83.644% supporting a convenient oral dosage form. Meanwhile, compound **12c** boasts a highly desirable low predicted penetration across the blood–brain barrier (QPlogBB of −3.935), making it a highly selective candidate for peripheral targets and minimizing potential central nervous system side effects. The collective presence of a low cardiotoxicity risk across both compounds, coupled with the high oral absorption of compound **9** and the tissue selectivity of compound **12c**, strongly validates their consideration as suitable scaffolds for further anticancer optimization.

### 2.7. Structure-Activity Relationships (SAR Study)

**Figure 11:**
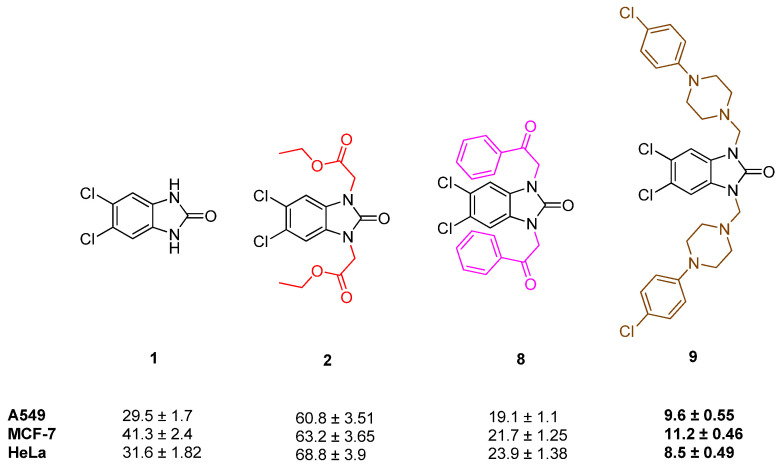
Structure and activity of compounds **1**, **2**, **8**, and **9**.

Considering the anticancer activity results, it was observed that 5,6-dichloro-1,3-dihydro-2*H*-benzimidazol-2-one (**1**) showed greater anticancer activity than its ester (compound **2**) and acetohydrazide (compound **3**) derivatives against A549, MCF-7, and HeLa cell lines. This indicates that alkylation of two NH protons in compound **1** with ethyl bromoacetate (compound **2**) resulted in decreased activity, whereas alkylation with phenyl acyl bromide (compound **6**) and 4-chlorophenyl piperazine (compound **9**) resulted in increased activity (Figure 11).

**Figure 12:**
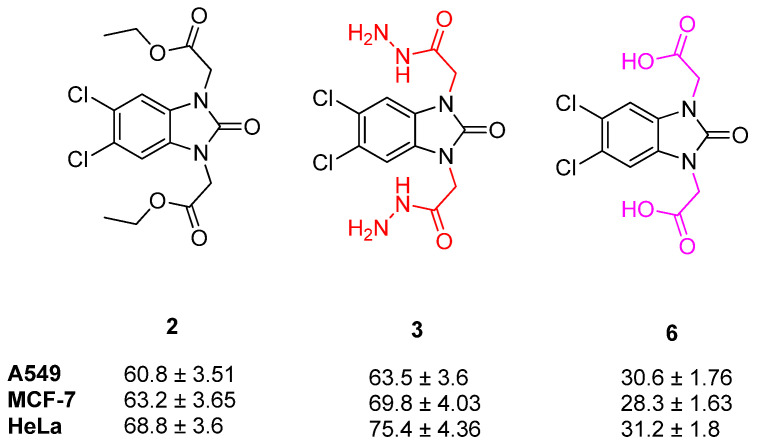
Structure and activity of compounds **2**, **3** and **6**.

When the activity of compound **2** was compared with compound **3** and **6**, it can be observed that the formation of the hydrazide (**3**) decreased the activities, but the formation of carboxylic acid (**6**) increased the activities in A549, MCF-7, and HeLa cell lines (Figure 12).

**Figure 13:**
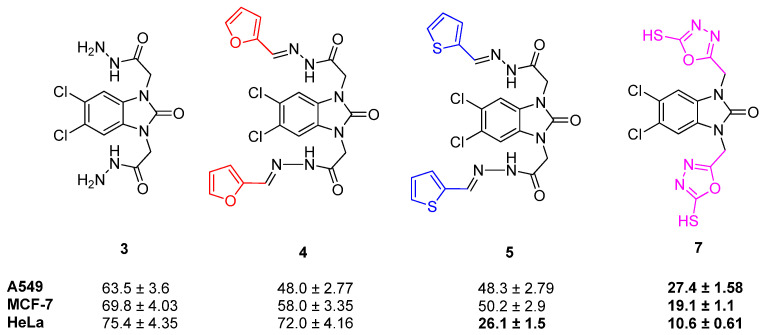
Structure and activity of compounds **3**–**5** and **7**.

When the anticancer activities of compounds **3**, **4**, **5**, and **7** were compared, the formation of Schiff bases and oxadiazole moieties enhanced the activity against all tested cell lines. Among these compounds, the formation of oxadiazole moieties (compound **7**) enhanced the activities to a greater extent against all tested cell lines. Among the Schiff bases (compounds **4** and **5**), the activities against the A549 and MCF-7 cell lines are similar, whereas the main differences can be seen against the HeLa cell line (72.0 ± 4.16 and 26.1 ± 1.5, respectively) (Figure 13).

**Figure 14:**
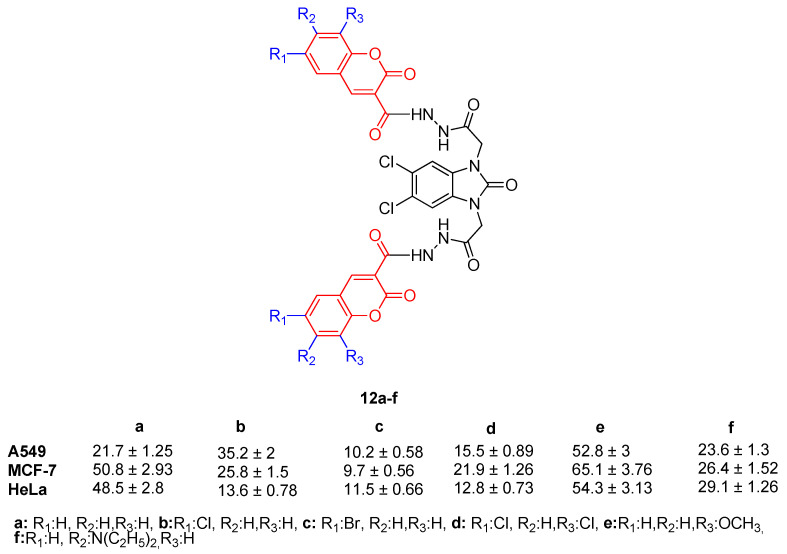
Structure and activity of compounds **12a**–**f**.

Furthermore, when the activities of compounds **12a**–**f** were investigated, the addition of a coumarin cycle to compound **3** resulted in greater anticancer activity against all tested cancer cell lines. In activity results of compounds **12a**–**f**, substitution at the eighth position of the coumarin ring with OCH_3_ decreased the activity against all tested cancer cell lines. When the activity of **12a**–**f** against A549 cell lines was examined, only bromine substitution at the sixth position and two chlorine substitutions at the sixth and eighth positions of the coumarin ring (compounds **12c** and **12d**) have resulted in activity enhancement. When the activities of these compounds against MCF-7 and HeLa cell lines were investigated, the activity enhancement with substitution on the coumarin ring (except OCH_3_ substitution at the eighth position, compound **12e**) can be seen (Figure 14).

## 3. Material and Methods

### 3.1. Chemistry

#### 3.1.1. Synthesis of 5,6-Dichloro-1,3-dihydro-2*H*-benzimidazol-2-one (**1**)

This compound was synthesized from the reaction of 4,5-dichloro-o-phenylenediamine and urea in diglyme according to our previously published protocol [58,69]. The compounds 4,5-dichloro-o-phenylenediamine (1.79 g, 10 mmol) and urea (0.90 g, 15 mmol) were dissolved in diglyme (25 mL) and the mixture was boiled for 4 h under a reflux condenser. The mixture was then cooled to room temperature, the precipitate was filtered off, washed with cold acetone, and dried in a vacuum desiccator.

Yield: 1.93 g (95%), m.p.: 350–352 °C (343–345 °C [70]), IR (KBr, cm^−1^): 1684 (C=O), 3154 (NH). ^1^H-NMR (400 MHz, DMSO-*d*_6_), δ, ppm: 7.07 (2H, s, ArH), 10.89 (2H, s, NH). Elemental analysis: Calculated for C_7_H_4_Cl_2_N_2_O, C, 41.41; H, 1.99; N, 13.80; found: C, 41.36; H, 1.95; N, 13.76.

#### 3.1.2. Synthesis of Diethyl 2,2′-(5,6-Dichloro-2-oxo-1*H*-benzimidazole-1,3(2*H*)-diyl)diacetate (**2**)

Compound 1 (2.03 g, 10 mmol) and K_2_CO_3_ (6.90 g, 50 mmol) were added to dry acetone (50 mL) and stirred at room temperature for 15 min; then ethyl bromoacetate (2.48 mL, 22 mmol) was added and stirred at room temperature overnight. After adding water (100 mL) to the final mixture, the precipitate was filtered off. It was washed with plenty of water, and the resulting product was purified by crystallization in acetone and dried in a desiccator.

Yield: 3.60 g (96%), m.p.: 155–157 °C, IR (KBr, cm^−1^): 1219 (C-O), 1722, 1745 (C=O), 2968 (CH), 3084 (ArH). ^1^H-NMR (400 MHz, DMSO-*d*_6_), δ, ppm: 1.91 (t, *J* = 7.2 Hz, 6H, 2CH_3_), 4.15 (q, *J* = 7.2 Hz, 4H, 2OCH_2_), 4.74 (s, 4H, 2NCH_2_), 7.62 (2H, s, ArH). ^13^C-NMR (100 MHz, DMSO-*d*_6_), δ, ppm: 14.90 (CH_3_), 42.50 (NCH_2_), 61.58 (OCH_2_), 108.93, 121.61, 129.67 (Ar-C), 154.17 (C=O, benzimidazolone), 166.94 (C=O). Elemental analysis: Calculated for C_15_H_16_Cl_2_N_2_O_5_, C, 48.02; H, 4.30; N, 7.47; found: C, 47.96; H, 4.26; N, 7.44.

#### 3.1.3. Synthesis of 2,2′-(5,6-Dichloro-2-oxo-1*H*-benzimidazole-1,3(2*H*)-diyl)diacetohydrazide (**3**)

Compound 2 (3.47 g, 10 mmol) was dissolved in ethanol (50 mL), and hydrazine monohydrate (50 mmol) was added to the mixture; it was then boiled for 3 h under reflux conditions. After the reaction was complete, the mixture was cooled to room temperature, and the precipitate was filtered off. The product was purified by washing with ethanol. It was dried in a vacuum desiccator.

Yield: 2.98 g (86%), m.p.: 357–358 °C, IR (KBr, cm^−1^): 1663, 1728 (C=O), 2932 (CH), 3036 (ArH), 3206, 3302 (NHNH_2_). ^1^H-NMR (400 MHz, DMSO-*d*_6_), δ, ppm: 4.29 + 4.79 (s, 4H, NH_2_), 4.45 (s, 4H, 2NCH_2_), 7.41 (2H, s, ArH), 9.30 + 8.68 (s, 2H, NH). ^13^C-NMR (100 MHz, DMSO-*d*_6_), δ, ppm: 42.62 (NCH_2_), 108.54, 121.45, 129.92 (Ar-C), 153.99 (C=O, benzimidazolone), 166.54 (C=O). Elemental analysis: Calculated for C_11_H_12_Cl_2_N_6_O_3_, C, 38.06; H, 3.48; N, 24.21; found: C, 38.01; H, 3.40; N, 24.14.

#### 3.1.4. Synthesis of Compounds **4** and **5**

Compound 3 (3.47 g, 10 mmol) and DMF (10 mL) were placed in a round-bottom flask and the mixture was stirred for 15 min at room temperature. Then, furfural or thiophen-2-carbaldehyde (2.2 mmol) and 2 drops of acetic acid (as a catalyst) were added to the mixture. The mixture was boiled for 8 h under reflux conditions. The final mixture was brought to room temperature, then taken into cold water to precipitate the products. The precipitated substance was filtered, then purified by washing with ethanol. It was dried on CaCl_2_ in a vacuum desiccator.

**2,2′-(5,6-Dichloro-2-oxo-1*H*-benzimidazole-1,3(2*H*)-diyl)bis[*N*′-(furan-2-ylmethylidene) acetohydrazide] (4):**

Yield: 3.92 g (78%), m.p.: 293–294 °C, IR (KBr, cm^−1^): 1295 (C-O), 1681, 1729 (C=O), 2930 (CH), 3061 (ArH), 3120 (NH). ^1^H-NMR (400 MHz, DMSO-*d*_6_), δ, ppm: 4.66 + 5.00 (s, 4H, NCH_2_, trans and cis amid conformer, *cis*/*trans* ratio 71/29), 7.11 (2H, s, ArH), 7.45 (2H, s, ArH), 7.60–7.65 (m, 4H, ArH), 8.21 + 8.43 (s, 2H, N=CH, E/Z amid conformer, *E*/*Z* ratio 68/32), 11.71 (s, 1H, NH). ^13^C-NMR (100 MHz, DMSO-*d*_6_), δ, ppm: 42.64 + 43.03 (CH_2_), 112.61, 114.18, 114.48, 123.75, 130.24, 134.70, 137.60, 145.57, 149.41 (Ar-C), 153.28 (C=O, benzimidazolone), 168.12 + 163.42 (C=O). Elemental analysis: Calculated for C_21_H_16_Cl_2_N_6_O_5_, C, 50.11; H, 3.20; N, 16.70; found: C, 50.03; H, 3.14; N, 16.62.

**2-(5,6-Dichloro-2-oxo-3-{2-oxo-2-[(2-(thiophen-2-ylmethylidene)hydrazinyl]ethyl}-2,3-dihydro-1*H*-benzimidazol-1-yl)-*N*′-[thiophen-2-ylmethylidene]acetohydrazide (5):**

Yield: 3.63 g (68%), m.p.: 350–351 °C, IR (KBr, cm^−1^): 1677, 1724 (C=O), 2924 (CH), 3063 (ArH), 3125 (NH). ^1^H-NMR (400 MHz, DMSO-*d*_6_), δ, ppm: 4.66 + 5.01 (s, 4H, NCH_2_, trans and cis amid conformer, *cis*/*trans* ratio 71/29), 7.11 (2H, s, ArH), 7.46 (2H, s, ArH), 7.55–7.65 (m, 4H, ArH), 8.21 + 8.42 (s, 2H, N=CH, E/Z amid conformer, *E*/*Z* ratio 70/30), 11.73 (s, 1H, NH). ^13^C-NMR (100 MHz, DMSO-*d*_6_), δ, ppm: 42.05 + 43.06 (CH_2_), 110.78, 123.77, 128.28, 128.37, 129.15, 130.23, 131.19, 139.02, 139.72, 142.95 (Ar-C), 154.28 (C=O, benzimidazolone), 163.23 + 167.90 (C=O). Elemental analysis: Calculated for C_21_H_16_Cl_2_N_6_O_3_S_2_, C, 47.11; H, 3.01; N, 15.70; found: C, 47.04; H, 2.92; N, 15.64.

#### 3.1.5. Synthesis of 2,2′-(5,6-Dichloro-2-oxo-1H-benzo[d]imidazole-1,3(2H)-diyl)diacetic Acid (**6**)

Compound 2 (3.47 g, 10 mmol) was dissolved in ethanol (20 mL) and NaOH solution (4N, 20 mL) was added to the mixture. Then, the mixture was refluxed for 5 h. After the completion of the reaction, the mixture was cooled and poured into cold water. It was neutralized with 1 N HCl. The precipitated product was filtered off, washed with water and recrystallized from ethanol to obtain the pure product.

Yield: 2.84 g (90%), m.p.: 290–291 °C, IR (KBr, cm^−1^): 1205 (C-O) 1722, 1749 (C=O), 2983 (CH), 3075 (ArH), 3125 (NH), 2500–3300 (COOH). ^1^H-NMR (400 MHz, DMSO-*d*_6_), δ, ppm: 4.63 (s, 4H, NCH_2_), 7.59 (2H, s, ArH), 13.16 (s, 2H, COOH). ^13^C-NMR (100 MHz, DMSO-*d*_6_), δ, ppm: ^13^C-NMR (100 MHz, DMSO-*d*_6_), δ, ppm: 42.65 (CH_2_), 112.62, 123.65, 130.24 (Ar-C), 149.50 (C=O, benzimidazolone), 168.11 (C=O carboxylic acid). Elemental analysis: Calculated for C_11_H_8_Cl_2_N_2_O_5_, C, 41.40; H, 2.53; N, 8.78; found: C, 41.37; H, 2.47; N, 8.70.

#### 3.1.6. Synthesis of 5,6-Dichloro-1,3-bis((5-mercapto-1,3,4-oxadiazol-2-yl)methyl)-1*H*-benzo[d]imidazol-2(3*H*)-one (**7**)

Compound 3 (3.47 g, 10 mmol) was dissolved in ethanol (25 mL) and stirred for 5 min at room temperature. 2N KOH solution (25 mL) was added to the solution and it was stirred for 5 min at room temperature again. Then, CS_2_ (22 mmol) was added to the solution, and it was refluxed for 5 h. After the completion of the reaction, the mixture was cooled and poured into cold water. It was neutralized with 1 N HCl. The precipitated product was filtered off, washed with water, and recrystallized from ethanol to obtain the pure product.

Yield: 3.75 g (87%), m.p.: 243–244 °C, IR (KBr, cm^−1^): 1687 (C=O), 2925 (CH), 3061 (ArH), 3125 (NH). ^1^H-NMR (400 MHz, DMSO-*d*_6_), δ, ppm: 5.23 (s, 4H, NCH_2_), 7.69 (2H, s, ArH). ^13^C-NMR (100 MHz, DMSO-*d*_6_), δ, ppm: 42.11 (CH_2_), 114.48, 123.73, 130.24 (Ar-C), 149.12 (C=O, benzimidazolone), 154.28, 163.42 (C=N), 177.11 (C=S oxadiazole). Elemental analysis: Calculated for C_13_H_8_Cl_2_N_6_O_3_S_2_, C, 36.20; H, 1.87; N, 19.49; found: C, 36.16; H, 1.83; N, 19.42.

#### 3.1.7. Synthesis of 5,6-Dichloro-1,3-bis(2-oxo-2-phenylethyl)-1,3-dihydro-2*H*-benzimidazol-2-one (**8**)

Compound 1 (2.03 g, 10 mmol) and dry K_2_CO_3_ (8.30 g, 60 mmol) were added to DMF (30 mL) and stirred at room temperature for 15 min, then 2-bromo-1-phenylethanone (4.38 g, 22 mmol) was added and stirred at room temperature overnight. After adding water (100 mL) to the final mixture, the precipitate was filtered off. It was washed with copious water, and the resulting product was purified by washing with hot acetone and dried in a desiccator.

Yield: 3.91 g (89%), m.p.: 259–260 °C, IR (KBr, cm^−1^): 1227 (C-O), 1684, 1736 (C=O), 2977 (CH), 3059 (ArH). ^1^H-NMR (400 MHz, DMSO-*d*_6_), δ, ppm: 5.57 (s, 4H, NCH_2_), 7.59–7.73 (m, 8H, ArH), 8.09 (s, 4H, ArH). ^13^C-NMR (100 MHz, DMSO-*d*_6_), δ, ppm: 48.28 (CH_2_), 110.64, 123.77, 128.61, 128.67, 129.33, 130.21, 134.54, 134.73 (Ar-C), 154.28 (2C=N), 155.38 (C=O, benzimidazolone), 163.47 (C=O). Elemental analysis: Calculated for C_23_H_16_Cl_2_N_2_O_3_, C, 62.88; H, 3.67; N, 6.38; found: C, 62.81; H, 3.62; N, 6.32.

#### 3.1.8. Synthesis of 5,6-Dichloro-1,3-bis((4-(4-chlorophenyl)piperazin-1-yl)methyl)-1*H*-benzo[d]imidazol-2(3*H*)-one (**9**)

4-Chlorophenyl piperazine (22 mmol), DMF (10 mL) and formaldehyde (37%, 2.5 mL) were placed in a reaction balloon and stirred for 10 min at room temperature. Then, compound 1 (2.03 g, 10 mmol) was added and the mixture was stirred overnight at room temperature. The mixture was poured into cold water, and the product was precipitated. It was filtered off, washed with water and dried. It was recrystallized from ethyl acetate to obtain a pure product.

Yield: 3.91 g (89%), m.p.: 233–234 °C, IR (KBr, cm^−1^): 1699 (C=O), 2982 (CH), 3015 (ArH). ^1^H-NMR (400 MHz, DMSO-*d*_6_), δ, ppm: 2. 68 (s, 8H, CH_2_), 3.07 (s, 8H, CH_2_), 4.70 (s, 4H, NCH_2_), 6.85 (m, 4H, ArH), 7.16 (m, 4H, ArH), 7.64 (s, 2H, ArH). ^13^C-NMR (100 MHz, DMSO-*d*_6_), δ, ppm: 48.39, 50.14 (CH_2_), 62.73 (NCH_2_), 110.93, 111.23, 122.81, 123.84, 128.97, 130.33, 150.15 (Ar-C), 155.40 (C=O, benzimidazolone). Elemental analysis: Calculated for C_29_H_30_Cl_4_N_6_O, C, 56.14; H, 4.87; N, 13.55; found: C, 56.04; H, 4.80; N, 13.47.

#### 3.1.9. Synthesis of Compounds **10a**–**f**

A mixture of corresponding salicyl aldehyde derivative (10 mmol) and 2,2-dimethyl-1,3-dioxane-4,6-dione (Meldrum’s acid) (1.58 g, 11 mmol) in ethanol (50 mL, containing 0.5 mL of pyridine) was refluxed in a round-bottom flask for 6 h. After completion of the reaction (monitored by TLC, ethyl acetate/hexane, 4:1), the solvent was evaporated under reduced pressure. The residue was washed with H_2_O and recrystallized from ethanol/water (3:2).

2**-Oxo-2*H*-chromene-3-carboxylic acid (10a)**

Yield: 1.39 g (73%), m.p.: 189–190 °C (189–190 °C [71]). Elemental analysis: Calculated for C_10_H_6_O_4_, C, 63.16; H, 3.18; found: C, 63.13; H, 3.15.

**6-Chloro-2-oxo-2*H*-chromene-3-carboxylic acid (10b)**

Yield: 2.20 g (63%), m.p.: 194–195 °C (194–195 °C [71]). Elemental analysis: Calculated for C_10_H_5_ClO_4_, C, 53.48; H, 2.24; found: C, 53.40; H, 2.19.

**6-Bromo-2-oxo-2*H*-chromene-3-carboxylic acid (10c)**

Yield: 1.80 g (67%). Mp 197–198 °C (195–196 °C [71]). Elemental analysis: Calculated for C_10_H_5_BrO_4_, C, 44.64; H, 1.87.; found: C, 44.60; H, 1.82.

**6,8-Dichloro-2-oxo-2*H*-chromene-3-carboxylic acid (10d)**

Yield: 2.34 g (65%), m.p.: 223–224 °C (222–223 °C [71]). Elemental analysis: Calculated for C_10_H_4_Cl_2_O_4_, C, 46.37; H, 1.56; found: C, 46.31; H, 1.52.

**8-Methoxy-2-oxo-2*H*-chromene-3-carboxylic acid (10e)**

Yield: 1.95 g (75%). mp 217–218 °C (214–215 °C [71]). Elemental analysis: Calculated for C_11_H_8_O_5_, C, 60.00; H, 3.66; found: C, 59.94; H, 3.61.

**7-Diethylamino-2-oxo-2*H*-chromene-3-carboxylic acid (10f)**

Yield: 2.46 g (68%), m.p.: 235–236 °C (232–233 °C [71]). Elemental analysis: Calculated for C_14_H_15_NO_4_, C, 64.36; H, 5.79, N,5.36; found: C, 64.31; H, 5.75, N,5.31.

#### 3.1.10. Synthesis of Compounds **11a**–**f**

Compounds **10a**–**f** (10 mmol) were dissolved in dichloromethane (75 mL), and thionyl chloride (1.78 g, 15 mmol) was added. The mixture was stirred for 30 min at room temperature, and 1H-benzotriazole (5.95 g, 50 mmol) was added. Then, the mixture was stirred overnight at room temperature. The precipitated salt was filtered off, and the filtrate was washed with 10% Na_2_CO_3_ solution (50 mL) and 1 N HCl (50 mL). Then, the solvent was removed under reduced pressure, and the product was obtained as a white solid. It was recrystallized from CH_2_Cl_2_/hexane, 1/1.

**3-(1*H*-Benzotriazol-1-ylcarbonyl)-2*H*-chromen-2-one (11a)**

Yield: 2.12 g (73%), m.p.: 180–181 °C (179–180 °C [71]). Elemental analysis: Calculated for C_19_H_9_N_3_O_3_, C, 65.98; H, 3.11; N, 14.43; found: C, 63.13; H, 3.07; N, 14.37.

**3-(1*H*-Benzotriazol-1-ylcarbonyl)-6-chloro-2*H*-chromen-2-one (11b)**

Yield: 2.20 g (63%), m.p.: 248–249 °C (248–249 °C [71]). ^1^H-NMR (400 MHz, DMSO-*d*_6_), δ, ppm: 8.74 (s, 1H, H-4); 8.29–8.05 (m, 2H, Ar-H); 8.05 (d, 1H, *J *= 2.4 Hz, Ar-H); 7.98–7.79 (m,2H, Ar-H); 7.68–7.64 (m,1H, Ar-H); 7.64 (d, 1H, *J* = 8.0 Hz, Ar-H). ^13^C-NMR (100 MHz, DMSO-*d*_6_), δ, ppm: 162.8 (C=O); 157.3 (C=O coumarin C-2); 153.2 (coumarin C-3); 146.9 (coumarin C-4); 145.9; 134.4; 131.7; 130.9; 129.5; 127.5; 122.6; 120.7; 119.3; 119.1; 114.2 (Ar-C). Elemental analysis: Calculated for C_16_H_8_ClN_3_O_3_, C, 59.00; H, 2.48; N, 12.90; found: C, 58.96; H, 2.45; N, 12.85.

**3-(1*H*-Benzotriazol-1-ylcarbonyl)-6-bromo-2*H*-chromen-2-one (11c)**

Yield: 2.52 g (68%), m.p.: 250–251 °C (250–251 °C [71]). ^1^H-NMR (400 MHz, DMSO-*d*_6_), δ, ppm: 7.55 (d, 1H, *J* = 8.4, Ar H), 7.70 (t, 1H, *J* = 8.8, Ar H), 7.86 (t, 1H, *J* = 8.8, Ar H), 7.96 (dd, 1H, *J* = 8.8, *J* = 2.4, Ar H), 8.21 (d, 1H, *J* = 2.0, Ar H), 8.31–8.28 (m, 2H, Ar H), 8.74 (s, 1H, H-4). ^13^C-NMR (100 MHz, DMSO-*d*_6_), δ, ppm: 114.20, 117.30, 119.40, 119.90, 120.80, 122.60, 127.60, 131.00, 131.90, 132.50, 137.30, 145.90, 146.93, 153.68, 157.29 (C=O), 162.79 (C=O). Elemental analysis: Calculated for C_16_H_7_BrN_3_O_3_, C, 51.92; H, 2.18; N, 11.35; found: C, 51.85; H, 2.14; N, 11.31.

**3-(1*H*-Benzotriazol-1-ylcarbonyl)-6,8-dichloro-2*H*-chromen-2-one (11d)**

Yield: 2.34 g (65%), m.p.: 263–264 °C (263–264 °C [71]). ^1^H-NMR (400 MHz, DMSO-*d*_6_), δ, ppm: 8.76 + 8.67 (s, 1H, coumarin C-4), 8.29 (s, 1H, coumarin H-7), 8.27 (s, 1H, coumarin H-5), 8.11–7.99 (m, 2H, Ar-H), 7.85 (t, 1H, *J* = 7.2 Hz, Ar-H), 7.67 (t, 1H, *J* = 7.2 Hz, Ar-H). ^13^C-NMR (100 MHz, DMSO-*d*_6_), δ, ppm: 163.9 (C=O), 158.1 (C=O coumarin C-2), 152.4 (coumarin C-3), 148.7 (coumarin C-4), 146.8, 138.4, 133.1, 132.4, 129.9, 128.4, 123.1, 121.9, 116.0, 114.1, 112.0 (Ar-C). Elemental analysis: Calculated for C_16_H_7_Cl_2_N_3_O_3_, C, 53.36; H, 1.96; N, 11.67; found: C, 53.33; H, 1.92; N, 11.61.

**3-(1*H*-Benzotriazol-1-ylcarbonyl)-8-methoxy-2*H*-chromen-2-one (11e)**

Yield: 2.46 g (68%). m.p.: 236–237 °C (236–237 °C [71]). ^1^H-NMR (400 MHz, DMSO-*d*_6_), δ, ppm: 8.78 + 8.66 (s, 1H, coumarin C-4), 8.30 (d, 1H, *J* = 7.6 Hz, coumarin H-7), 7.84 (m, 2H, Ar-H), 7.66 (m, 1H, Ar-H), 7.57 (m, 3H, Ar-H), 3.94 (s, 3H, OCH_3_). ^13^C-NMR (100 MHz, DMSO-*d*_6_), δ, ppm: 163.9 (C=O), 158.1 (C=O coumarin C-2), 152.4 (coumarin C-3), 148.7 (coumarin C-4), 146.8, 138.4, 133.1, 132.4, 129.9, 128.4, 123.1, 121.9, 116.0, 114.1, 112.0 (Ar-C). Elemental analysis: Calculated for C_17_H_11_N_3_O_4_, C, 63.55; H, 3.45; N, 13.08; found: C, 63.51; H, 3.40; N, 13.06.

**3-(1*H*-Benzotriazol-1-ylcarbonyl)-7-diethylamino-2*H*-chromen-2-on (11f)**

Yield: 2.46 g (68%), m.p: 212–213 °C (210–211 °C [71]). Elemental analysis: Calculated for C_20_H_18_N_4_O_3_, C, 66.29; H, 5.01; N, 15.46; found: C, 66.22; H, 4.97; N, 15.42.

#### 3.1.11. Synthesis of Compounds **12a**–**f**

Compound **3** (3.47 g, 10 mmol) was dissolved in DMSO (5 mL) and the mixture was stirred for 10 min at room temperature. Then, the corresponding compound **11a**–**f** (22 mmol) was added. Then the mixture was heated at 80 °C for 7 h under reflux conditions. The final mixture was poured onto the ice-water mixture and the precipitated substance was filtered off and washed with water and hot ethanol for purification.

***N′,N′′′*** **-(2,2′-(5,6-dichloro-2-oxo-1*H*-benzo[d]imidazole-1,3(2*H*)-diyl)bis(acetyl))bis(2-oxo-2H-chromene-3-carbohydrazide) (12a):**

Yield: 4.97 g (72%), m.p.: 343–344 °C, IR (KBr, cm^−1^): 1228 (C-O), 1686–1721 (C=O), 2930 (CH), 3044 (ArH), 3228 (NH). ^1^H-NMR (400 MHz, DMSO-*d*_6_), δ, ppm: 4.72 (s, 4H, NCH_2_), 7.43–7.53 (m, 6H, ArH), 7.58 (m, 2H, ArH), 7.99 (s, 2H, ArH), 8.87 (s, 2H, ArH, coumarin C_3_H), 10.65 (s, 2H, NH), 11.17 (s, 1H, NH). ^13^C-NMR (100 MHz, DMSO-*d*_6_), δ, ppm: 42.57 (NCH_2_), 109.98, 116.62, 116.86, 118.45, 118.47, 118.70, 123.79, 125.67, 129.90, 130.80, 134.87, 148.40 (Ar-C), 154.37, 159.29, 160.22, 164.54 (C=O). Elemental analysis: Calculated for C_31_H_20_Cl_2_N_6_O_9_, C, 53.85, H, 2.92, N, 12.15; found: C, 53.76, H, 2.86, N, 12.08.

***N′,N′′′*** **-(2,2′-(5,6-dichloro-2-oxo-1*H*-benzo[d]imidazole-1,3(2*H*)-diyl)bis(acetyl))bis(6-chloro-2-oxo-2H-chromene-3-carbohydrazide) (12b):**

Yield: 5.62 g (74%), m.p.: 345–346 °C, IR (KBr, cm^−1^): 1196 (C-O), 1705 (C=O), 2935 (CH), 3064 (ArH), 3216 (NH). ^1^H-NMR (400 MHz, DMSO-*d*_6_), δ, ppm: 4.85 (s, 4H, NCH_2_), 7.54 (d, 2H, *J* = 8.8 Hz, ArH), 7.78 (m, 2H, ArH), 8.11 (s, 2H, ArH), 8.32 (s, 2H, ArH), 8.83 (s, 2H, ArH, coumarin C_3_H), 10.64 (s, 2H, NH), 11.24 (s, 1H, NH). ^13^C-NMR (100 MHz, DMSO-*d*_6_), δ, ppm: 41.85 (NCH_2_), 109.98, 116.62, 116.86, 118.45, 118.47, 118.70, 123.79, 125.67, 129.90, 130.80, 134.87, 148.40 (Ar-C), 154.159.29, 160.22, 164.54 (C=O). Elemental analysis: Calculated for C_31_H_18_Cl_4_N_6_O_9_, C, 48.97, H, 2.39, N, 11.05; found: C, 48.92, H, 2.33, N, 10.96.

***N′,N′′′*** **-(2,2′-(5,6-dichloro-2-oxo-1*H*-benzo[d]imidazole-1,3(2*H*)-diyl)bis(acetyl))bis(6-bromo-2-oxo-2H-chromene-3-carbohydrazide) (12c):**

Yield: 5.62 g (74%), m.p.: 342–343 °C, IR (KBr, cm^−1^): 1224 (C-O), 1713 (C=O), 2976 (CH), 3016 (ArH), 3205 (NH). ^1^H-NMR (400 MHz, DMSO-*d*_6_), δ, ppm: 4.71 (s, 4H, NCH_2_), 7.45–7.55 (m, 4H, ArH), 7.88 (d, 2H, *J* = 8.0 Hz, ArH), 8.24 (s, 2H, ArH), 8.81 (s, 2H, ArH, coumarin C_3_H), 10.62 (s, 2H, NH), 11.23 (s, 1H, NH). ^13^C-NMR (100 MHz, DMSO-*d*_6_), δ, ppm: 44.57 (NCH_2_), 116.47, 116.15, 118.47, 125.27, 126.90, 127.22, 130.25, 138.92, 148.55 (Ar-C), 152.60 (C=O), 153.73 (coumarin C_3_), 160.22, 164.54 (C=O). Elemental analysis: Calculated for C_31_H_18_Br_2_Cl_2_N_6_O_9_, C, 43.84, H, 2.14, N, 9.90; found: C, 43.75, H, 2.08, N, 9.82.

***N′,N′′′*** **-(2,2′-(5,6-dichloro-2-oxo-1*H*-benzo[d]imidazole-1,3(2*H*)-diyl)bis(acetyl))bis(6,8-Dichloro-2-oxo-2H-chromene-3-carbohydrazide) (12d):**

Yield: 5.62 g (74%), m.p.: 316–317 °C, IR (KBr, cm^−1^): 1217 (C-O), 1719 (C=O), 2948 (CH), 3068 (ArH), 3241 (NH). ^1^H-NMR (400 MHz, DMSO-*d*_6_), δ, ppm: 4.71 (s, 4H, NCH_2_), 7.51 (s, 2H, ArH), 8.10 (s, 4H, ArH), 8.80 (s, 2H, ArH, coumarin C_3_H), 10.57 (s, 2H, NH), 11.14 (s, 1H, NH). ^13^C-NMR (100 MHz, DMSO-*d*_6_), δ, ppm: 45.05 (NCH_2_), 116.65, 118.71, 120.08, 126.70, 126.81, 130.78, 134.86, 134.97, 147.54, 148.48 (Ar-C), 154.34, 155.73, 160.14, 161.42 (C=O). Elemental analysis: Calculated for C_31_H_16_Cl_4_N_6_O_9_, C, 44.90, H, 1.94, N, 10.13; found: C, 44.81, H, 1.86, N, 10.10.

***N′,N′′′*** **-(2,2′-(5,6-dichloro-2-oxo-1*H*-benzo[d]imidazole-1,3(2*H*)-diyl)bis(acetyl))bis(8-methoxy-2-oxo-2H-chromene-3-carbohydrazide) (12e):**

Yield: 5.93 g (79%), m.p.: 346–347 °C, IR (KBr, cm^−1^): 1194, 1279 (C-O), 1695, 1713 (C=O), 2987 (CH), 3056 (ArH), 3242 (NH). ^1^H-NMR (400 MHz, DMSO-*d*_6_), δ, ppm: 3.92 (s, 6H, OCH_3_), 4.71 (s, 4H, NCH_2_), 7.35–7.55 (m, 8H, ArH), 8.84 (s, 2H, ArH, coumarin C_3_H), 10.65 (s, 2H, NH), 11.16 (s, 2H, NH). ^13^C-NMR (100 MHz, DMSO-*d*_6_), δ, ppm: 44.58 (NCH_2_), 56.67 (OCH_3_), 110.57, 116.86, 118.43, 119.11, 119.29, 125.64, 126.92, 127.22, 136.07, 138.90, 146.74, 148.80 (Ar-C), 153.73, 159.16, 159.97, 164.60 (C=O). Elemental analysis: Calculated for C_33_H_24_Cl_2_N_6_O_11_, C, 52.74, H, 3.22, N, 11.18; found: C, 52.65, H, 3.18, N, 11.12.

***N′,N′′′*** **-(2,2′-(5,6-dichloro-2-oxo-1*H*-benzo[d]imidazole-1,3(2*H*)-diyl)bis(acetyl))bis(8-methoxy-2-oxo-2H-chromene-3-carbohydrazide) (12f):**

Yield: 5.93 g (79%), m.p.: 311–312 °C, IR (KBr, cm^−1^): 1187, 1263 (C-O), 1695, 1704 (C=O), 2930 (CH), 3016 (ArH), 3237 (NH). ^1^H-NMR (400 MHz, DMSO-*d*_6_), δ, ppm: 1.11 (m, 12H, CH_3_), 3.32 (m, 8H, 4NCH_2_, CH_3_), 4.69 (s, 4H, NCH_2_), 6.59 (s, 2H, ArH), 6.80 (s, 2H, ArH), 7.51 (s, 2H, ArH), 7.69 (s, 2H, ArH), 8.67 (s, 2H, ArH, coumarin C_3_H), 10.53 (s, 2H, NH), 11.08 (s, 2H, NH). ^13^C-NMR (100 MHz, DMSO-*d*_6_), δ, ppm: 12.57 (CH_3_), 28.08 (NCH_2_CH_3_), 44.85 (NCH_2_), 108.12, 110.82, 119.04, 122.14, 122.57, 125.74, 126.93, 127.22, 132.30, 138.30, 148.80 (Ar-C), 153.29, 157.86, 160.30, 161.78 (C=O). Elemental analysis: Calculated for C_39_H_38_Cl_2_N_8_O_9_, C, 56.19, H, 4.59, N, 13.44; found: C, 56.11, H, 4.53, N, 13.36.

### 3.2. Anticancer Activity

#### 3.2.1. Cell Viability

Cell viability assay was performed on the human lung cancer (A549) (ATCC CCL-185), human breast cancer (MCF-7) (ATCC-HTB-22), cervical cancer (HeLa) (ATCC-CCL-2), and human embryonic kidney 293 (HEK293) (ATCC-CRL-1573) cell lines, which were purchased from Deutsche Sammlung von Mikroorganismen und Zellkulturen (DSMZ, Braunschweig, Germany). The cells were maintained in Dulbecco’s modified Eagle’s medium (DMEM), supplemented with 10% fetal bovine serum (FBS), 2 mM L-glutamine, 100 U/mL penicillin, and 100 mg/L streptomycin (Biochrom AG, Berlin, Germany) at 37 °C in a humidified atmosphere of 5% CO_2_.

The cytotoxic effect was determined by the MTT (3-(4,5-dimethyl-2-thiazolyl)-2,5-diphenyl-2H-tetrazolium bromide) cell viability assay. Cells were seeded at an initial concentration of 1 × 10^5^ cells/mL in 96-well microplates. Cells were then treated with different concentrations (0.5, 5, 10, 25, 50, 100 and 200 µM) of the novel synthesized compounds for 48 h. After the incubation period, the formed formazan crystals were dissolved in dimethyl sulfoxide (DMSO), and the optical density (OD) of the compounds was measured at 570 nm using a spectrophotometer (BMG Labtech, Ortenberg, Germany). Cytotoxicity was expressed as an increase in the mean percentage of cytotoxicity relative to the unexposed control ± standard deviation (SD). Doxorubicin (0.5, 5 and 50 µM) was used as a positive control. Control values were set at 0% cytotoxicity. IC50 was calculated by fitting the data to a sigmoidal curve using a four-parameter logistic model and was presented as an average of three independent measurements. The IC_50_ value was reported at a 95% confidence interval and the calculation was performed using GraphPad Prism software 10.0.0 (San Diego, CA, USA). The values of the blank wells were subtracted from each well of treated and control cells, and the inhibition of growth by 50% was calculated compared to untreated controls. Doxorubicin was used as a positive control. Each sample was tested in triplicate.

#### 3.2.2. Selectivity Index (SI) Analysis

To evaluate the selectivity of the tested compound(s) toward cancer cells over normal cells, the Selectivity Index (SI) was calculated using the following formula:

SI = IC_50_ (normal cells)/IC_50_ (cancer cells)

An SI value greater than 2 was considered to indicate selective cytotoxicity, while values below 2 were interpreted as non-selective. Higher SI values reflect greater therapeutic potential due to reduced toxicity toward normal cells [72].

### 3.3. Biochemical Analyses

#### 3.3.1. LDH Assay

LDH leakage assay was determined using the LDH Assay Kit (Cat no. ab102526, Abcam, Cambridge, UK) on the culture medium of a new set (at a density of 1 × 10^5^ cells/well) of A549, HeLa, and MCF-7 cells exposed to calculated IC50 values of the compounds for 48 h. One hundred microliters of culture medium was transferred to a new 96-well plate. One hundred microliters of LDH reaction solution was added to each well, and absorbance was measured at 490 nm using an ELISA plate reader (BMG Labtech, Ortenberg, Germany) after 30 min.

#### 3.3.2. TOS Levels

TOS were evaluated using a commercially available kit (Rel Assay Diagnostics^®^, Gaziantep, Turkey) according to the manufacturer’s instructions. A549, HeLa, and MCF-7 cells were exposed to calculated IC50 values of the compounds for 48 h. The TOS assay quantifies oxidizing agents based on their ability to convert an iron (II) ion chelator complex to iron (III) ions. The results were calibrated against hydrogen peroxide (H_2_O_2_, 250 µM) as a positive control for 48 h and expressed as µmol H_2_O_2_ equivalents per liter. The analysis was performed in three independent biological replicates (n = 3).

#### 3.3.3. Determination of Intracellular GSH/GSSG Ratio

Intracellular reduced (GSH) and oxidized (GSSG) glutathione levels were measured using the GSH/GSSG Ratio Detection Assay Kit (Cayman Chemical, Ann Arbor, MI, USA, Cat. No: 703002) according to the manufacturer’s protocol. Briefly, A549, HeLa, and MCF-7 cells were seeded in 6-well plates at a density of 1 × 10^5^ cells/well and treated with the IC_50_ concentrations of test compounds for 48 h. After treatment, cells were washed with cold PBS and lysed with MES buffer provided in the kit.

To prevent oxidation of GSH to GSSG during processing, samples were deproteinized using the supplied metaphosphoric acid solution and neutralized accordingly. Total glutathione and GSSG levels were measured separately by adding the appropriate buffers and reagents to the wells of a 96-well plate, followed by the addition of DTNB recycling reagent and glutathione reductase. Absorbance was read at 405 nm using a microplate reader (BMG Labtech, Germany). The amount of GSH was calculated by subtracting 2 × GSSG from the total glutathione, and the GSH/GSSG ratio was used to assess intracellular redox status.

### 3.4. Cell Cycle Analysis

Cell cycle distribution was assessed by propidium iodide (PI) staining and flow cytometry. HeLa, MCF-7, and A549 cells were seeded in 6-well plates at a density of 1 × 10^5^ cells per well and allowed to attach overnight. Cells were then treated with IC50 concentrations of compounds for 48 h. Following exposure, both adherent and floating cells were collected, washed twice with cold phosphate-buffered saline (PBS, pH 7.4), and fixed overnight in 70% cold ethanol at 4 °C. After fixation, the cells were centrifuged and resuspended in 500 µL of PI/RNase staining solution containing 50 µg/mL PI and 100 µg/mL RNase A (Sigma-Aldrich^®^, St. Louis, MO, USA), followed by incubation in the dark for 30 min at 37 °C. The stained samples were analyzed using a Muse™ Cell Analyzer (Merck, Millipore, Germany) equipped with a 488 nm excitation laser and a 617 nm emission filter. A minimum of 10,000 events per sample were acquired after exclusion of debris and doublets via FSC-A vs. FSC-H gating. The DNA content histograms were generated automatically, and cell populations were expressed as percentages of G0/G1, S, and G2/M phases using the Muse™ Cell Cycle Analysis Software 1.5.0.0. All assays were conducted in triplicate biological repeats (n = 3), and results were reported as mean ± SEM.

### 3.5. Molecular Docking Protocol

An in silico molecular modeling study was conducted using the Schrödinger Molecular Modeling Package (Schrödinger Version 2018-2: Maestro) to examine the binding affinities and interaction profiles of the synthesized compounds against two distinct protein targets, namely Vascular Endothelial Growth Factor Receptor 2 (VEGFR2) and Cyclin-Dependent Kinase 4 (CDK4) [73]. The structures of these targets were downloaded from the RCSB Protein Data Bank (PDB) under accession codes 4ASD and 7SJ3, respectively [74,75]. The 4ASD entry representing the juxtamembrane and kinase domains of VEGFR2 complexed with Sorafenib and the 7SJ3 entry corresponding to the active CDK4-Cyclin D3 complex bound to the inhibitor abemaciclib were prepared for docking using the Protein Preparation Wizard module. This preparation included removing co-crystallized solvent molecules (except those within 5 Å of the binding site), assigning atomic charges and force field parameters (using the OPLS4 force field), and correcting the protonation states for ionizable residues via the Epik submodule according to predicted pKa values at physiological pH, ensuring the biologically relevant tautomeric and protonation states of the active site residues. The 3D structures of the synthesized compounds were processed via the LigPrep module to generate all possible stereoisomers and low-energy conformers, and the appropriate protonation and tautomeric states were assigned for each molecule to obtain energetically favorable structures. Molecular docking simulations were performed using the Induced Fit Docking (IFD) algorithm combined with the Extra Precision (XP) mode to maximize accuracy in pose estimation and scoring [76].

The predictive power of molecular docking methodology was established through a redocking validation procedure. We utilized the co-crystallized inhibitors, Sorafenib for the VEGFR-2 active site and Abemaciclib for the CDK4 pocket, to test the accuracy of the protocol. The obtained RMSD values of 0.965 Å (VEGFR-2) and 0.783 Å (CDK4) confirm the high precision of the method. Since both values are significantly below the generally accepted 2.0 Å threshold, the validity of the selected docking method is affirmed, ensuring the protocol is reliable [77].

### 3.6. Statistical Analysis

Statistical analysis was performed using SPSS 20.0 (SPSS, Chicago, IL, USA). Differences between groups were assessed using one-way analysis of variance (ANOVA), followed by the Tukey post-hoc test for multiple comparisons. All experiments were performed in triplicate, and data were expressed as the means ± standard deviation. A *p*-value less than 0.05 (≤0.05) was considered statistically significant.

## 4. Conclusions

In conclusion, we successfully synthesized a new series of benzimidazolone-bridged hybrid compounds containing thiophene, furan, oxadiazole, piperazine, and coumarin moieties, and assessed their cytotoxic activity against three cancer cell lines: lung cancer (A549), breast cancer (MCF-7), and cervical cancer (HeLa). Among the synthesized compounds, the piperazine-containing derivative (compound **9**) showed the best activities with IC_50_ values of 9.6 ± 0.55 µM and 8.5 ± 0.49 µM against lung cancer (A549) and cervical cancer (HeLa) cell lines, respectively. Compound 1**2c** (containing the coumarin moiety) showed the best activity with an IC50 value of 9.7 ± 0.56 µM against the MCF-7 cell line. All the synthesized compounds showed no toxicity against non-cancerous cells (HEK-293), and most of them showed a higher selectivity index compared to doxorubicin against the tested cancer cell lines. Based on the structure–activity relationship studies, it is concluded from the results that the synthesis of carboxylic acid (**6**) and phenyl ethenone (**8**) derivatives of compound **1** has resulted in increased anticancer activities against the tested cell lines; however, synthesis of ester (**2**) and hydrazide (**3**) derivatives of compound **1** has resulted in decreased anticancer activities when compared to the activities of compound **1**. Synthesis of Schiff bases (**4** and **5**), oxadiazole (**7**), and coumarin (**12a**–**f**) derivatives from compound **3** resulted in increased activity against all tested cancerous cell lines. Most of the synthesized compounds showed a higher selectivity index value than the standard drug doxorubicin.

Following the cell viability screening, a dual-criterion strategy was applied to select compounds for mechanistic evaluation. Only those molecules that (i) demonstrated selective cytotoxicity (SI > 2 in at least one tumor cell line) and (ii) showed strong molecular docking affinity toward VEGFR2 and/or CDK4 were advanced to redox-related assays. Based on this integrated approach, compounds **5**, **6**, **7**, **12b**, **12c**, **12d**, and **1** were identified as the most promising candidates. Among these, compounds **6**, **7**, **12c**, and **12d** showed the most consistent activity across multiple cell lines and exhibited potent binding energies (VEGFR2: −10.5 to −13.4 kcal/mol; CDK4: −6.5 to −8.6 kcal/mol). In particular, compound **7** displayed strong dual-target affinity and high selectivity toward HeLa cells; compound 12c provided the best VEGFR2 interaction within the whole series; compound **12d** demonstrated HeLa- and A549-specific selectivity; and compound **6** exerted balanced cytotoxicity against A549, MCF-7, and HeLa.

These selected compounds were subsequently subjected to oxidative stress–related biochemical analysis. The most active and selective derivatives induced a significant increase in LDH release, elevated total oxidant status (TOS), and a marked depletion of intracellular GSH/GSSG balance, indicating oxidative stress–mediated loss of membrane integrity rather than nonspecific toxicity. Complementary cell-cycle analysis revealed a pronounced, cell line–dependent disturbance of cell-cycle progression, including a clear G_2_/M arrest in HeLa cells for several of the lead compounds, in line with inhibition of CDK4–Cyclin D–regulated checkpoints and proliferative signaling.

Molecular docking study-based binding analyses of the key active compounds, including **6**, **7**, **9**, **12c**, and **12d**, across VEGFR2 and CDK4–Cyclin D3 revealed their capacity to engage critical hinge, DFG, and hydrophobic regions via a combination of hydrogen bonding, π–π stacking, and halogen interactions, supporting their potential as ATP-competitive inhibitors with favorable binding orientations and selectivity toward kinase active sites. The strong agreement between docking predictions, redox imbalance, LDH leakage, and G_2_/M cell-cycle arrest provides a coherent mechanistic framework for the antiproliferative effects observed in vitro.

Taken together, the integration of structure–activity relationships, cytotoxicity and selectivity profiles, redox-related biochemical markers, cell-cycle modulation, and in silico target engagement highlights compounds **6**, **7**, **9**, **12c**, and **12d** as the most compelling benzimidazolone-based lead structures. These multitarget candidates merit further optimization and in-depth preclinical evaluation as promising anticancer agents.

## Figures and Tables

**Table 1:** IC_50_ (µM) values and Selectivity Indices (SIs) of the synthesized compounds in human cancer cells and non-cancerous cells.

Compound	A549 IC_50_ Values (µM ± SD)/SI	MCF-7 IC_50_ Values (µM ± SD)/SI	HeLa IC_50_ Values (µM ± SD)/SI	HEK 293 IC_50_ Values (µM ± SD)
**1**	29.5 ± 1.7/2.03	41.3 ± 2.4/1.46	31.6 ± 1.82/1.9	60.1 ± 3.47
**2**	60.8 ± 3.51/1.17	63.2 ± 3.65/1.13	68.8 ± 3.9/1.04	71.3 ± 4.12
**3**	63.5 ± 3.6/1.06	69.8 ± 4.03/0.97	75.4 ± 4.35/0.89	67.9 ± 3.9
**4**	48.0 ± 2.77/1.15	58.0 ± 3.35/0.95	72.0 ± 4.16/0.76	55.0 ± 3.17
**5**	48.3 ± 2.79/1.22	50.2 ± 2.9/1.18	26.1 ± 1.5/2.27	59.4 ± 3.43
**6**	30.6 ± 1.76/2.04	28.3 ± 1.63/2.21	31.2 ± 1.8/2	62.7 ± 3.62
**7**	27.4 ± 1.58/1.4	19.1 ± 1.1/2.01	10.6 ± 0.61/3.63	38.5 ± 2.2
**8**	19.1 ± 1.1/0.91	21.7 ± 1.25/0.8	23.9 ± 1.38/0.73	17.5 ± 1.01
**9**	9.6 ± 0.55/1.58	11.2 ± 0.46/1.35	8.5 ± 0.49/1.78	15.2 ± 0.87
**12a**	21.7 ± 1.25/2.05	50.8 ± 2.93/0.87	48.5 ± 2.8/0.91	44.6 ± 2.57
**12b**	35.2 ± 2/1.39	25.8 ± 1.5/1.9	13.6 ± 0.78/3.61	49.1 ± 2.83
**12c**	10.2 ± 0.58/2.01	9.7 ± 0.56/2.21	11.5 ± 0.66/1.86	21.5 ± 1.24
**12d**	15.5 ± 0.89/2.01	21.9 ± 1.26/1.42	12.8 ± 0.73/2.4	31.2 ± 1.8
**12e**	52.8 ± 3/1.14	65.1 ± 3.76/0.92	54.3 ± 3.13/1.1	60.2 ± 3.47
**12f**	23.6 ± 1.3/1.84	26.4 ± 1.52/1.64	29.1 ± 1.26/1.49	43.5 ± 2.51
**Doxorubicin**	4.3 ± 0.2/1.20	6.4 ± 0.37/0.77	3.4 ± 0.19/1.54	5.2 ± 0.3

IC_50_ values are presented as mean ± SD (n = 3). Selectivity Index (SI) values were calculated using HEK-293 as the reference normal cell line.

**Table 2:** Effects of selected compounds on LDH release, total oxidative status (TOS), and GSH/GSSG ratio in HeLa, A549, and MCF-7 cells.

Cell Line	Group	LDH (µU/mL)	TOS (µmol H_2_O_2_ eq/L)	GSH/GSSG (nmol/mg Protein)
HeLa	Control	6.2 ± 0.8	2.1 ± 0.3	31.2 ± 2.4
	H_2_O_2_	41.5 ± 3.2 ^a^	10.4 ± 0.7 ^a^	6.8 ± 0.9 ^a^
	5	24.8 ± 2.1 ^a,b^	6.9 ± 0.5 ^a,b^	11.8 ± 1.3 ^a,b^
	6	27.3 ± 2.5 ^a,b^	7.8 ± 0.6 ^a,b^	10.6 ± 1.1 ^a,b^
	7	30.9 ± 2.7 ^a,b^	8.6 ± 0.7 ^a,b^	9.4 ± 1.0 ^a,b^
	12b	28.5 ± 2.3 ^a,b^	8.1 ± 0.6 ^a,b^	10.1 ± 1.1 ^a,b^
	12d	29.7 ± 2.4 ^a,b^	8.4 ± 0.6 ^a,b^	9.7 ± 1.0 ^a,b^
A549	Control	5.9 ± 0.6	2.0 ± 0.2	30.4 ± 2.1
	H_2_O_2_	40.7 ± 3.0 ^a^	10.5 ± 0.8 ^a^	7.1 ± 0.9 ^a^
	1	19.8 ± 1.7 ^a,b^	6.1 ± 0.4 ^a,b^	13.5 ± 1.3 ^a,b^
	6	24.5 ± 2.0 ^a,b^	7.3 ± 0.5 ^a,b^	11.2 ± 1.0 ^a,b^
	12c	30.4 ± 2.6 ^a,b^	8.5 ± 0.7 ^a,b^	9.5 ± 0.8 ^a,b^
	12d	27.3 ± 2.2 ^a,b^	7.9 ± 0.6 ^a,b^	10.1 ± 0.9 ^a,b^
MCF-7	Control	5.4 ± 0.6	1.9 ± 0.2	29.8 ± 2.3
	H_2_O_2_	39.5 ± 2.7 ^a^	10.1 ± 0.7 ^a^	7.4 ± 0.9 ^a^
	6	22.7 ± 1.8 ^a,b^	6.9 ± 0.5 ^a,b^	12.1 ± 1.2 ^a,b^
	7	25.3 ± 2.0 ^a,b^	7.6 ± 0.6 ^a,b^	11.3 ± 1.0 ^a,b^
	12c	28.8 ± 2.2 ^a,b^	8.3 ± 0.6 ^a,b^	10.2 ± 1.0 ^a,b^

Values are presented as mean ± SD (n = 3). H_2_O_2_ was used as a positive oxidative stress control. ^a^ *p* < 0.05 compared to the control group; ^b^ *p* < 0.05 compared to the H_2_O_2_ group.

**Table 3:** Docking scores of all compounds at the active sites of CDK4 and VEGFR2 kinase receptors (kcal/mol).

Compound	VEGFR2	CDK4	Compound	VEGFR2	CDK4
**1**	−9.34	−5.73	**12a**	−10.05	−6.96
**2**	−6.32	−6.31	**12b**	−8.63	−8.62
**3**	−11.44	−9.55	**12c**	−13.44	−6.96
**4**	−11.59	−8.70	**12d**	−11.84	−6.53
**5**	−12.93	−8.01	**12e**	−8.63	−4.10
**6**	−12.17	−7.62	**12f**	−11.37	−6.72
**7**	−10.50	−8.63	Sorafenib	−14.66	-
**8**	−9.88	−7.33	Abemaciclib	-	−13.60
**9**	−12.92	−10.02			

**Table 4:** The predicted ADMET properties of compounds.

Compound	QPlogHERG	QPPCaco	QPlogBB	QPlogKhsa	HOA%
**1**	−3.60	610.14	−0.16	−0.33	87.54
**2**	−4.63	758.73	−0.65	−0.09	100.00
**3**	−4.28	17.69	−2.19	−0.63	35.22
**4**	−5.23	583.52	−0.89	0.22	74.24
**5**	−6.27	656.37	−0.83	0.62	82.95
**6**	−0.30	4.62	−1.34	−0.67	50.78
**7**	−4.96	369.76	−0.57	−0.06	92.11
**8**	−6.97	1556.63	−0.37	0.40	100.00
**9**	−8.33	291.27	1.36	1.26	83.64
**12a**	−8.37	7.39	−3.87	0.31	38.34
**12b**	−8.15	5.33	−3.94	0.54	40.92
**12c**	−8.18	5.34	−3.94	0.59	41.86
**12d**	−7.99	5.33	−3.79	0.80	34.23
**12e**	−8.05	4.92	−4.44	0.25	34.87
**12f**	−8.26	9.28	−4.63	0.98	40.21

## Data Availability

Data are contained within the article and Supplementary Materials.

## Supplementary Materials

The following supporting information can be downloaded at: https://www.mdpi.com/article/10.3390/ph18121899/s1, Figure S1. IR spectra of compound **1**; Figure S2. ^1^H NMR spectra of compound **1**; Figure S3. IR spectra of compound **2**; Figure S4. ^1^H NMR spectra of compound **2**; Figure S5. ^13^C NMR spectra of compound **2**; Figure S6. IR spectra of compound **2**; Figure S7. ^1^H NMR spectra of compound **3**; Figure S8. ^13^C NMR spectra of compound **3**; Figure S9. IR spectra of compound **4**; Figure S10. ^1^H NMR spectra of compound **4**; Figure S11. ^13^C NMR spectra of compound **4**; Figure S12. IR spectra of compound **5**; Figure S13. ^1^H NMR spectra of compound **5**; Figure S14. ^13^C NMR spectra of compound **5**; Figure S15. IR spectra of compound **6**; Figure S16. ^1^H NMR spectra of compound **6**; Figure S17. ^13^C NMR spectra of compound **6**; Figure S18. IR spectra of compound **7**; Figure S19. ^1^H NMR spectra of compound **7**; Figure S20. ^13^C NMR spectra of compound **7**; Figure S21. IR spectra of compound **8**; Figure S22. ^1^H NMR spectra of compound **8**; Figure S23. ^13^C NMR spectra of compound **8**; Figure S24. IR spectra of compound **9**; Figure S25. ^1^H NMR spectra of compound **9**; Figure S26. ^13^C NMR spectra of compound **9**; Figure S27. ^1^H NMR spectra of compound **11b**; Figure S28. ^13^C NMR spectra of compound **11b**; Figure S29. ^1^H NMR spectra of compound **11c**; Figure S30. ^13^C NMR spectra of compound **11c**; Figure S31. ^1^H NMR spectra of compound **11d**; Figure S32. ^13^C NMR spectra of compound **11d**; Figure S33. ^1^H NMR spectra of compound **11e**; Figure S34. ^13^C NMR spectra of compound **11e**; Figure S35. IR spectra of compound **12a**; Figure S36. ^1^H NMR spectra of compound **12a**; Figure S37. ^13^C NMR spectra of compound **12a**; Figure S38. IR spectra of compound **12b**; Figure S39. ^1^H NMR spectra of compound **12b**; Figure S40. ^13^C NMR spectra of compound **12b**; Figure S41. IR spectra of compound **12c**; Figure S42. ^1^H NMR spectra of compound **12c**; Figure S43. ^13^C NMR spectra of compound **12c**; Figure S44. IR spectra of compound **12d**; Figure S45. ^1^H NMR spectra of compound **12d**; Figure S46. ^13^C NMR spectra of compound **12d**; Figure S47. IR spectra of compound **12e**; Figure S48. ^1^H NMR spectra of compound **12e**; Figure S49. ^13^C NMR spectra of compound **12e**; Figure S50. ^13^C NMR spectra of compound **12f**; Figure S51. ^1^H NMR spectra of compound **12f**; Figure S52. ^13^C NMR spectra of compound **12f**.
