# Supplementary material for: Evaluation of Antiproliferative Activity and Molecular Modeling Studies of Some Novel Benzimidazolone-Bridged Hybrid Compounds
Source: Pharmaceuticals (Basel). 2025 Dec 17;18(12):1899. doi: 10.3390/ph18121899 (PMC12735921; doi:10.3390/ph18121899)
Supplement: Supplementary file 1 [file pharmaceuticals-18-01899-s001.zip › pharmaceuticals-4020762-supplementary.pdf]

Evaluation of Antiproliferative Activity and Molecular Modeling Studies of Some Novel Benzimidazolone-Bridged Hybrid Compounds

Okan Güven [1], Emre Menteşe [1], Fatih Yılmaz [2,*], Adem Güner [3], Mustafa Emirik [1] and Nedime Çalışkan [1]

[1] Department of Chemistry, Faculty of Art and Sciences, Recep Tayyip Erdogan University,
53100 Rize, Turkey; okan_guven172@erdogan.edu.tr (O.G.); emre.mentese@erdogan.edu.tr (E.M.); mustafa.emirik@erdogan.edu.tr (M.E.); nedime_caliskan19@erdogan.edu.tr (N.Ç.)

[2] Vocational School of Technical Sciences, Department of Chemistry and Chemical Process Technology,
Recep Tayyip Erdogan University, 53100 Rize, Turkey

[3] Department of Occupational Health and Safety, Faculty of Health Sciences, Sinop University,
57000 Sinop, Turkey; ademguner@sinop.edu.tr

* Correspondence: fyilmaz@erdogan.edu.tr

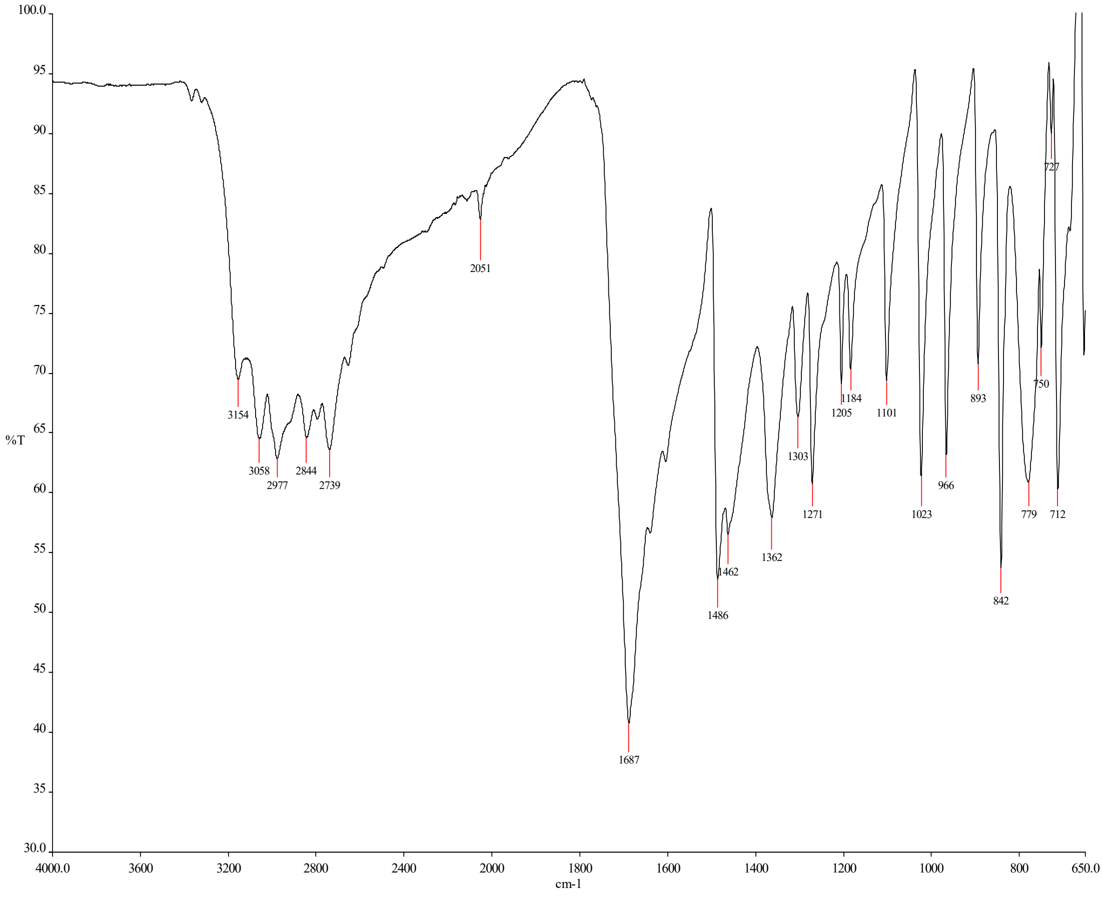

Figure S1. IR spectra of compound **1**

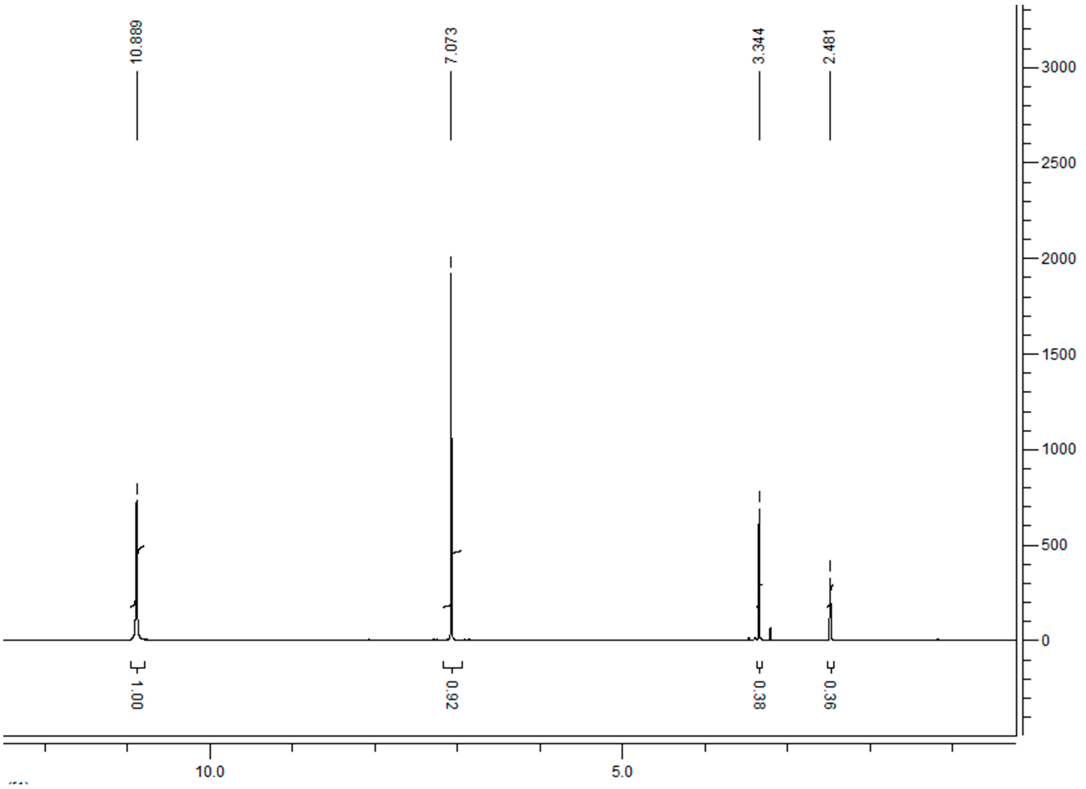

Figure S2. ^{1}H NMR spectra of compound **1**

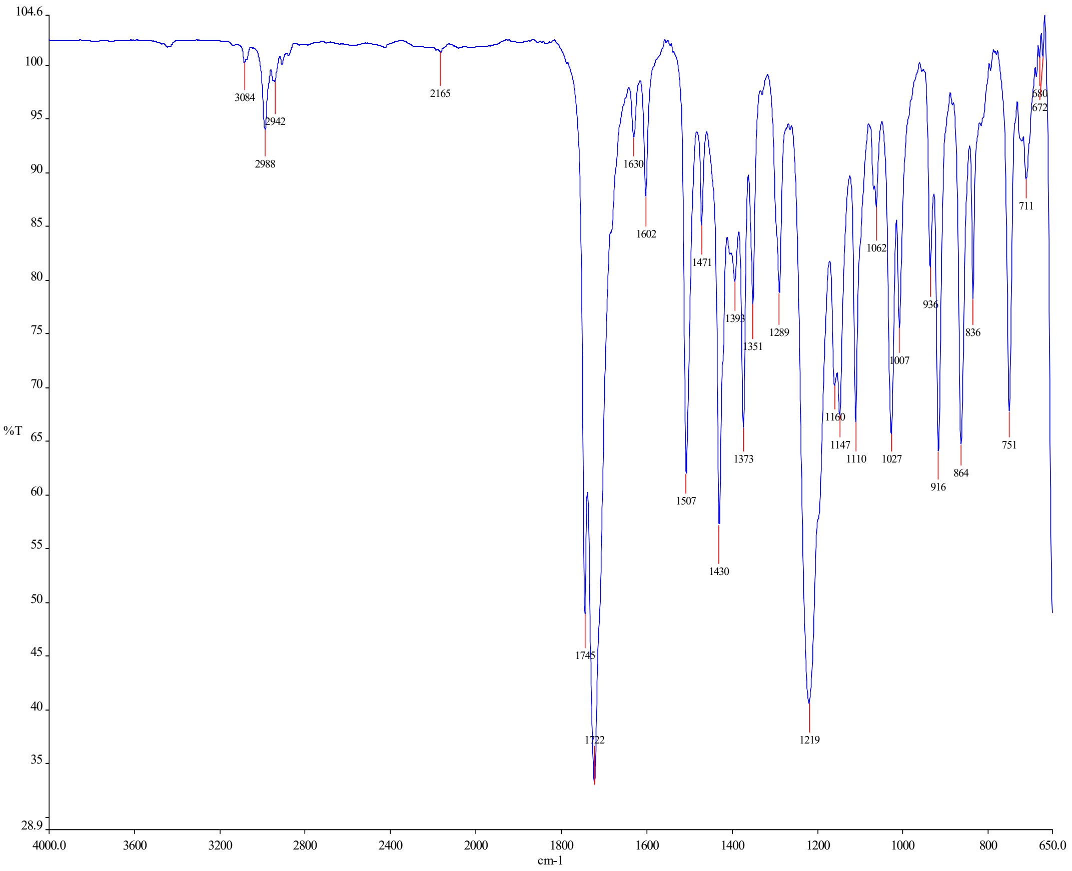

Figure S3. IR spectra of compound **2**

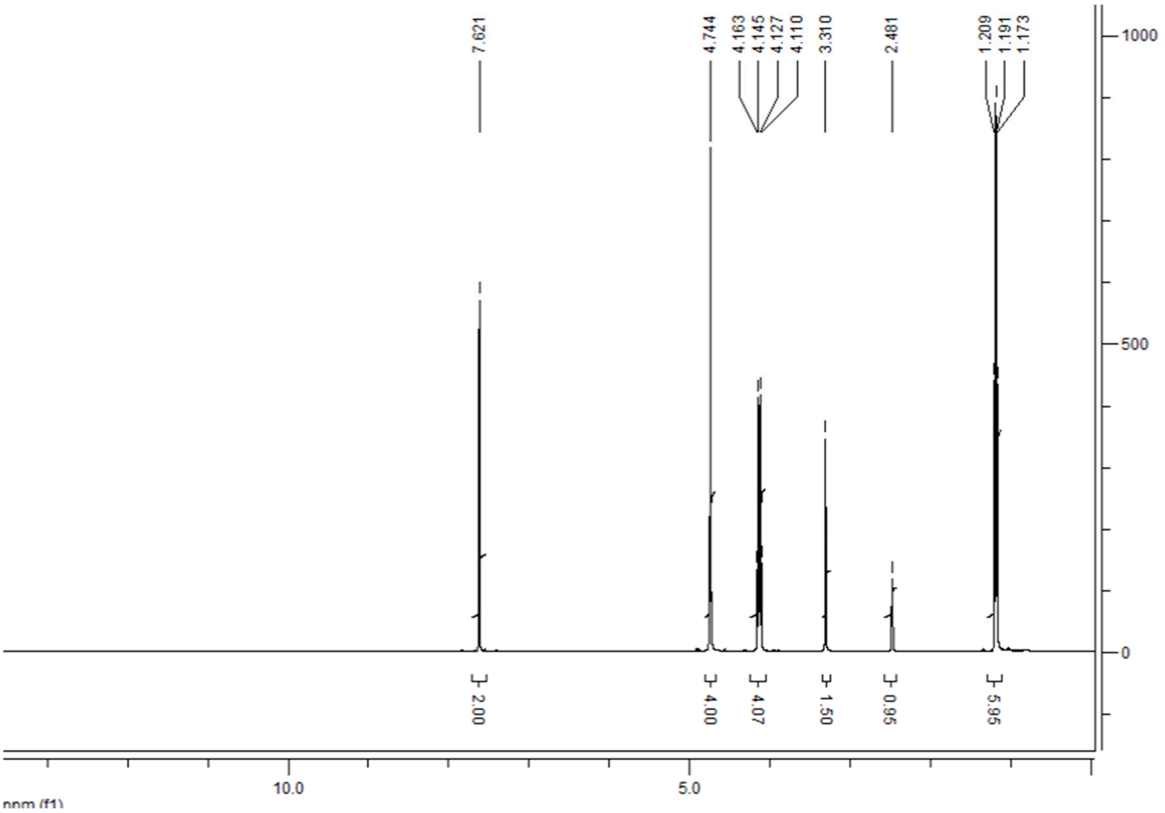

Figure S4. ^{1}H NMR spectra of compound **2**

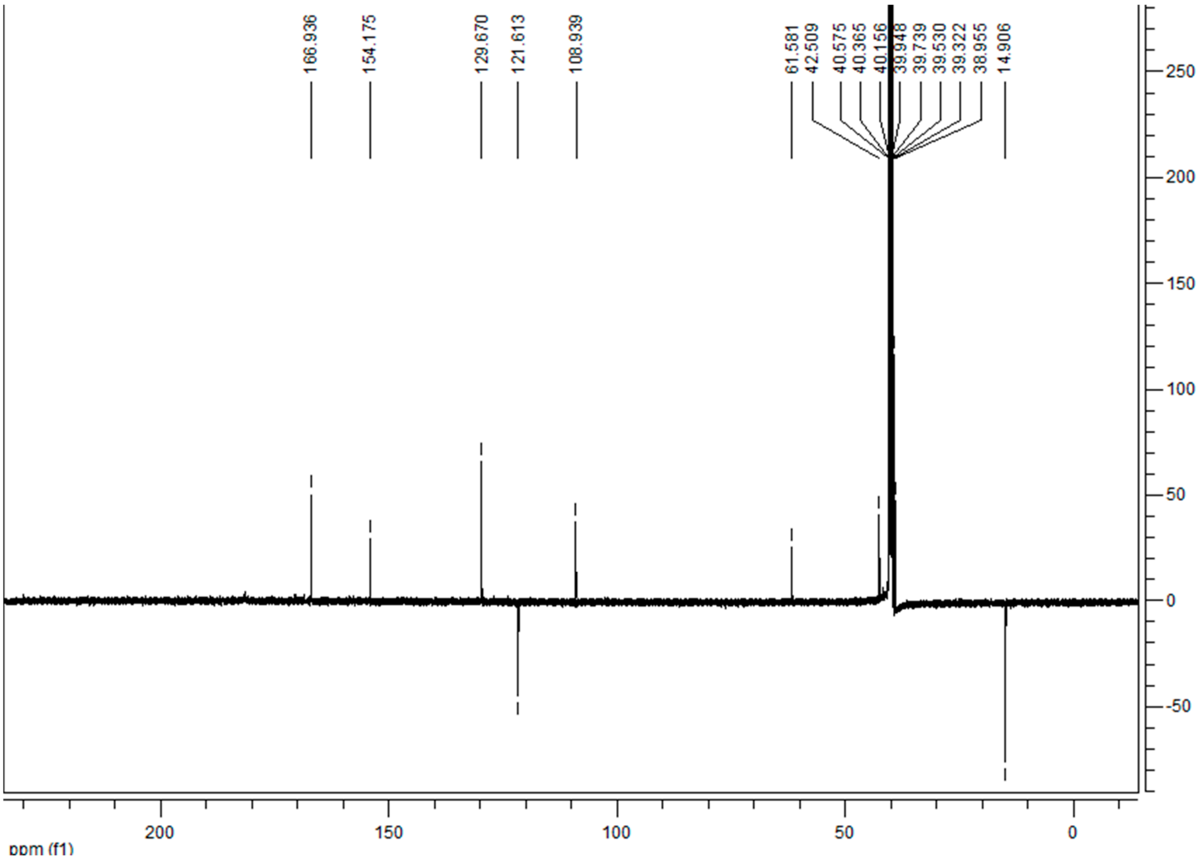

Figure S5. ^{13}C NMR spectra of compound **2**

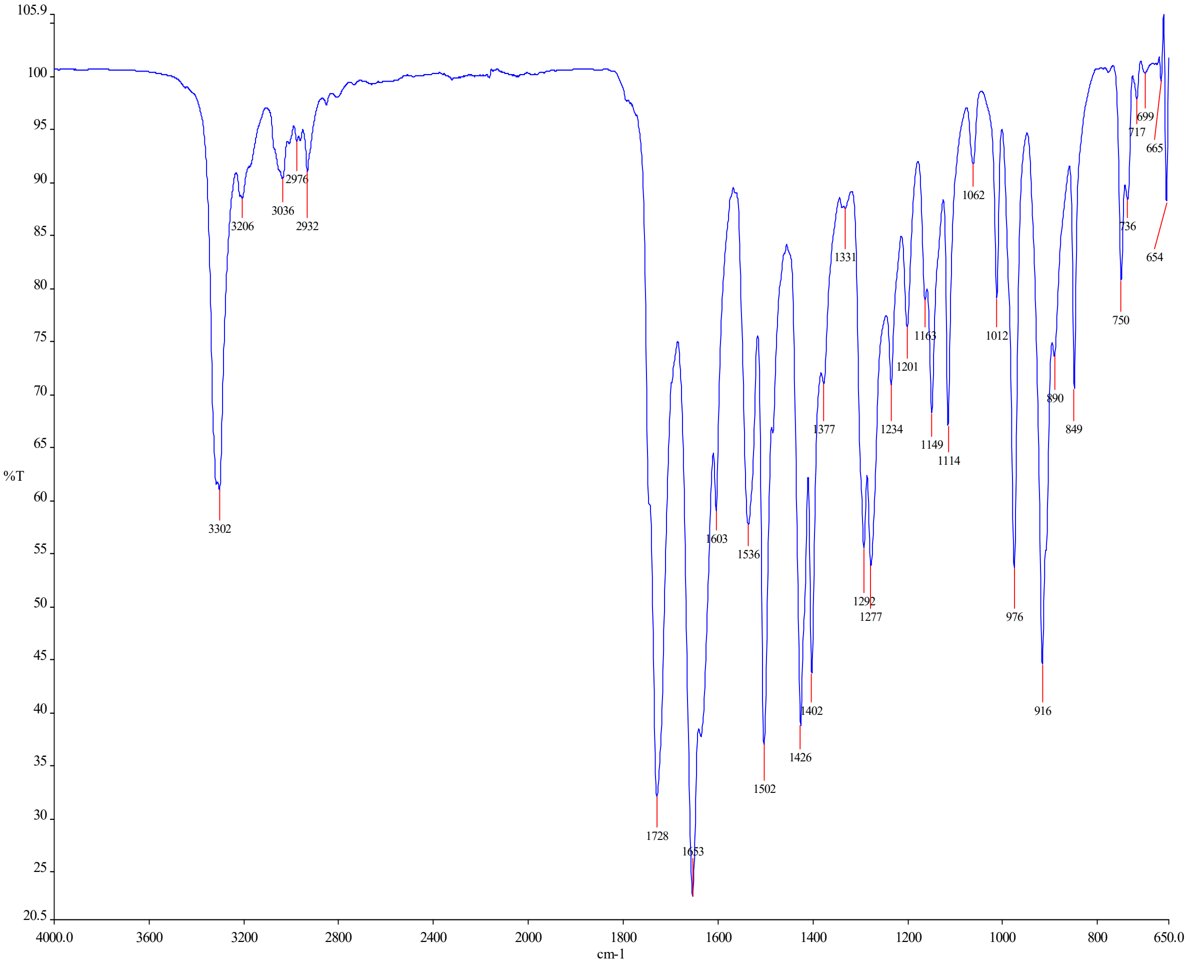

Figure S6. IR spectra of compound **2**

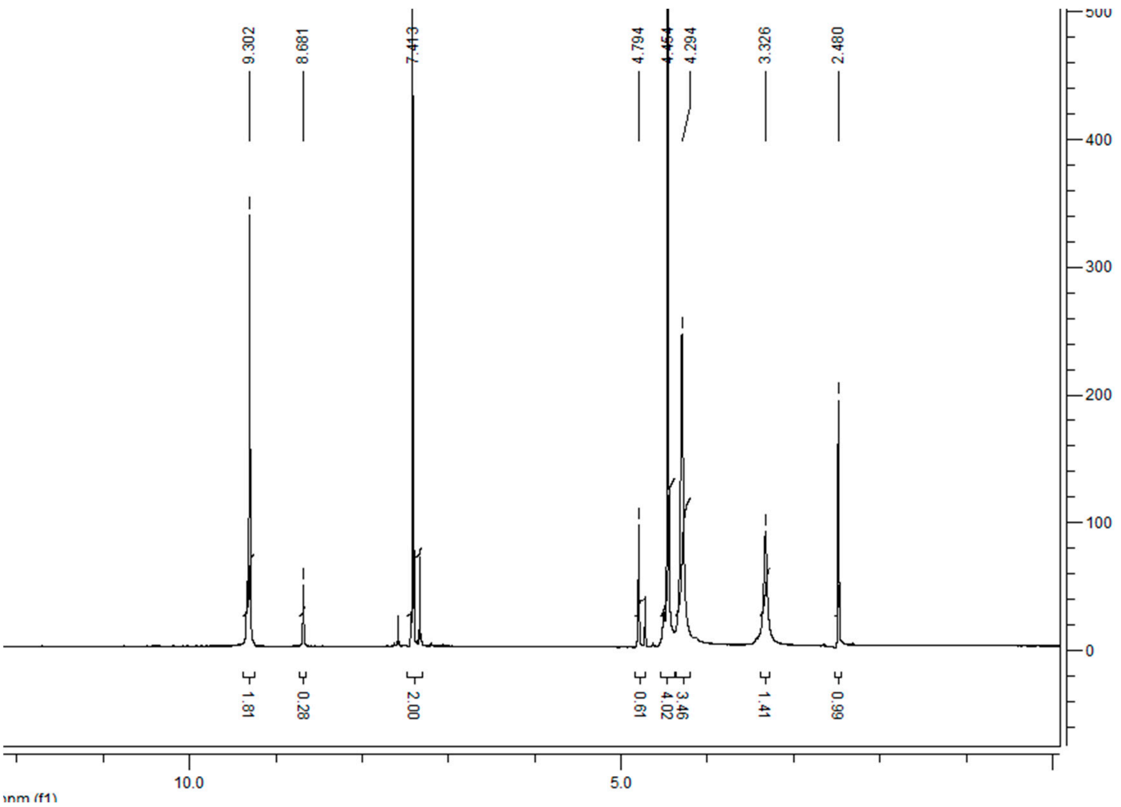

Figure S7. ^{1}H NMR spectra of compound **3**

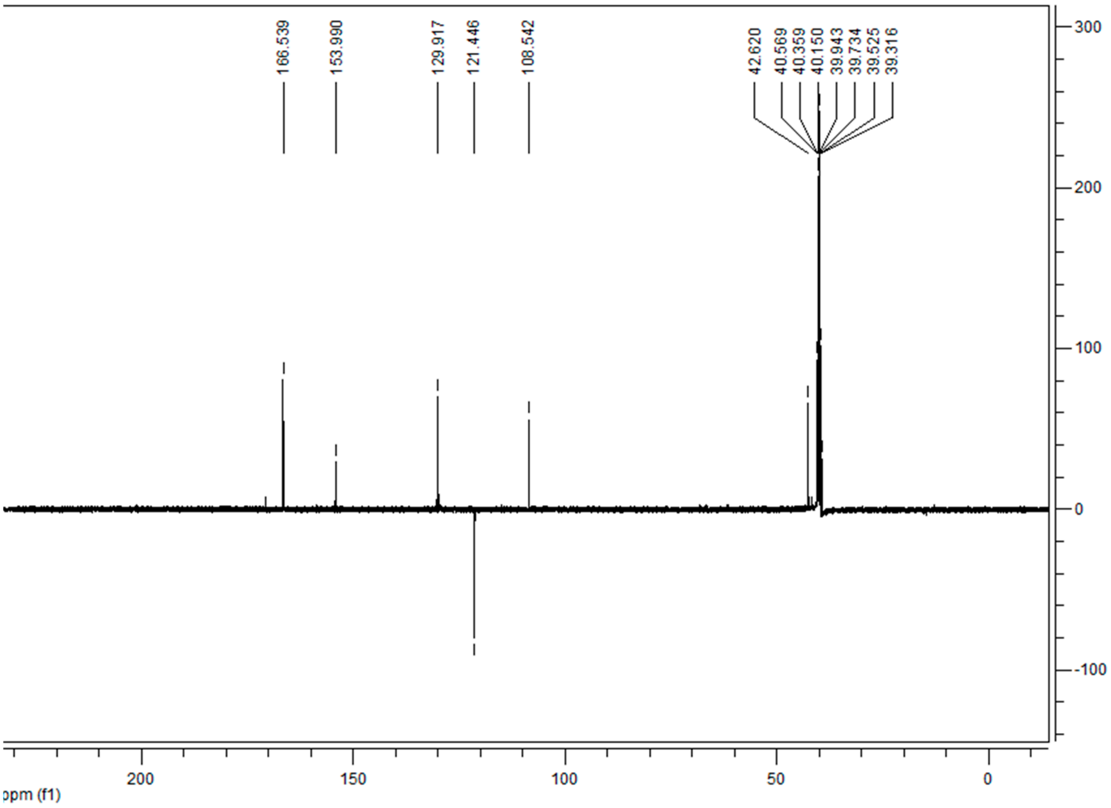

Figure S8. ^{13}C NMR spectra of compound **3**

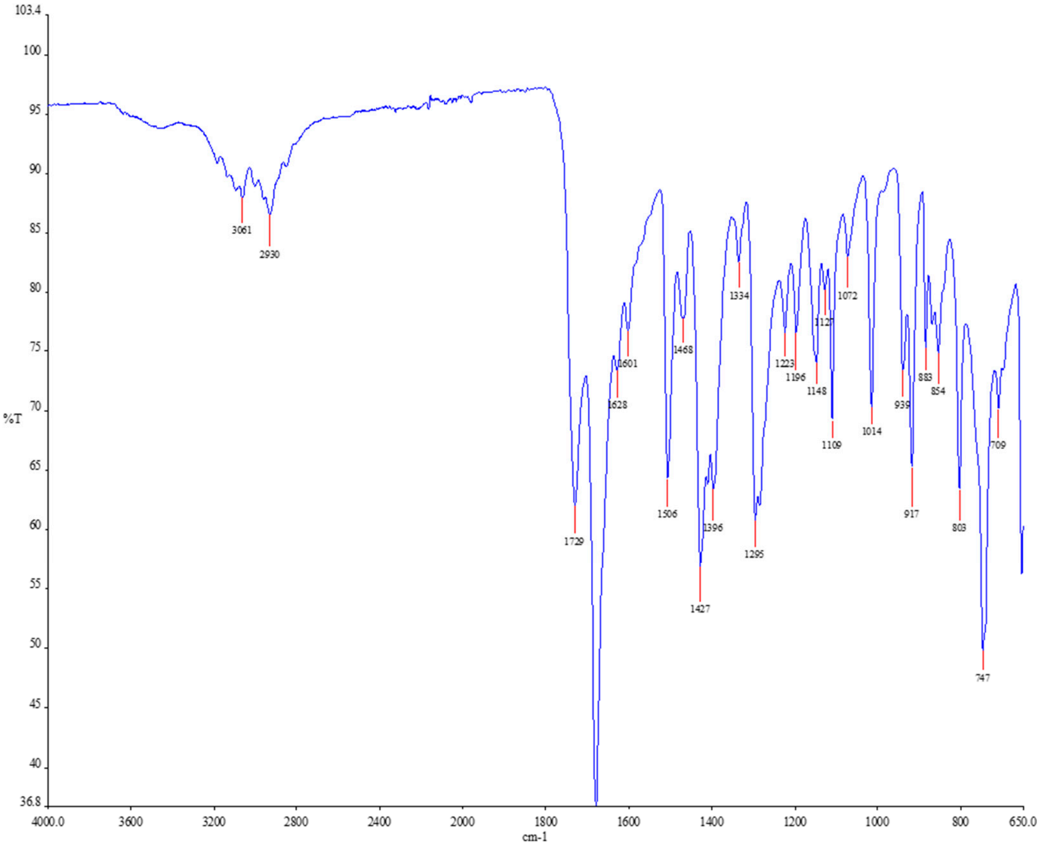

Figure S9. IR spectra of compound **4**

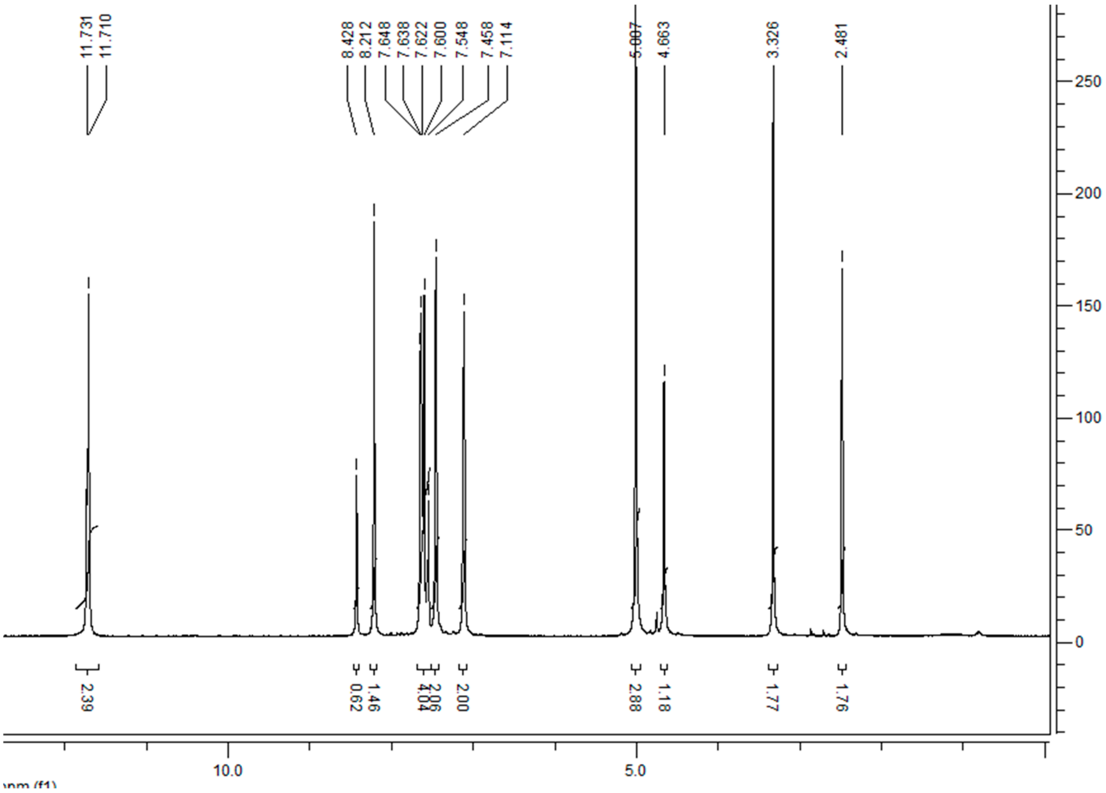

Figure S10. ^{1}H NMR spectra of compound **4**

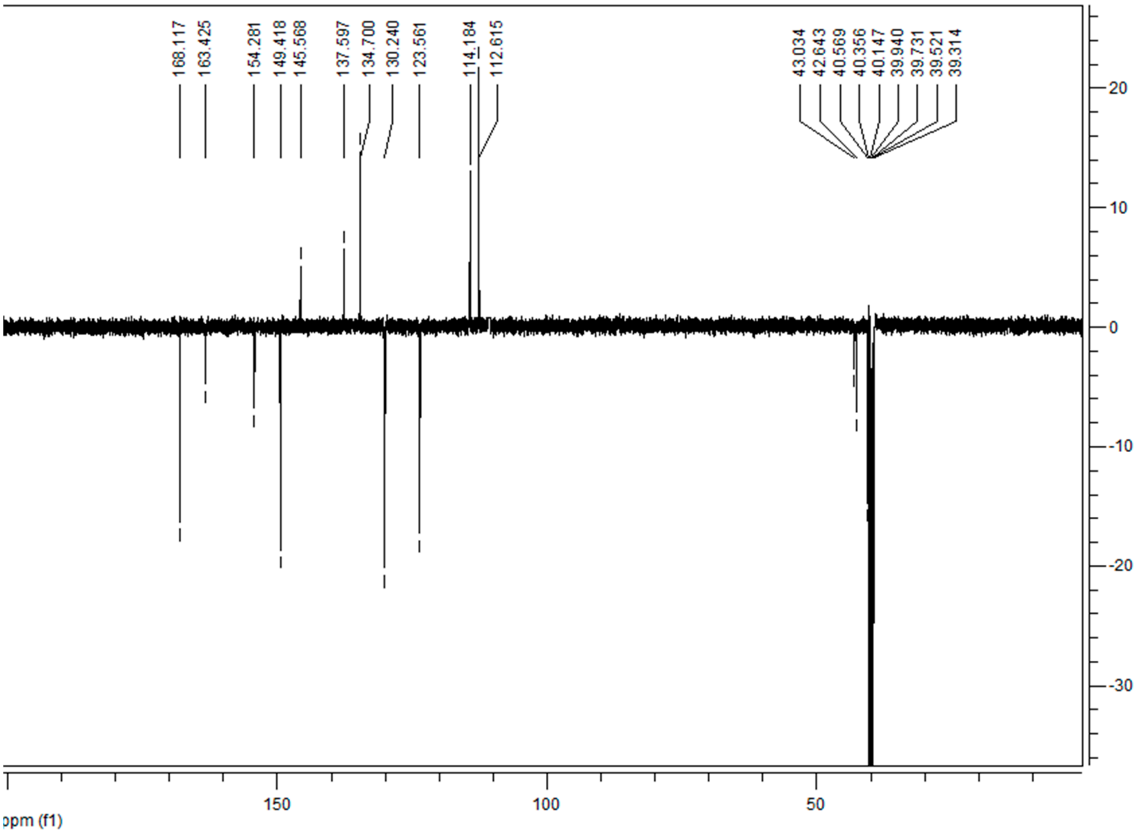

Figure S11. ^{13}C NMR spectra of compound **4**

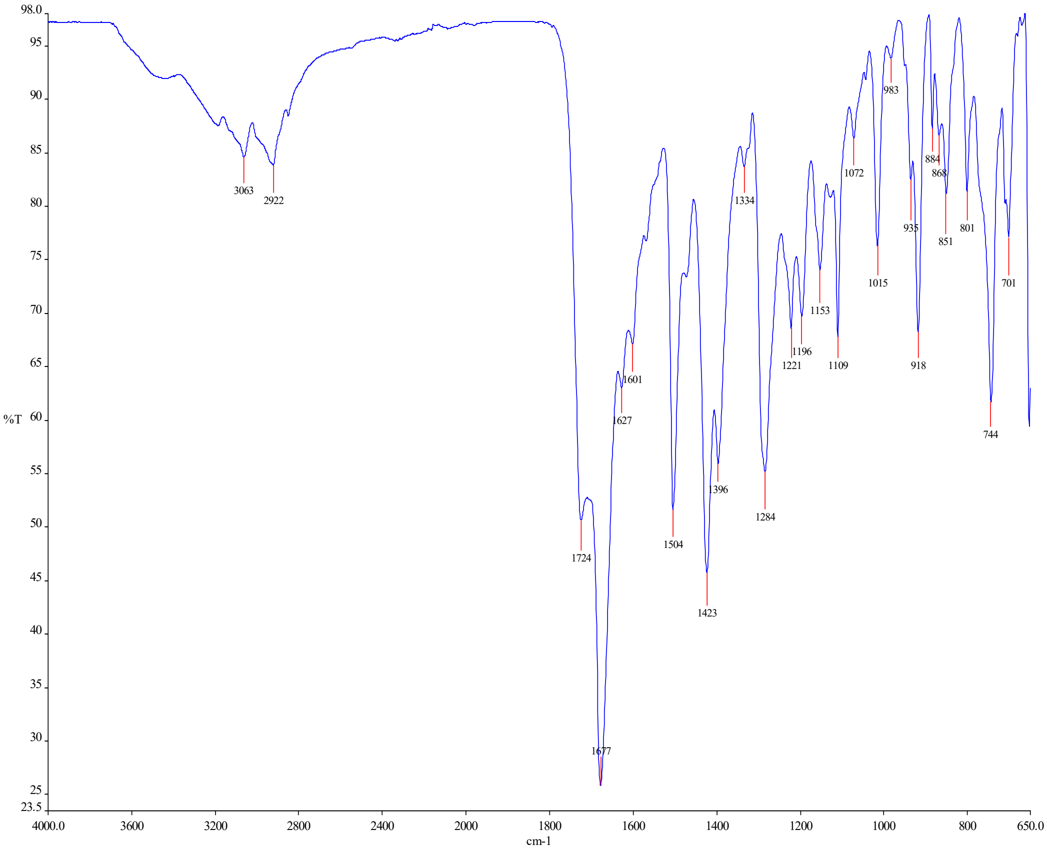

Figure S12. IR spectra of compound **5**

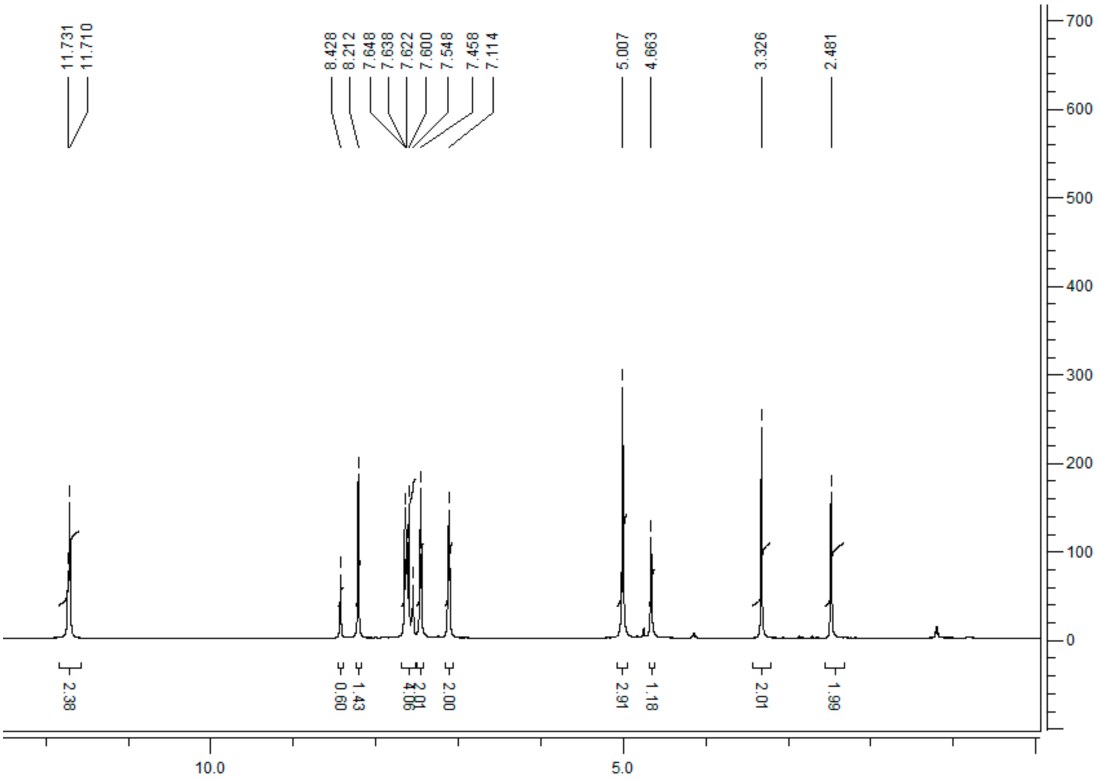

Figure S13. ^{1}H NMR spectra of compound **5**

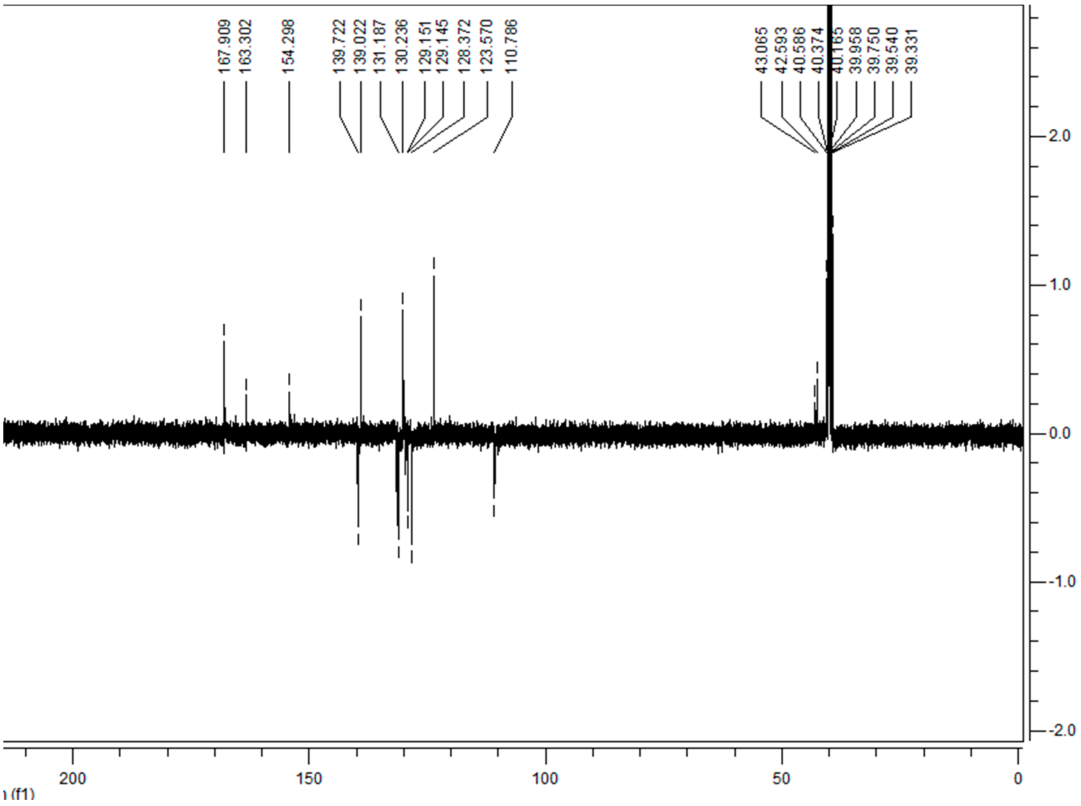

Figure S14. ^{13}C NMR spectra of compound **5**

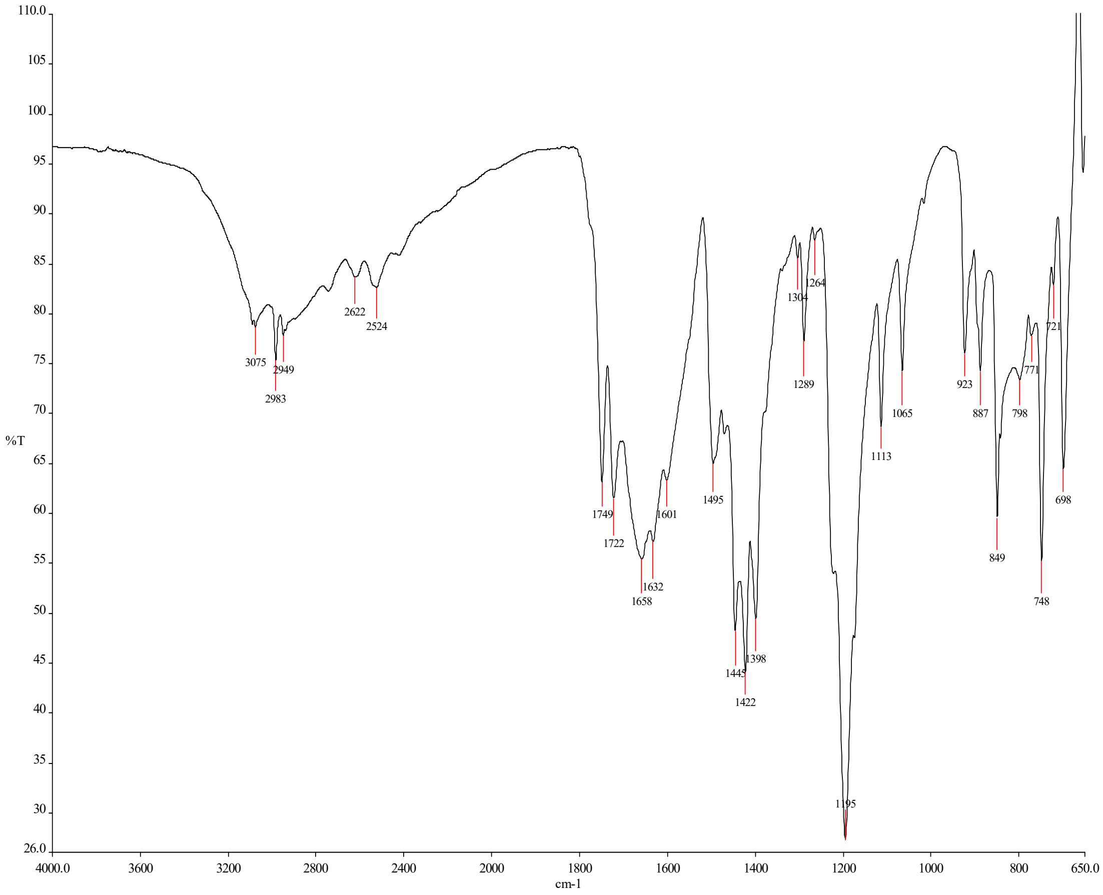

Figure S15. IR spectra of compound **6**

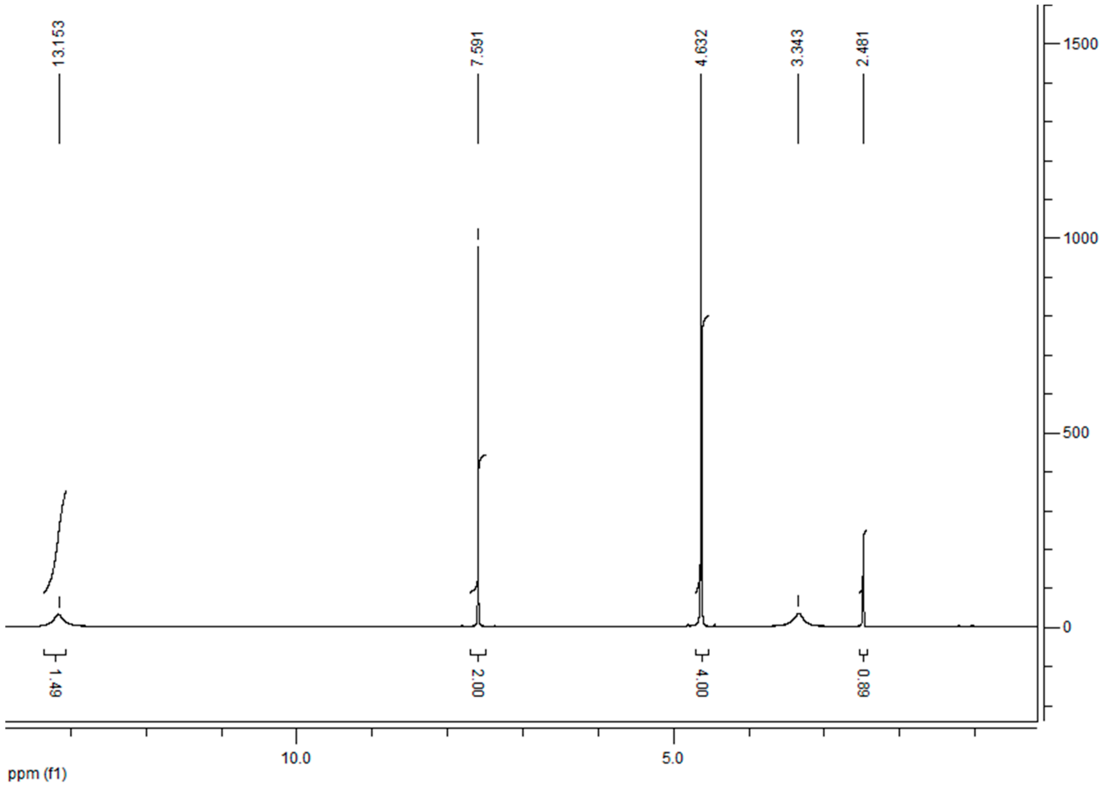

Figure S16. ^{1}H NMR spectra of compound **6**

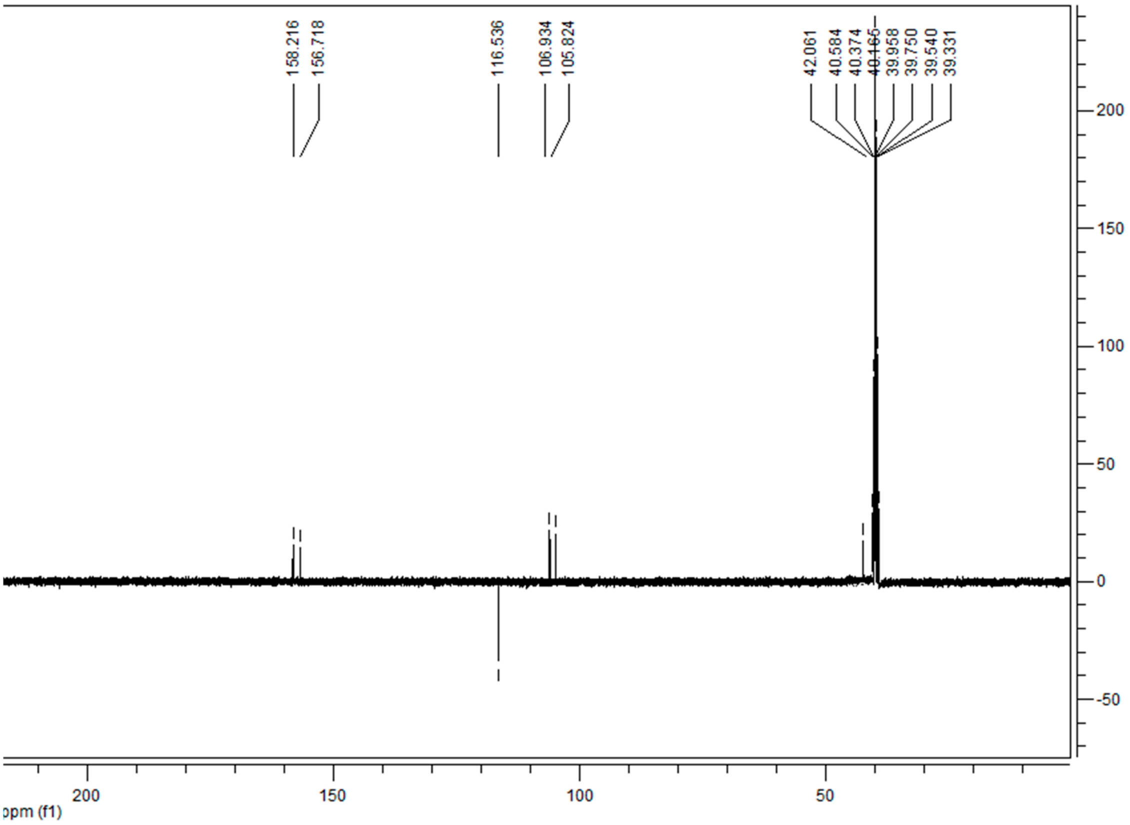

Figure S17. ^{13}C NMR spectra of compound **6**

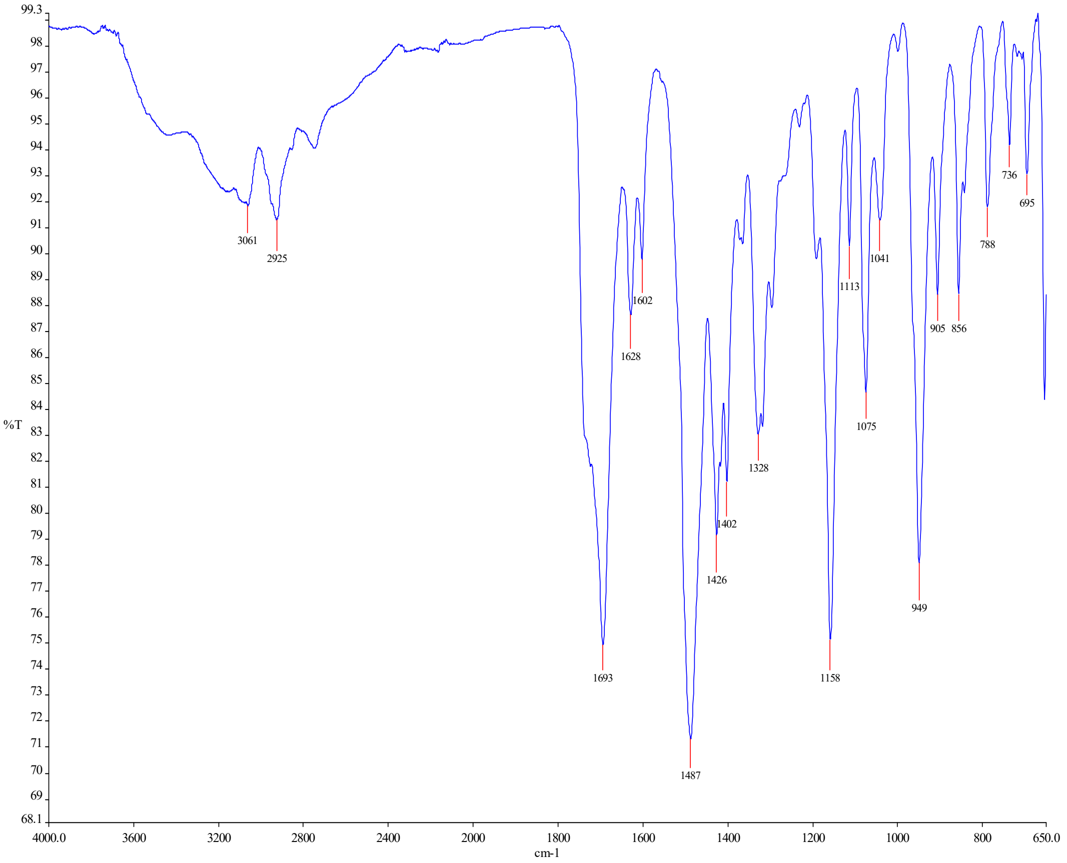

Figure S18. IR spectra of compound **7**

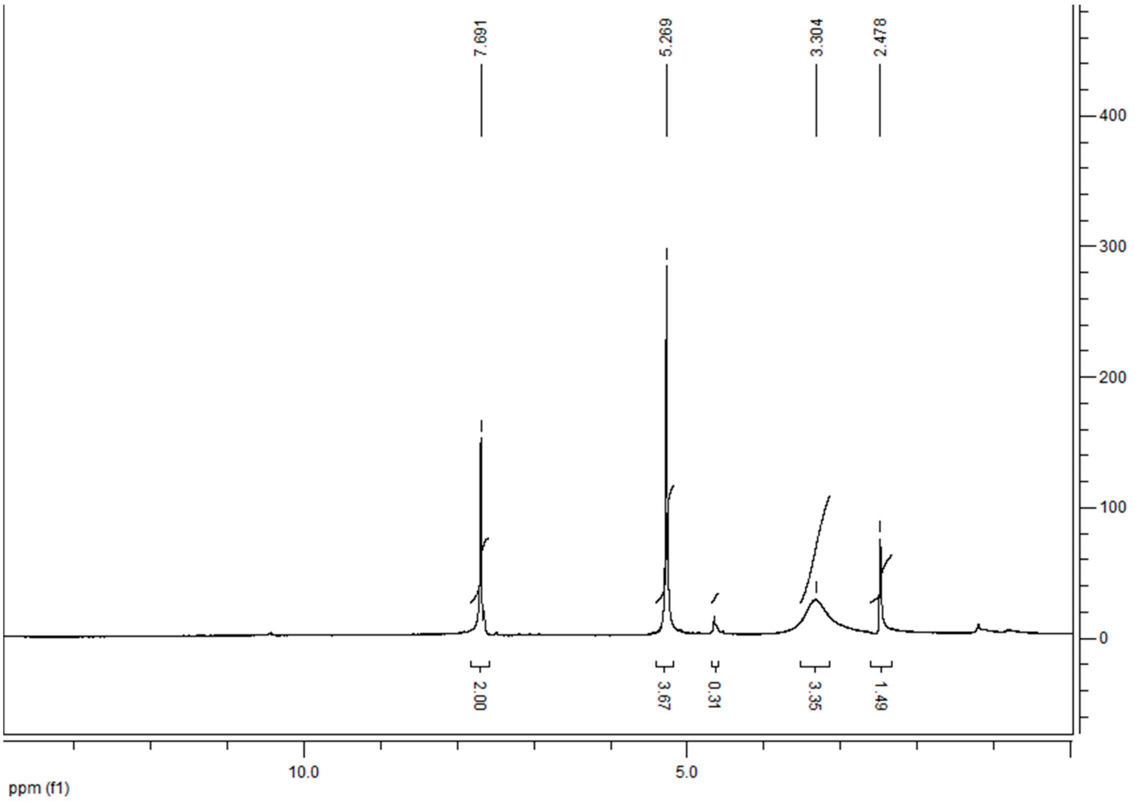

Figure S19. ^{1}H NMR spectra of compound **7**

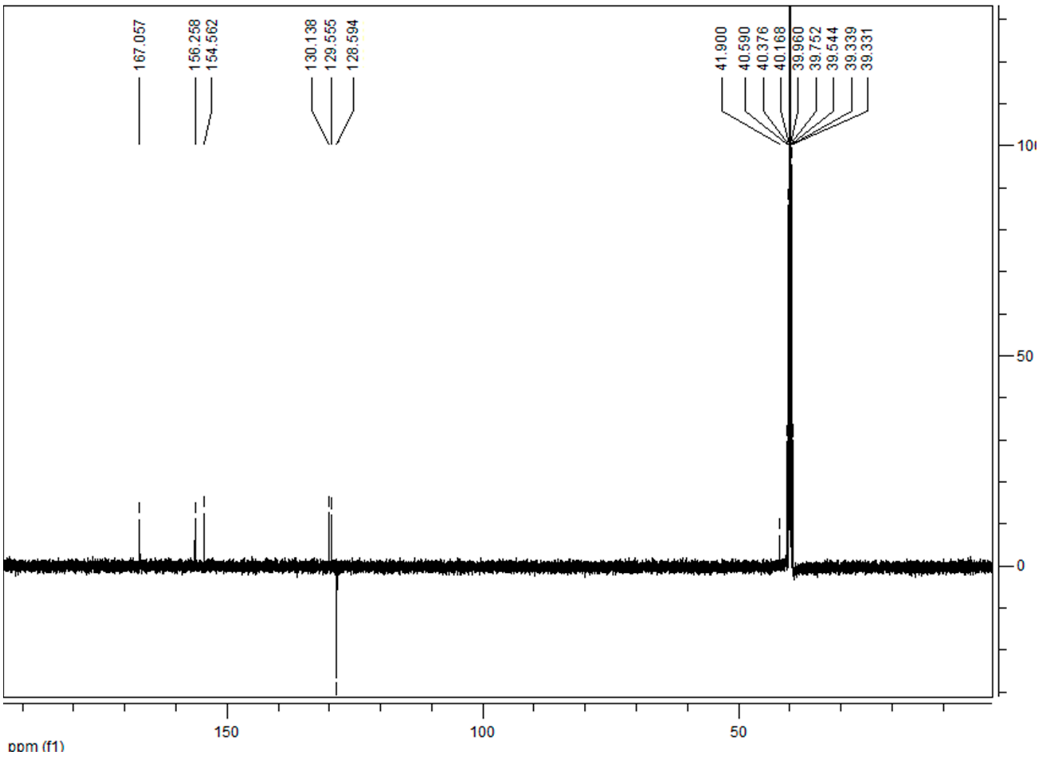

Figure S20. ^{13}C NMR spectra of compoιund **7**

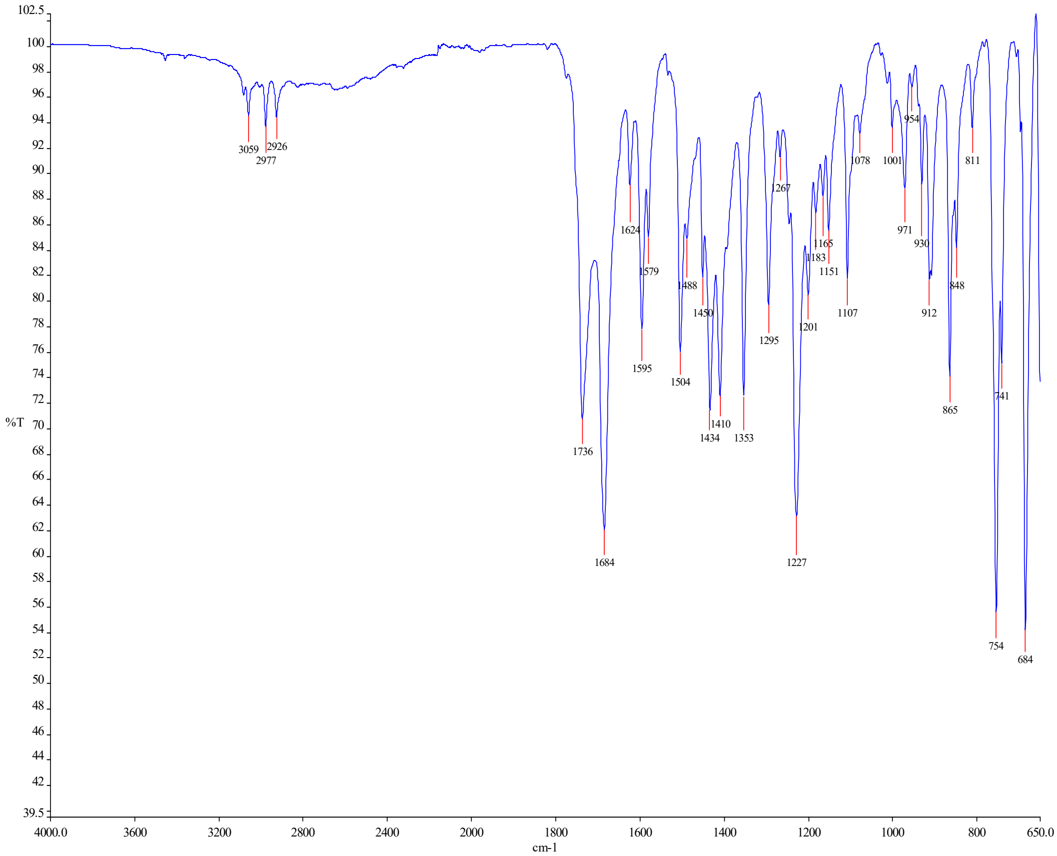

Figure S21. IR spectra of compound **8**

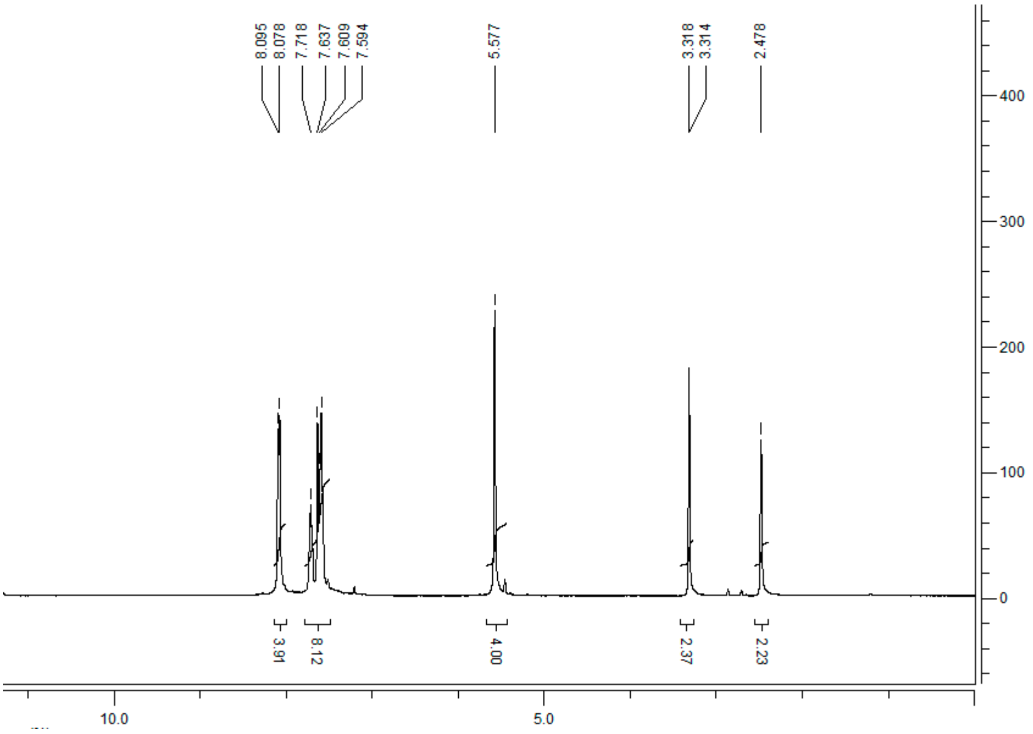

Figure S22. ^{1}H NMR spectra of compound **8**

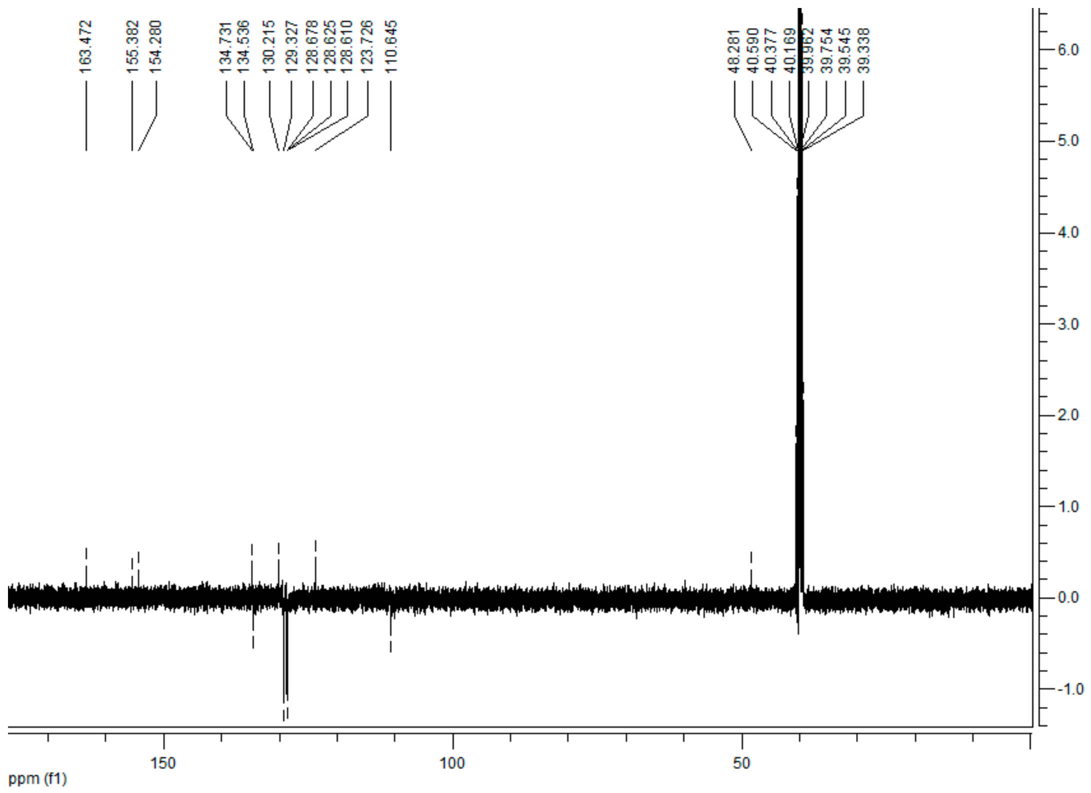

Figure S23. ^{13}C NMR spectra of compound **8**

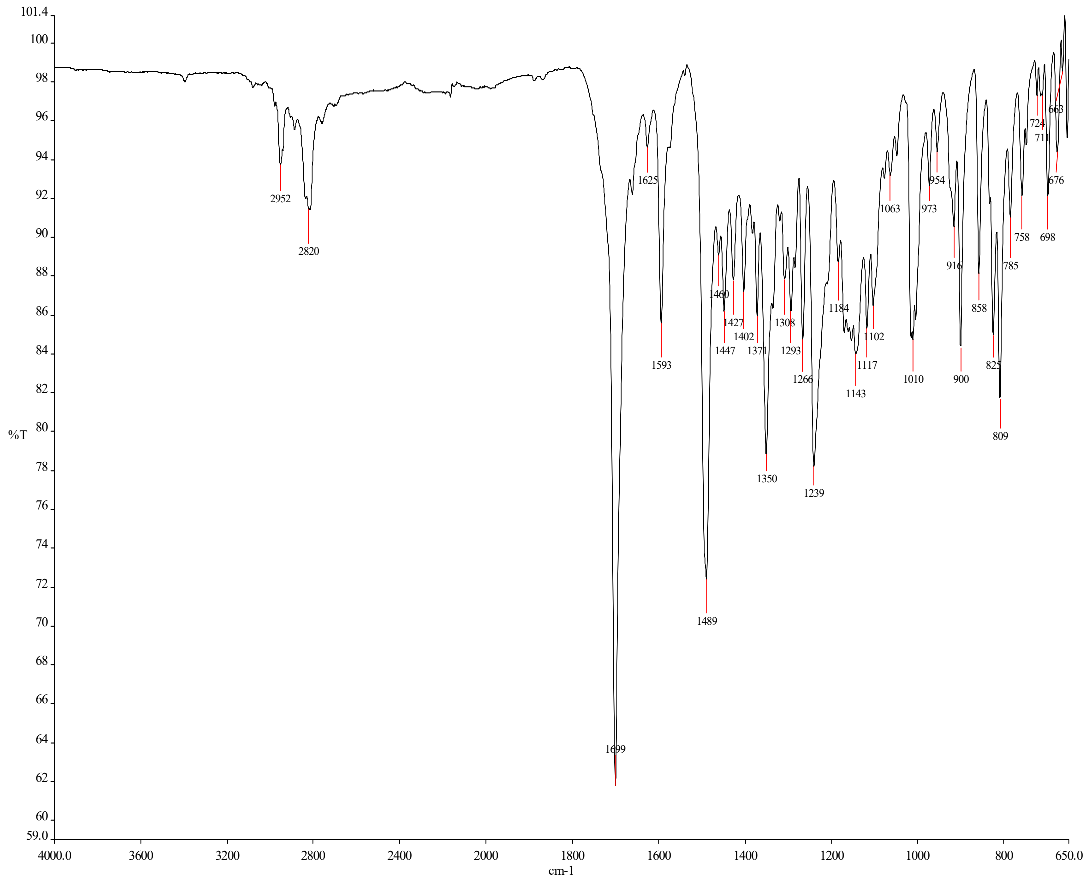

Figure S24. IR spectra of compound **9**

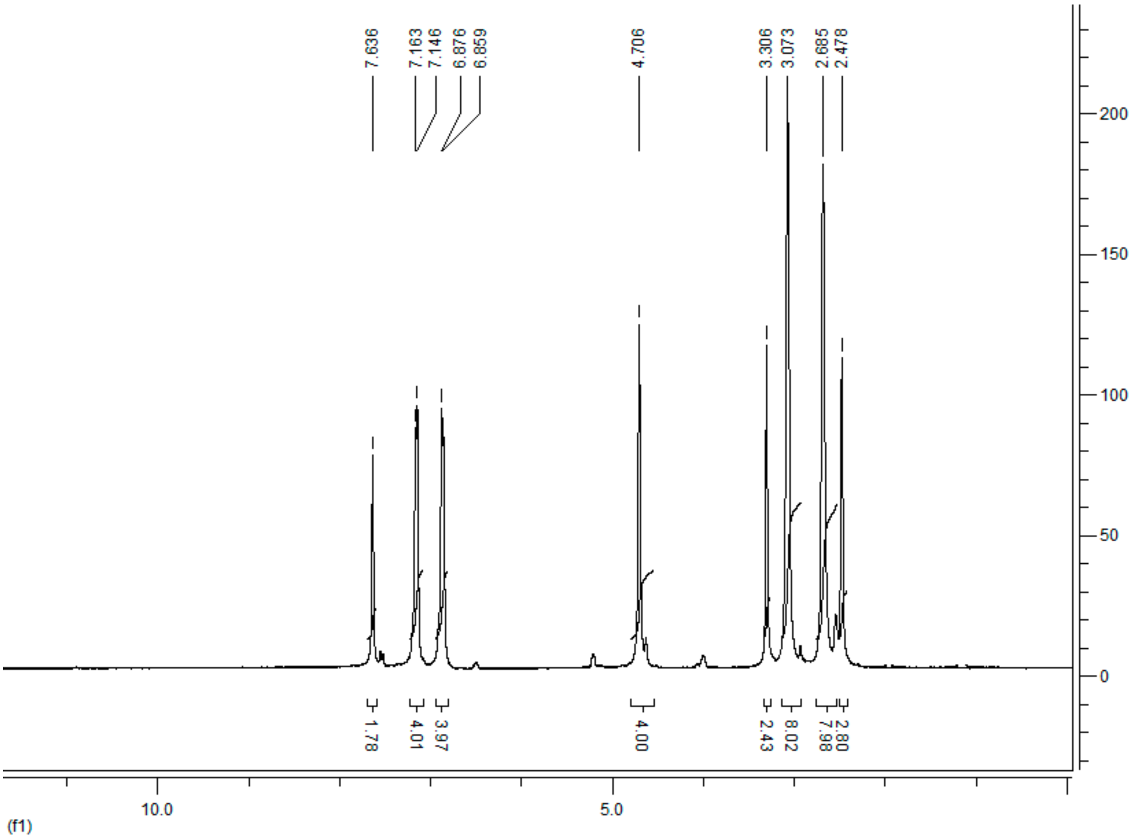

Figure S25. ^{1}H NMR spectra of compound **9**

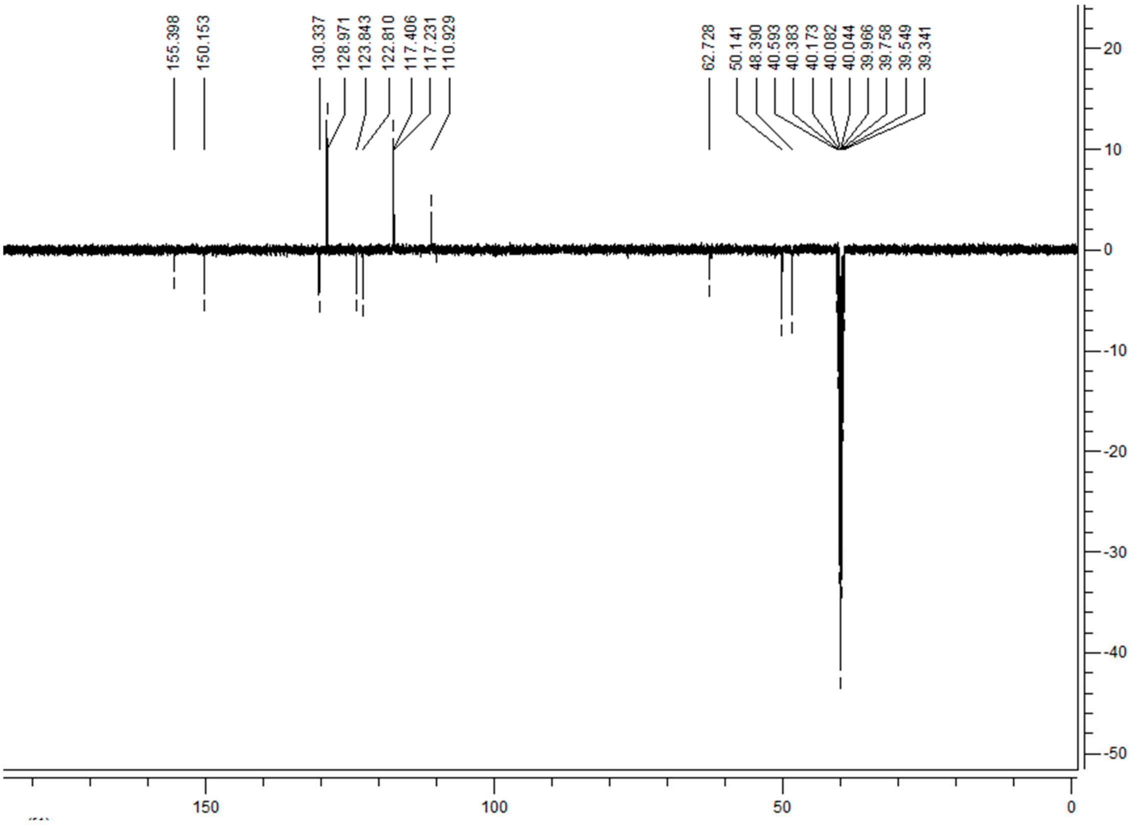

Figure S26. ^{13}C NMR spectra of compound **9**

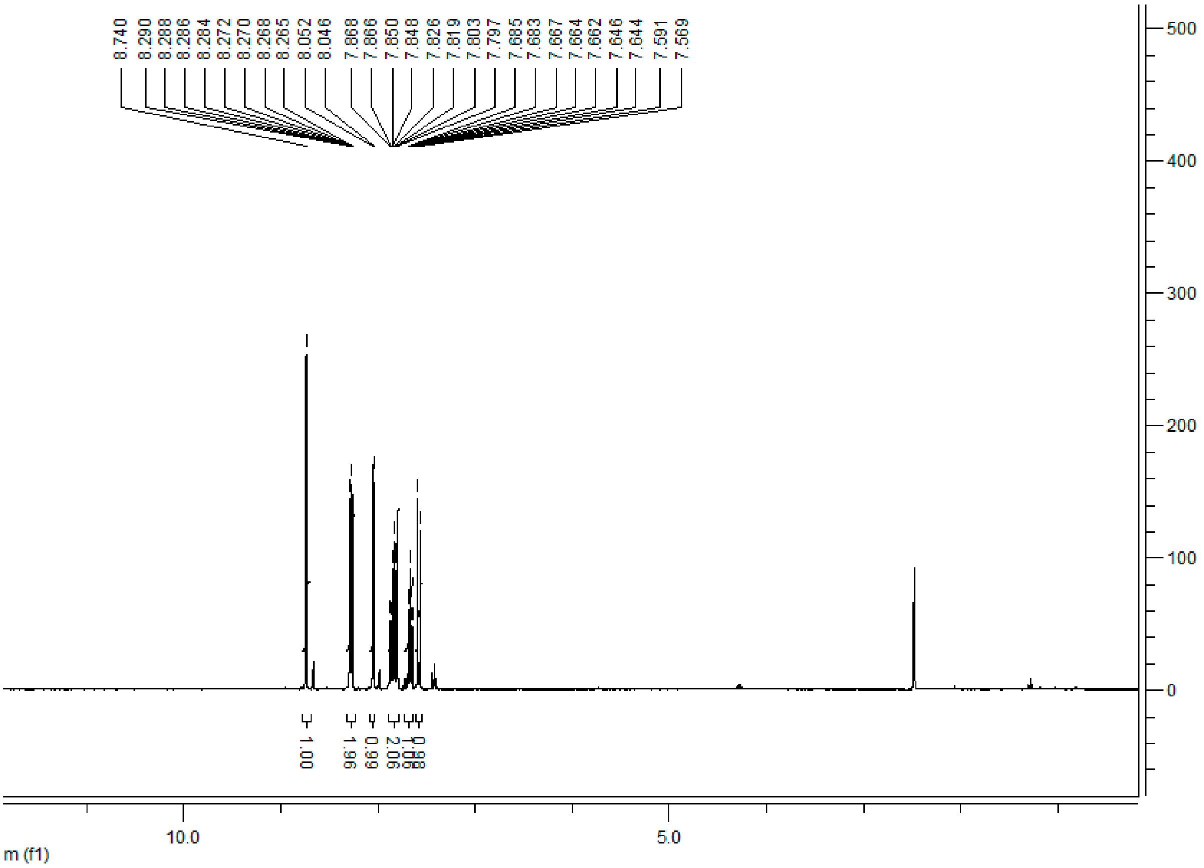

Figure S27. ^{1}H NMR spectra of compound **11b**

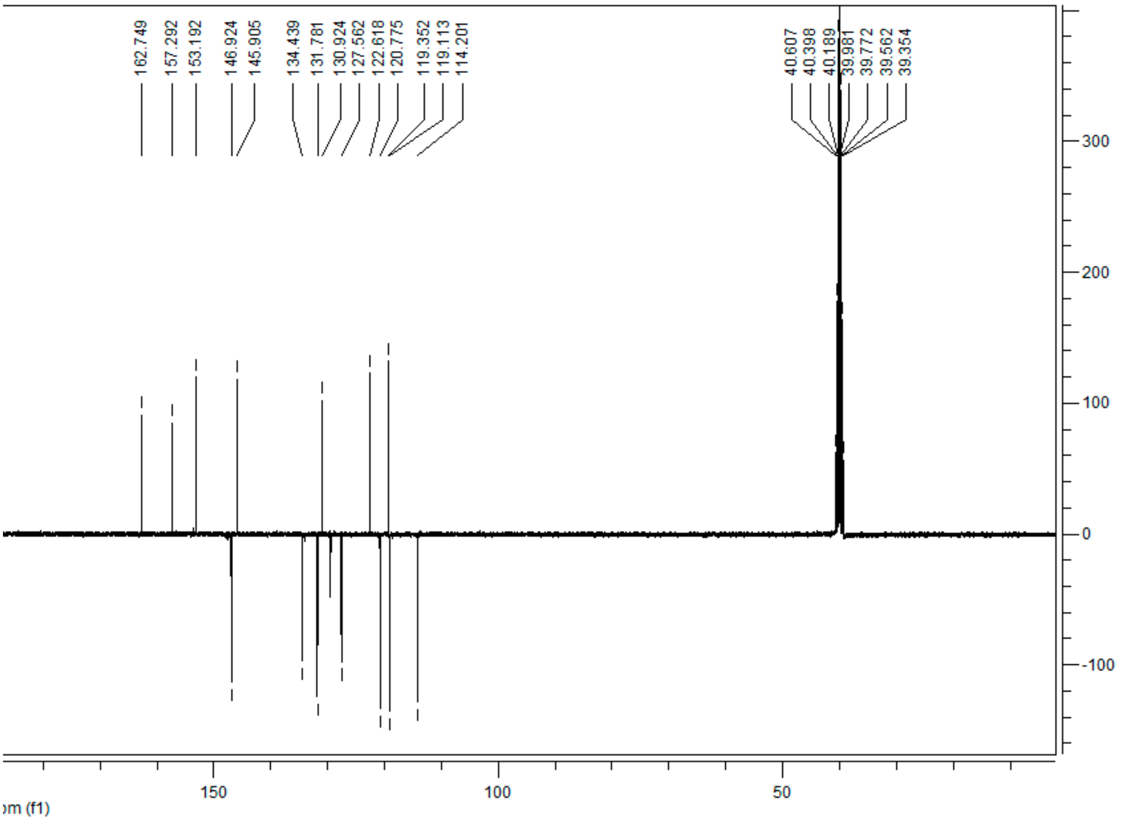

Figure S28. ^{13}C NMR spectra of compound **11b**

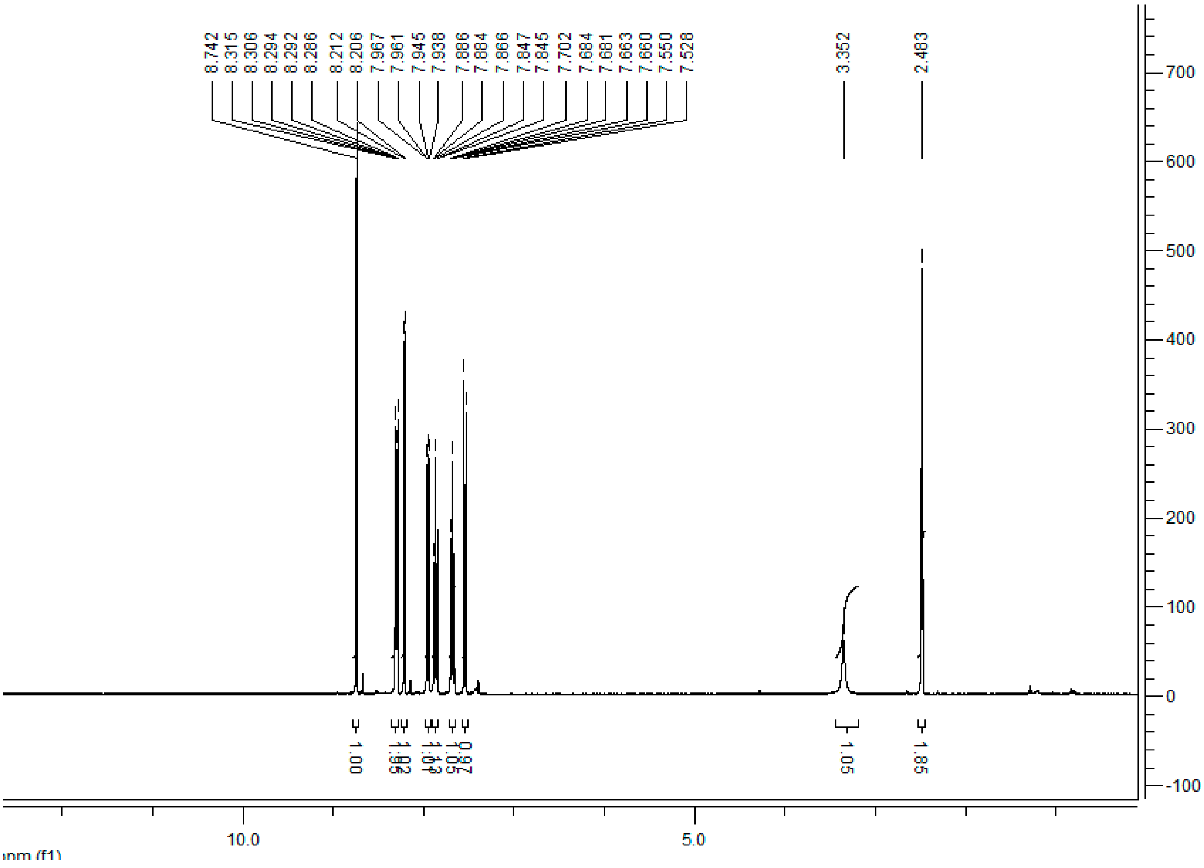

Figure S29. ^{1}H NMR spectra of compound **11c**

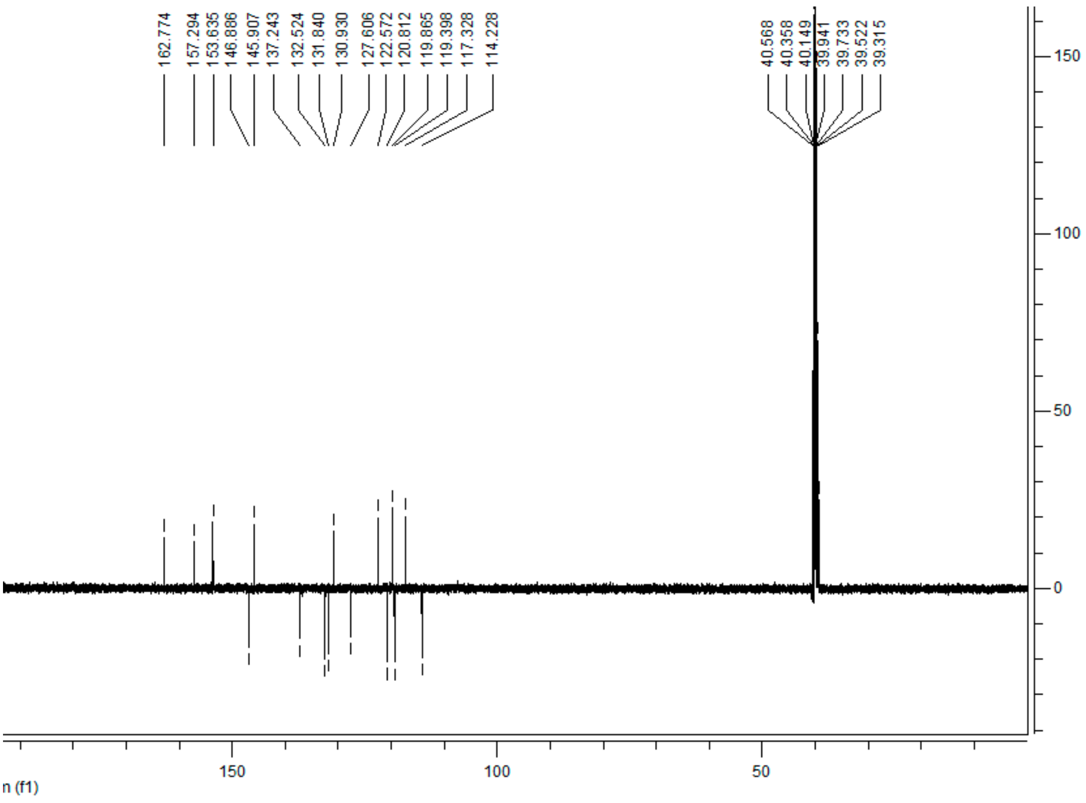

Figure S30. ^{13}C NMR spectra of compound **11c**

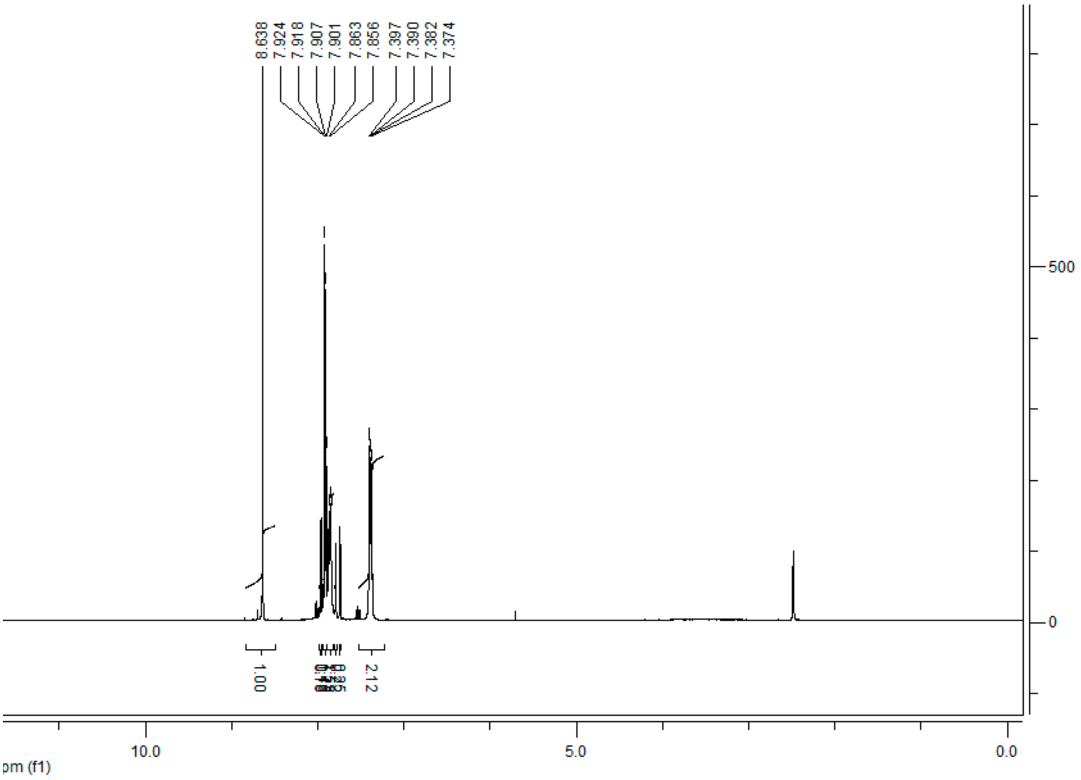

Figure S31. ^{1}H NMR spectra of compound **11d**

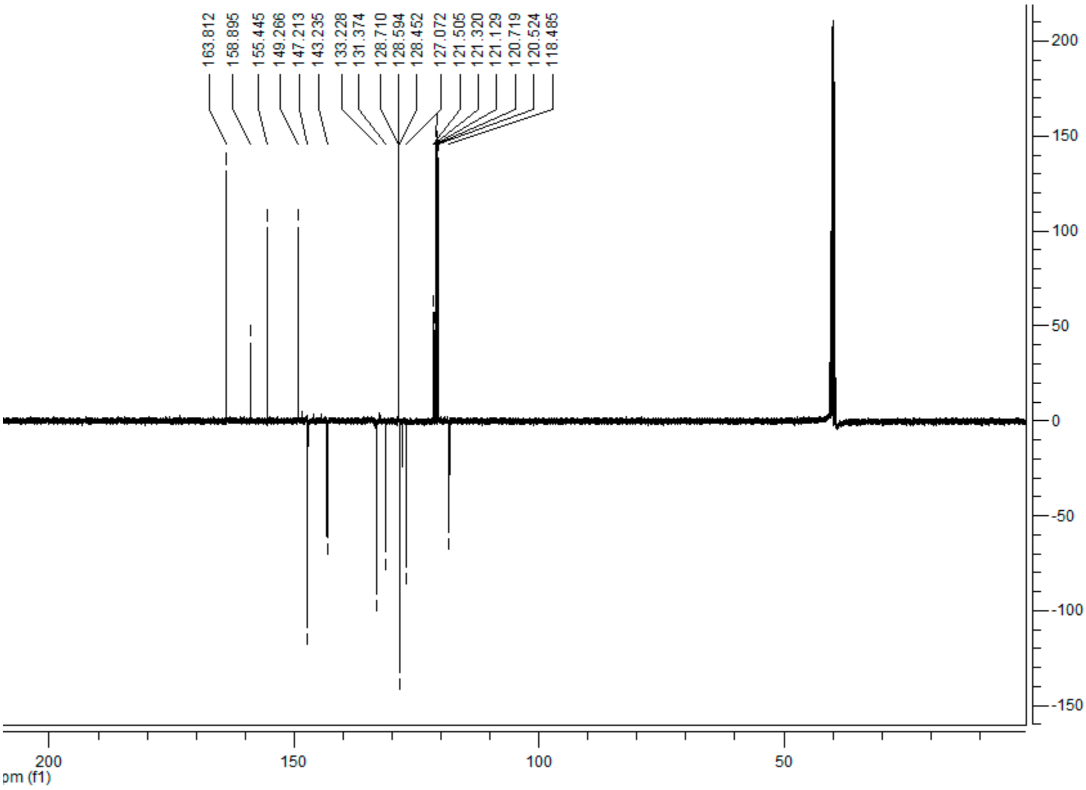

Figure S32. ^{13}C NMR spectra of compound **11d**

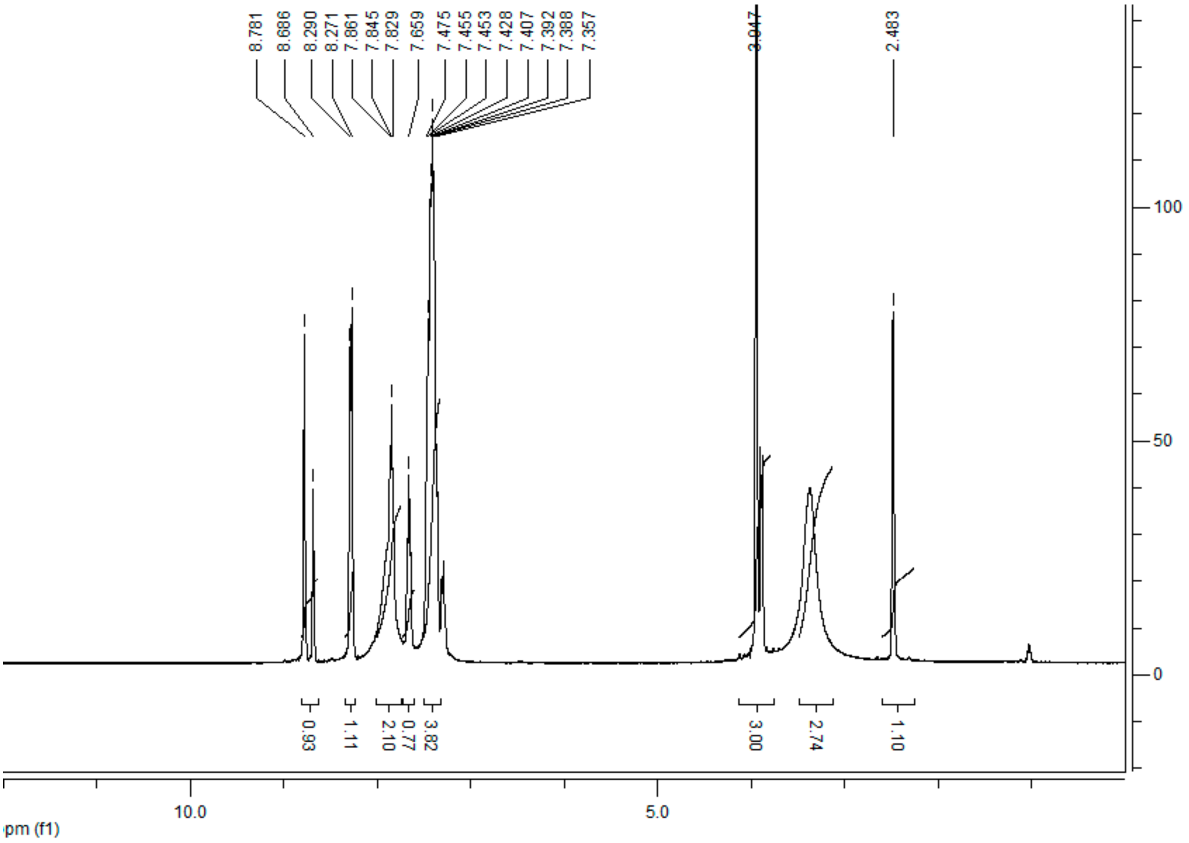

Figure S33. ^{1}H NMR spectra of compound **11e**

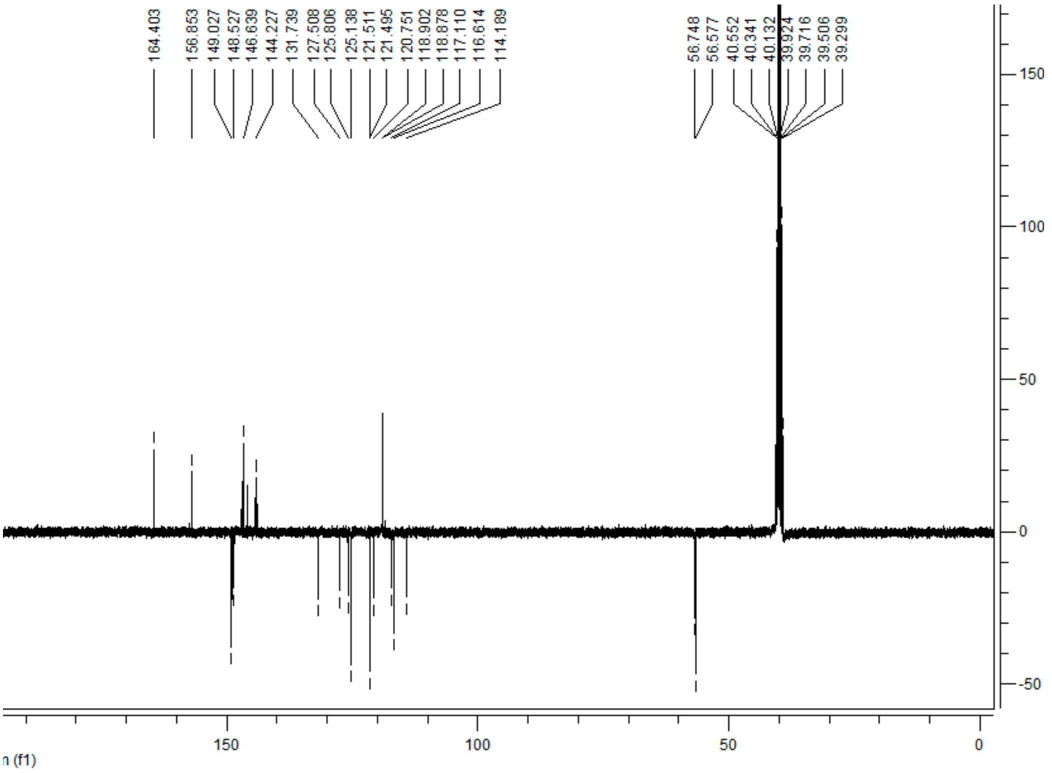

Figure S34. ^{13}C NMR spectra of compound **11e**

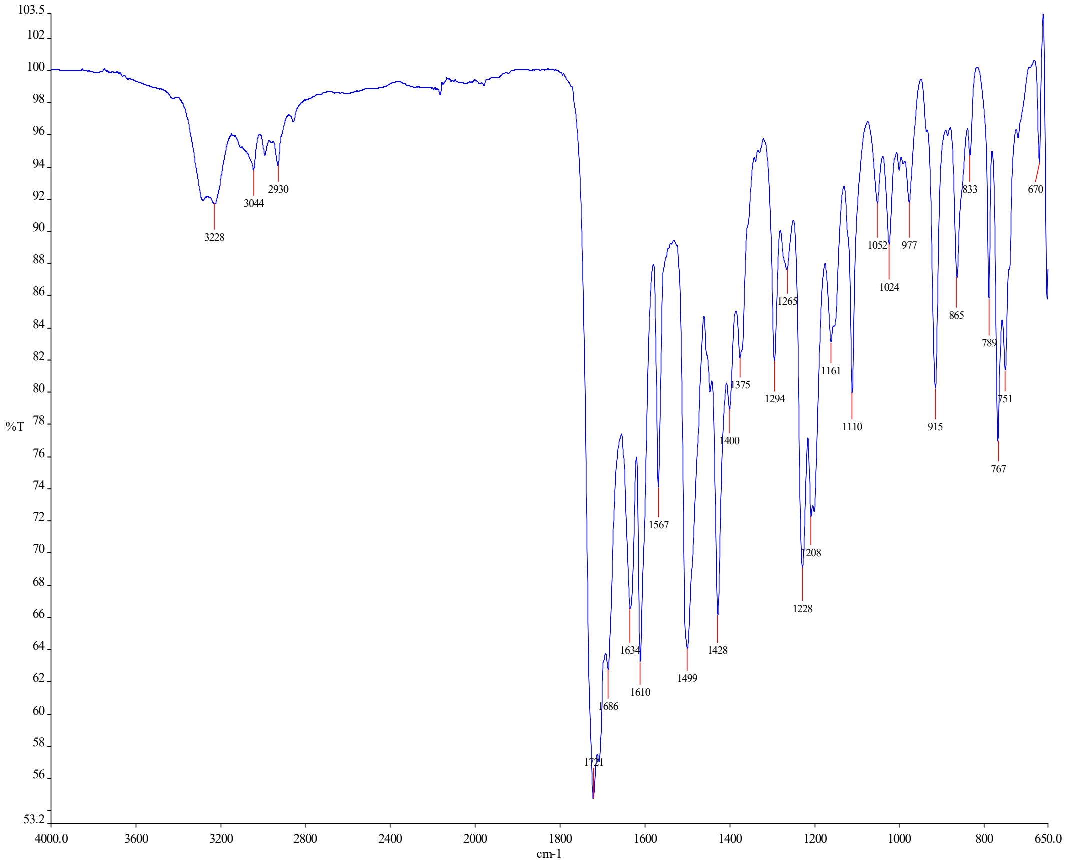

Figure S35. IR spectra of compound **12a**

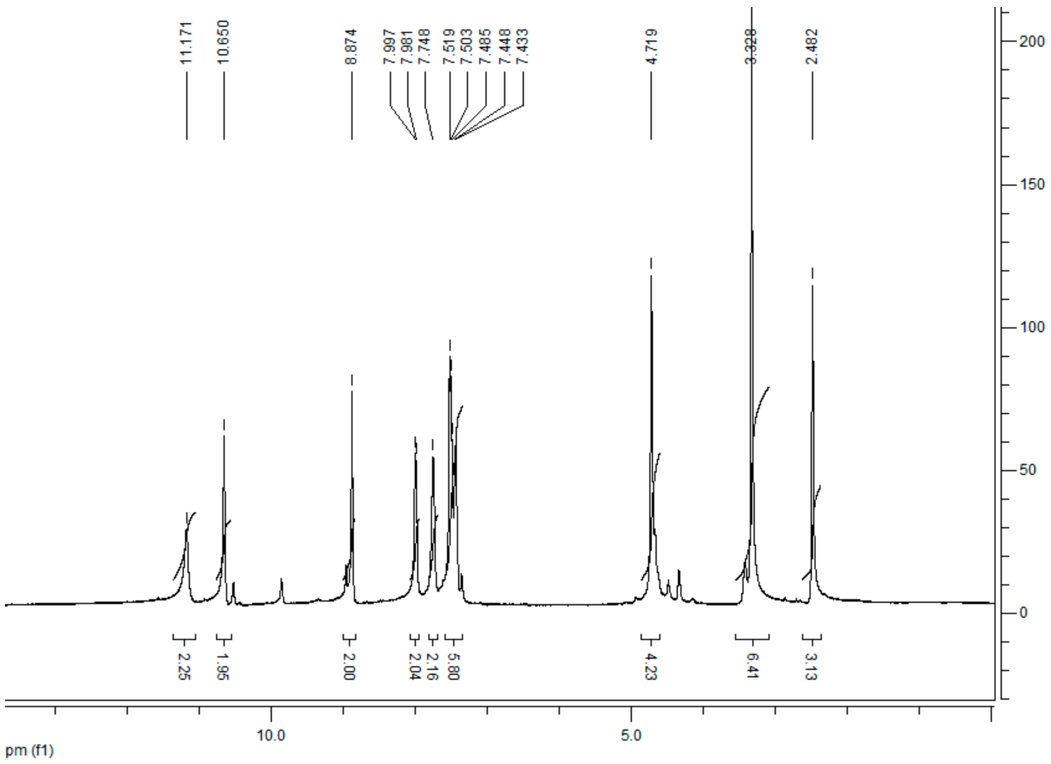

Figure S36. ^{1}H NMR spectra of compound **12a**

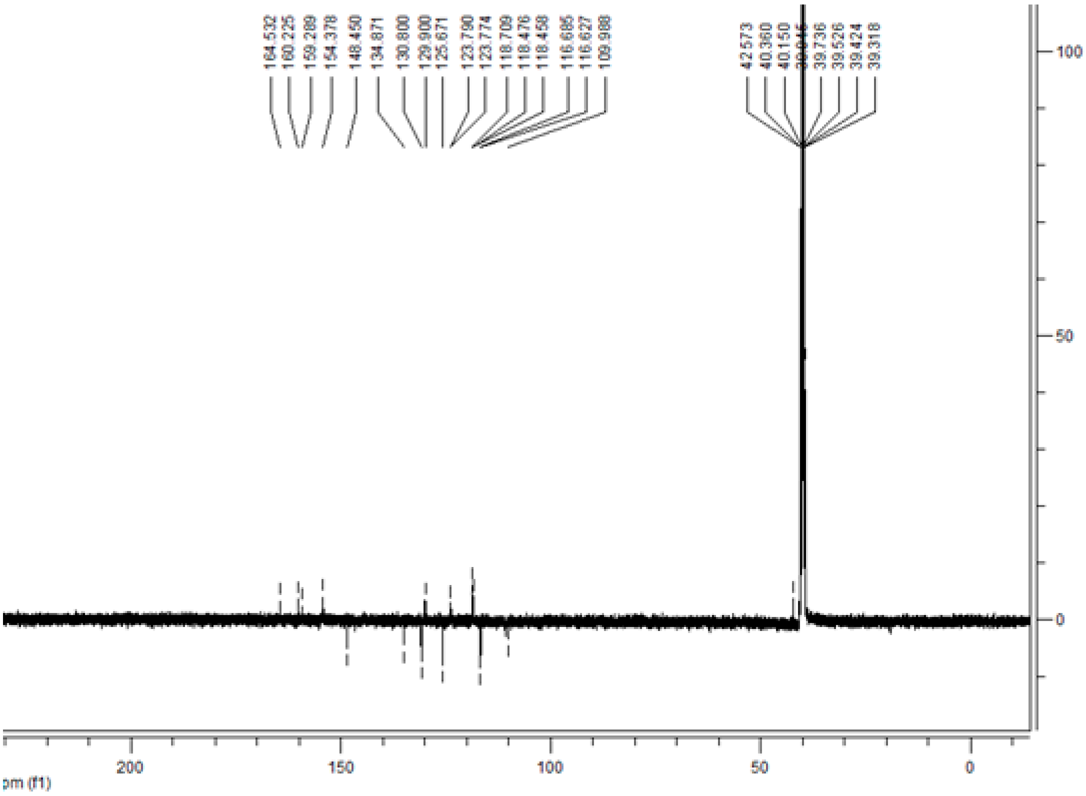

Figure S37. ^{13}C NMR spectra of compound **12a**

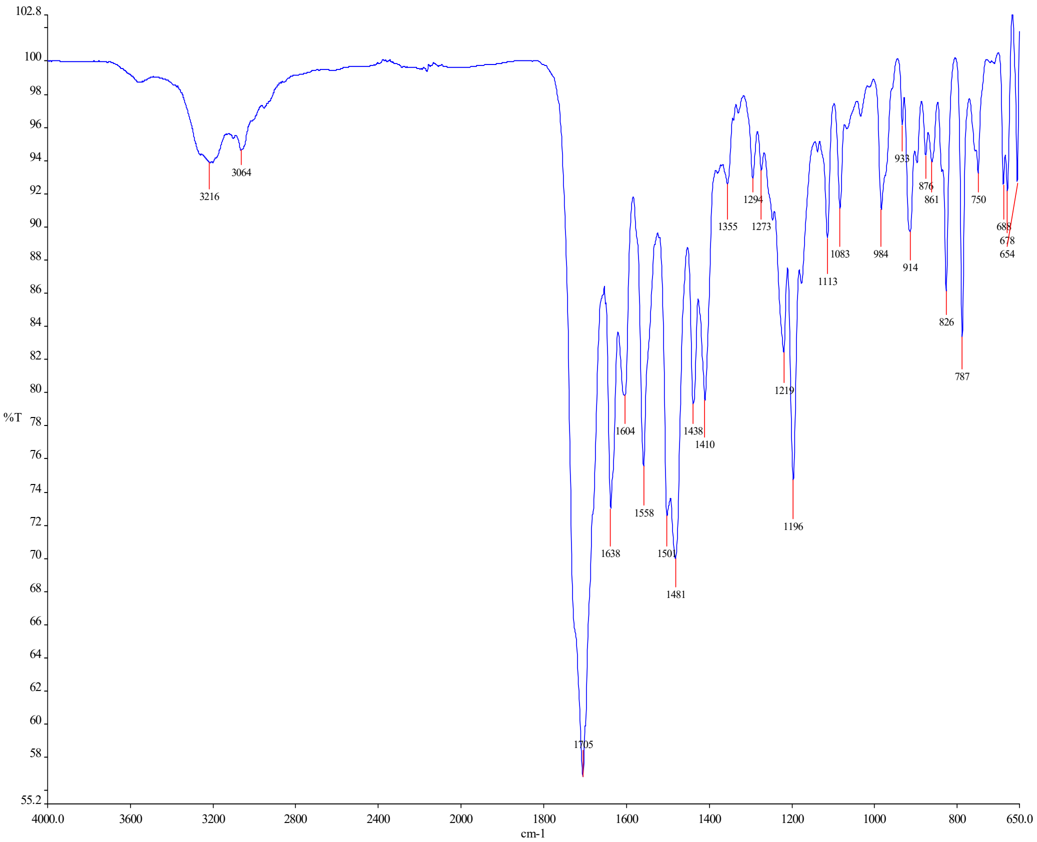

Figure S38. IR spectra of compound **12b**

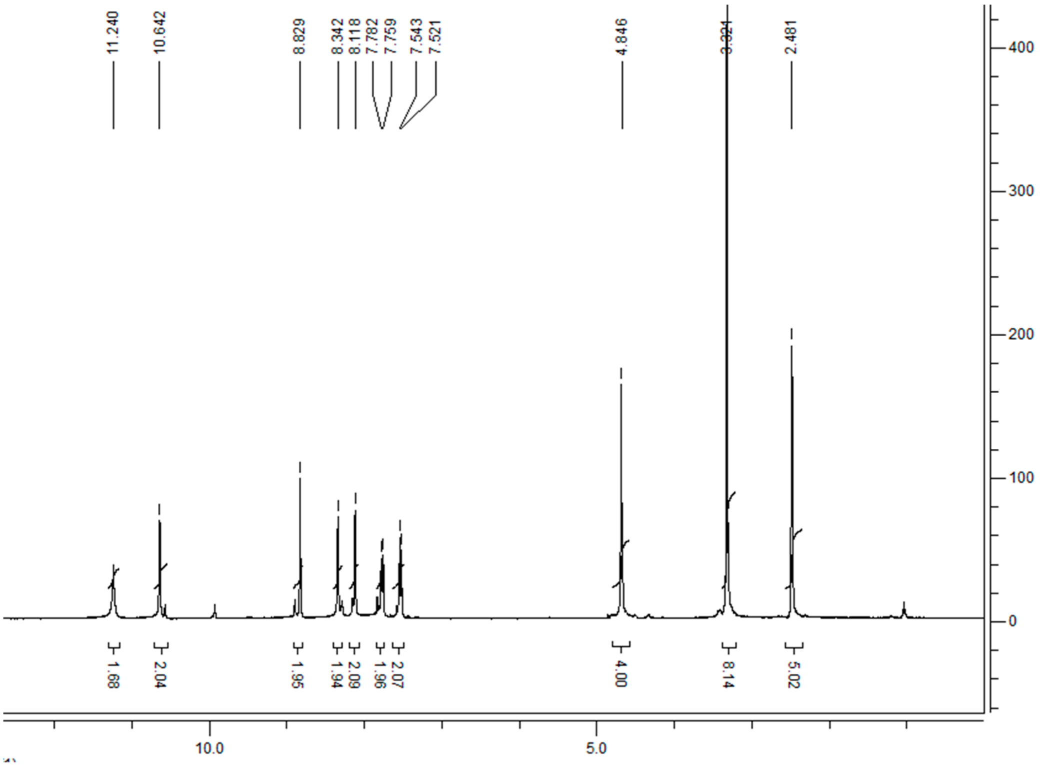

Figure S39. ^{1}H NMR spectra of compound **12b**

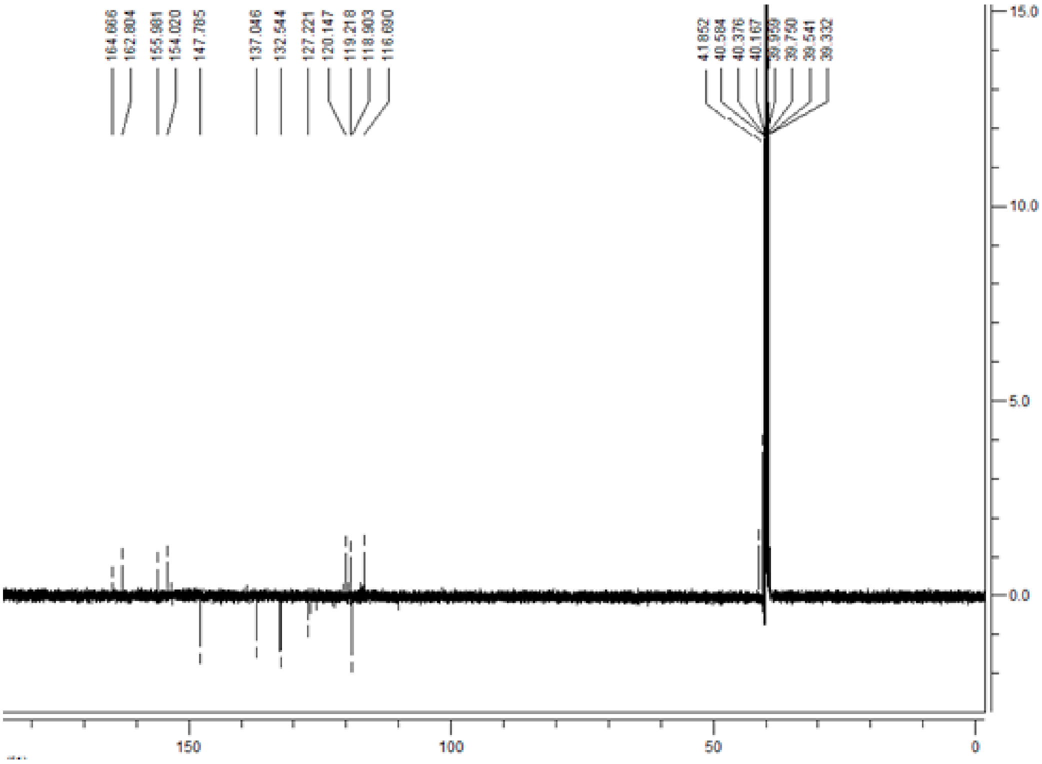

Figure S40. ^{13}C NMR spectra of compound **12b**

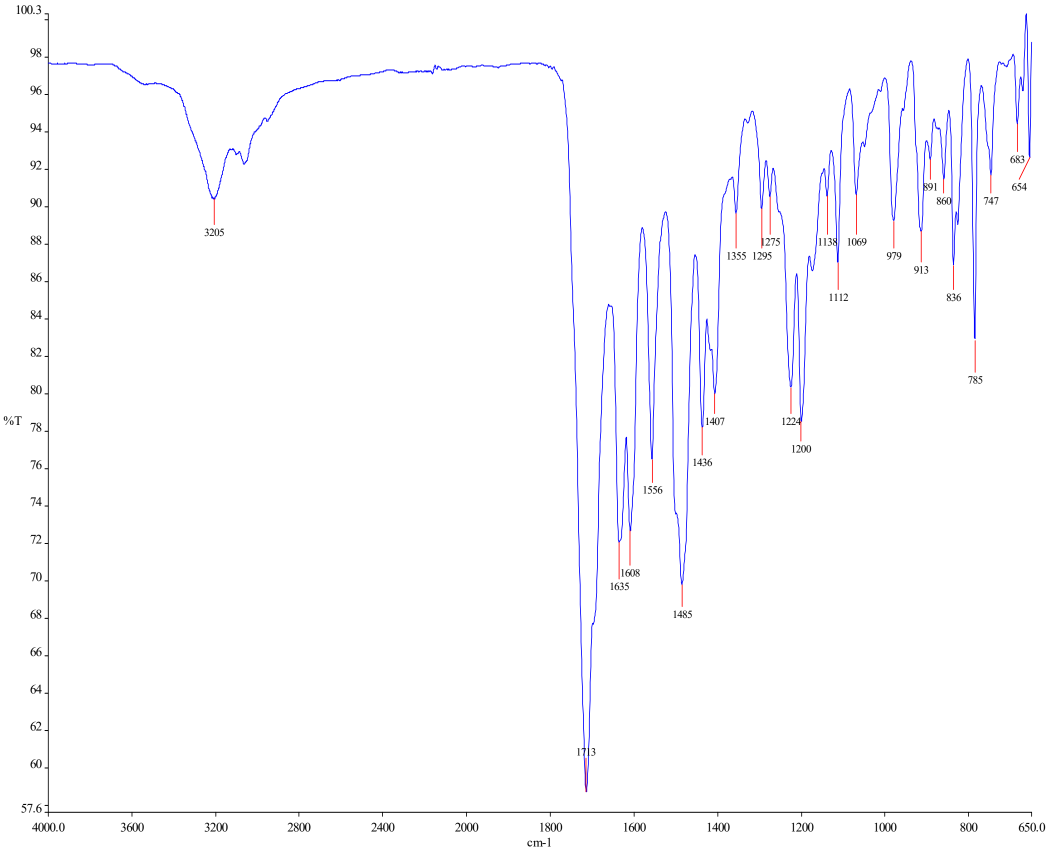

Figure S41. IR spectra of compound **12c**

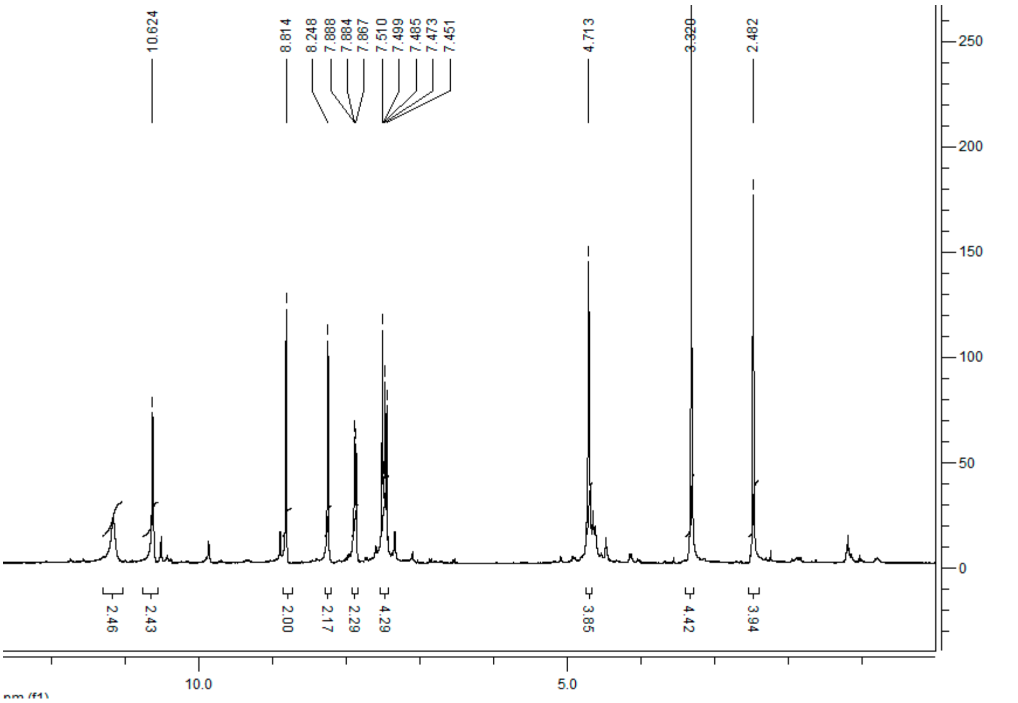

Figure S42. ^{1}H NMR spectra of compound **12c**

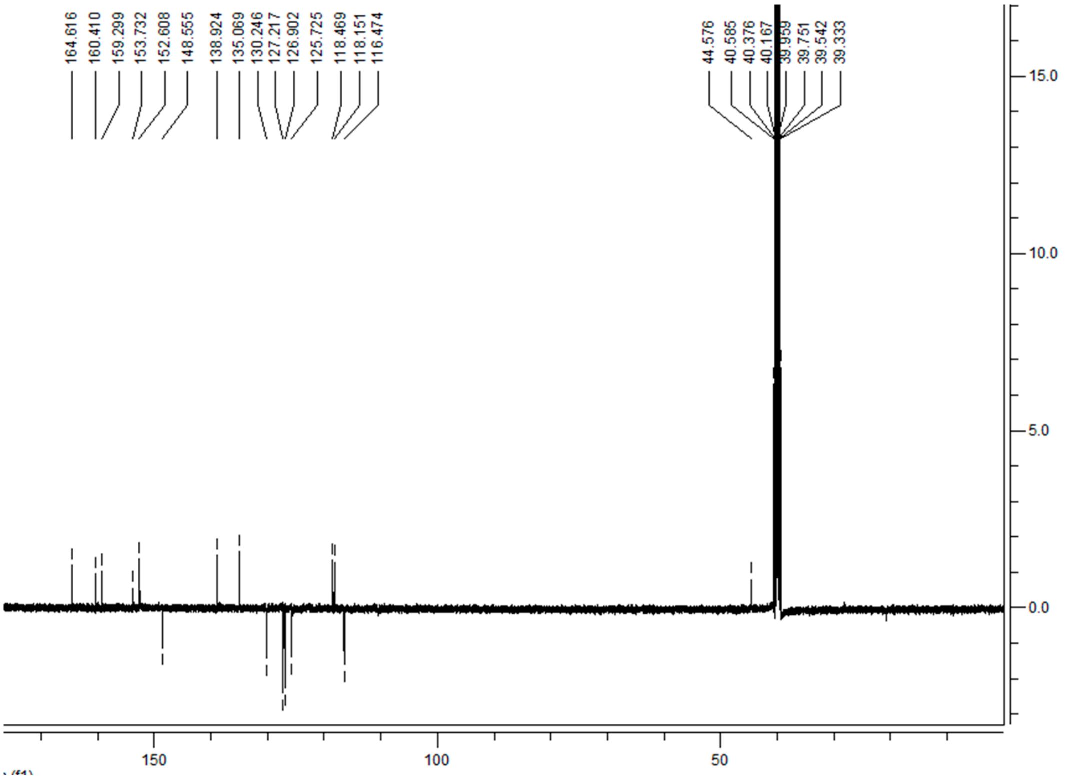

Figure S43. ^{13}C NMR spectra of compound **12c**

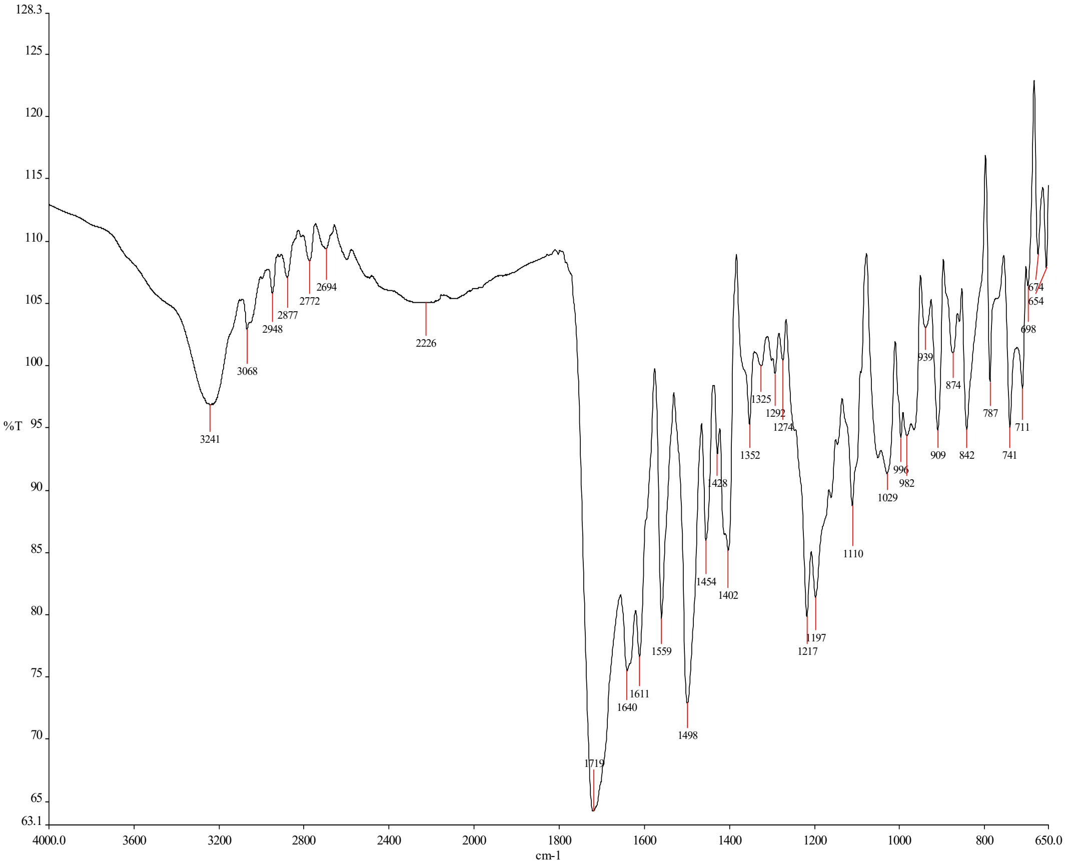

Figure S44. IR spectra of compound **12d**

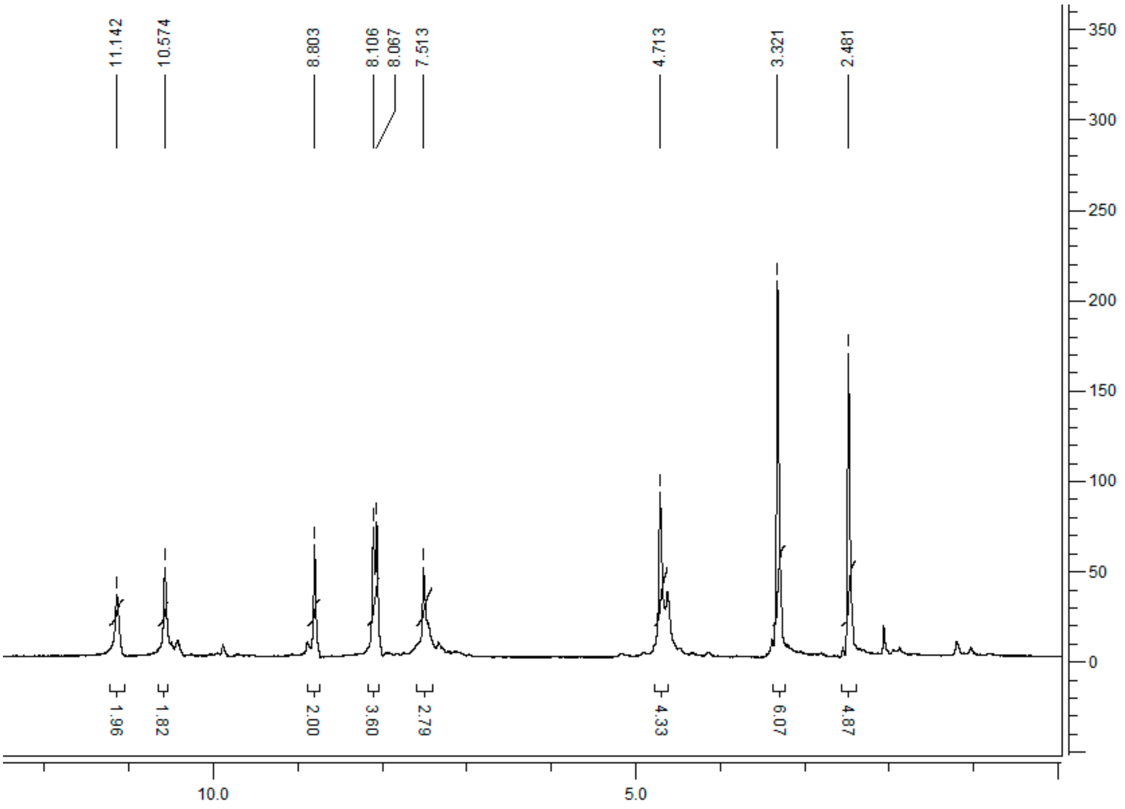

Figure S45. ^{1}H NMR spectra of compound **12d**

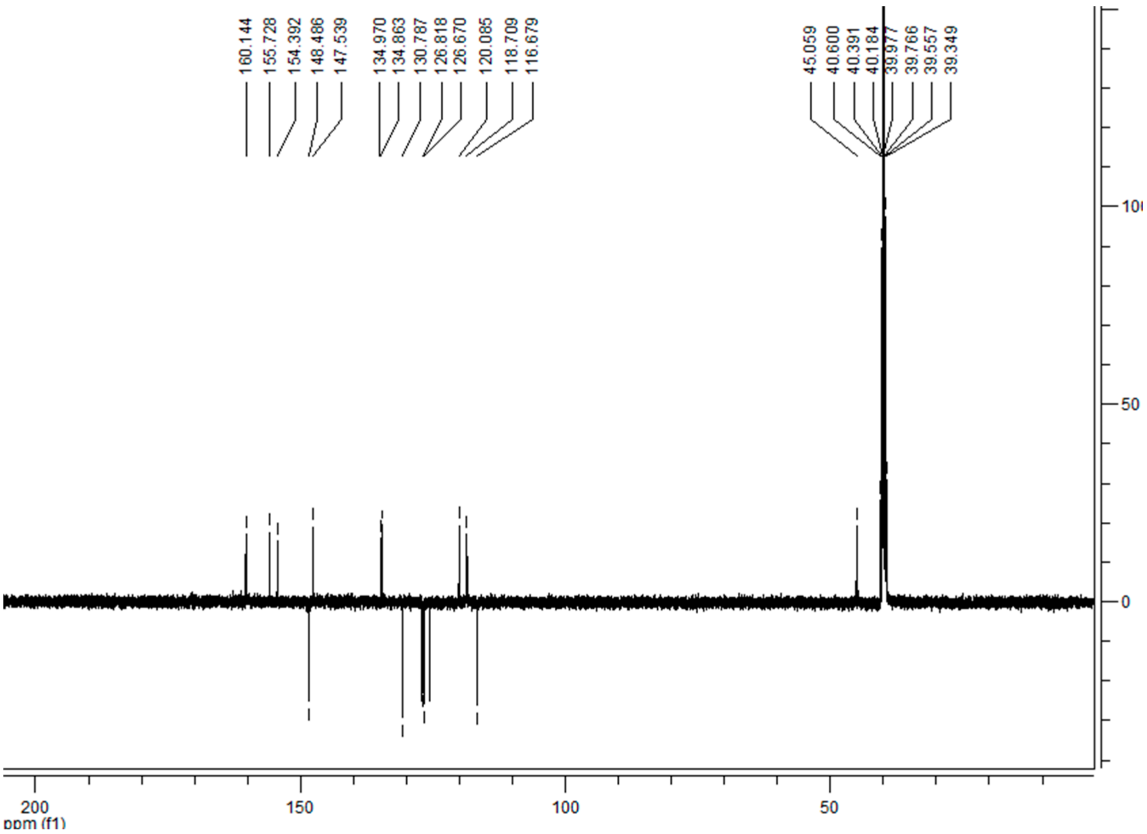

Figure S46. ^{13}C NMR spectra of compound **12d**

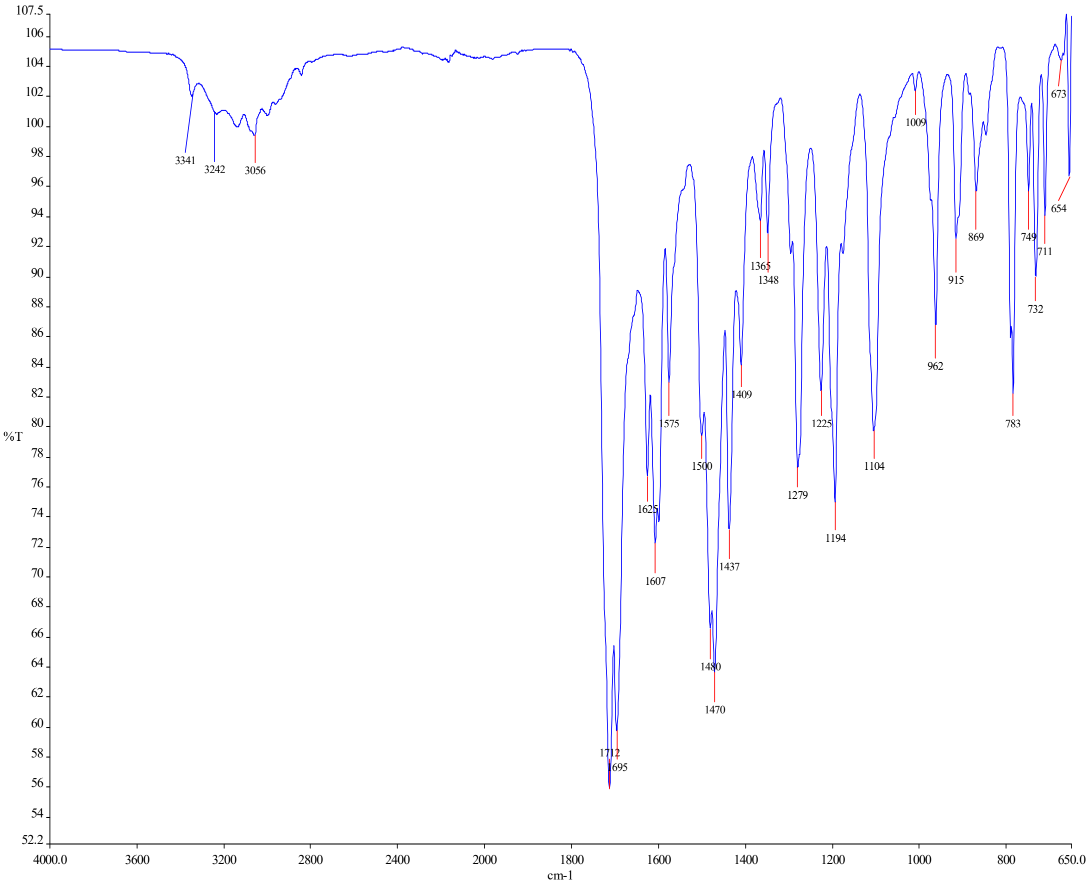

Figure S47. IR spectra of compound **12e**

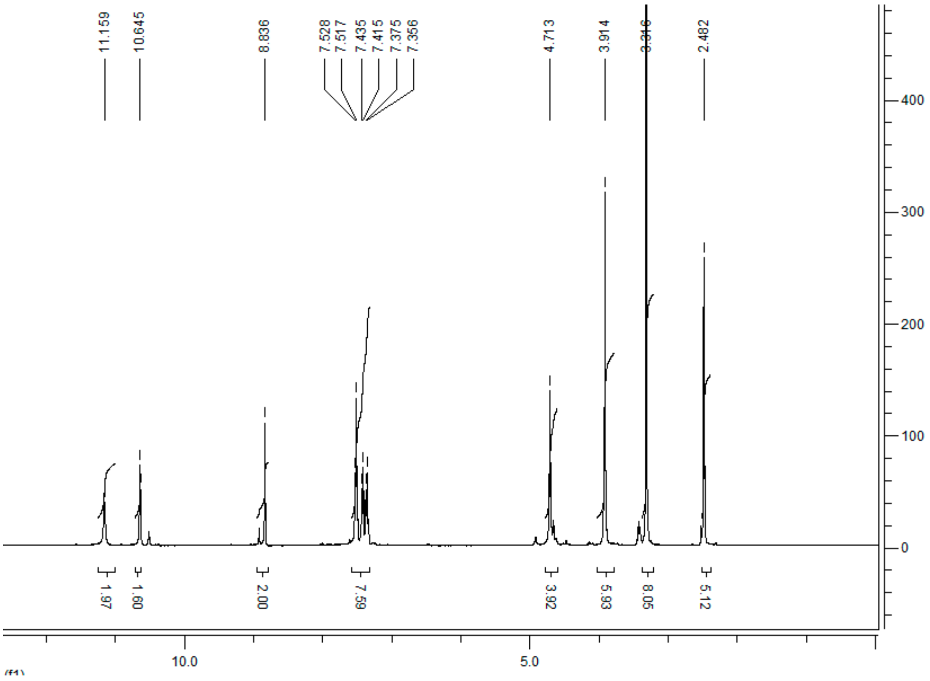

Figure S48. ^{1}H NMR spectra of compound **12e**

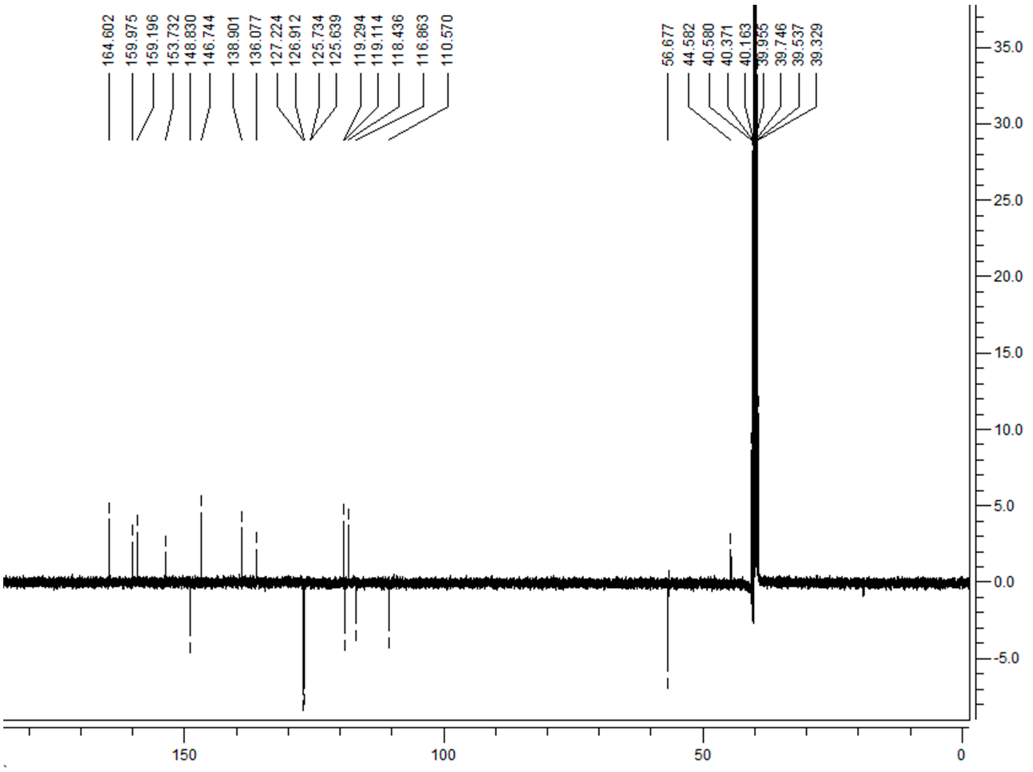

Figure S49. ^{13}C NMR spectra of compound **12e**

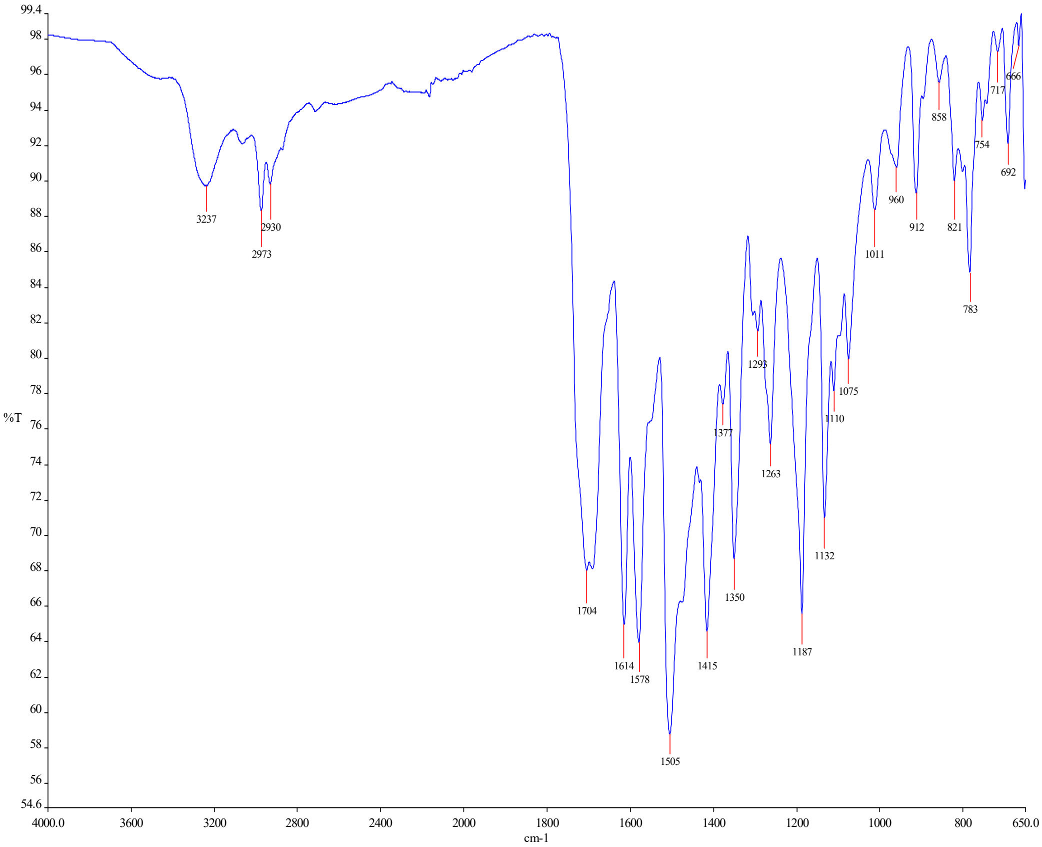

Figure S50. ^{13}C NMR spectra of compound **12f**

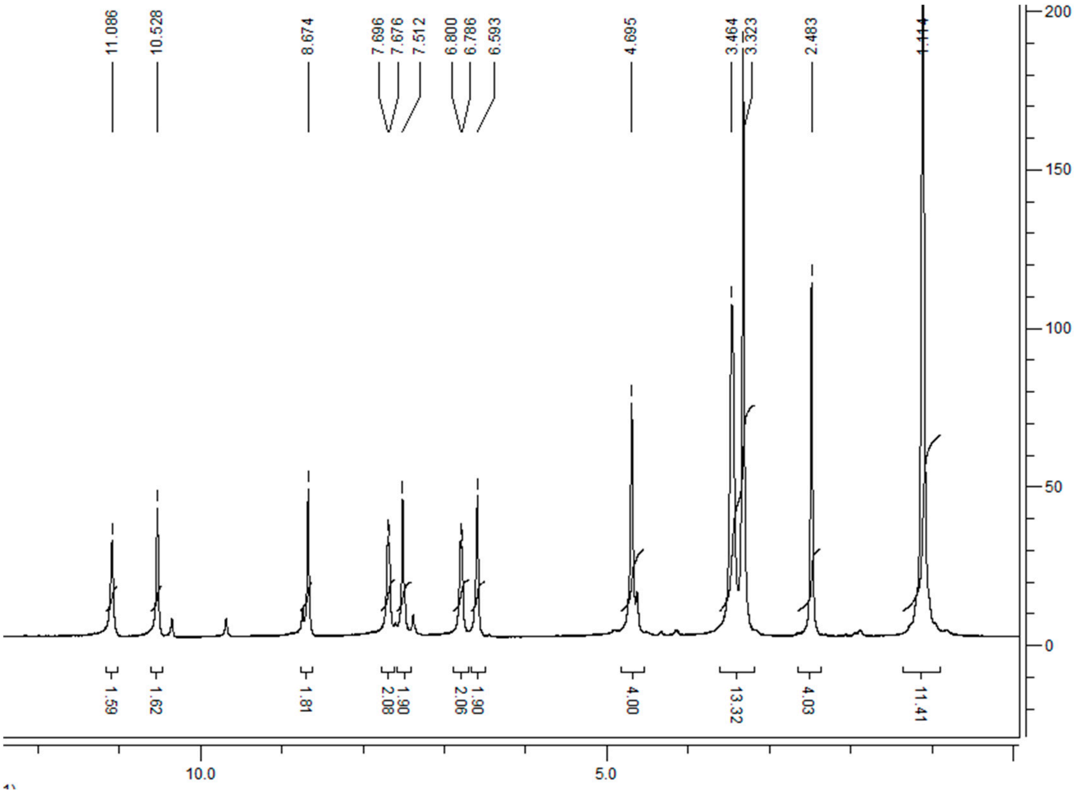

Figure S51. ^{1}H NMR spectra of compound **12f**

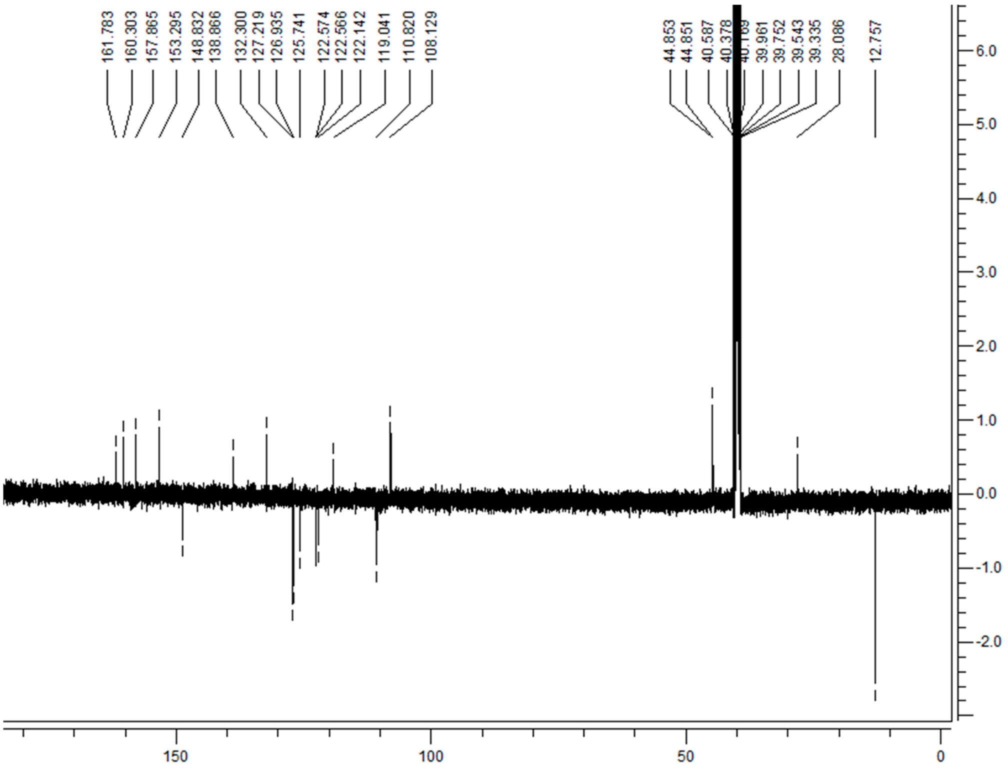

Figure S52. ^{13}C NMR spectra of compound **12f**
